# A neurodegenerative disease landscape of rare mutations in Colombia due to founder effects

**DOI:** 10.1186/s13073-022-01035-9

**Published:** 2022-03-08

**Authors:** Juliana Acosta-Uribe, David Aguillón, J. Nicholas Cochran, Margarita Giraldo, Lucía Madrigal, Bradley W. Killingsworth, Rijul Singhal, Sarah Labib, Diana Alzate, Lina Velilla, Sonia Moreno, Gloria P. García, Amanda Saldarriaga, Francisco Piedrahita, Liliana Hincapié, Hugo E. López, Nithesh Perumal, Leonilde Morelo, Dionis Vallejo, Juan Marcos Solano, Eric M. Reiman, Ezequiel I. Surace, Tatiana Itzcovich, Ricardo Allegri, Raquel Sánchez-Valle, Andrés Villegas-Lanau, Charles L. White, Diana Matallana, Richard M. Myers, Sharon R. Browning, Francisco Lopera, Kenneth S. Kosik

**Affiliations:** 1 Neuroscience Research Institute and Department of Molecular Cellular and Developmental Biology, University of California, Santa Barbara, CA USA; 2 Grupo de Neurociencias de Antioquia, School of Medicine, Universidad de Antioquia, Medellín, Colombia; 3 HudsonAlpha Institute for Biotechnology, Huntsville, AL USA; 4 Instituto Neurológico de Colombia (INDEC), Medellín, Colombia; 5 Department of Internal Medicine, School of Medicine, Universidad del Sinú, Montería, Colombia; 6 Department of Neurology, School of Medicine, Universidad de Antioquia, Medellín, Colombia; 7 Banner Alzheimer’s Institute, Phoenix, AZ USA; 8 Laboratorio de Enfermedades Neurodegenerativas (Fleni-CONICET), Buenos Aires, Argentina; 9 Centro de Memoria y Envejecimiento (Fleni-CONICET), Buenos Aires, Argentina; 10 Alzheimer’s Disease and Other Cognitive Disorders Unit, Hospital Clínic de Barcelona, IDIBAPS and University of Barcelona, Barcelona, Spain; 11 Neuropathology Section, Department of Pathology, University of Texas Southwestern Medical Center, Dallas, TX USA; 12 Instituto de Envejecimiento, Department of Psychiatry, School of Medicine, Pontifical Xaverian University, Bogotá, Colombia; 13 Department of Mental Health, Hospital Universitario Santa Fe de Bogotá, Bogotá, Colombia; 14 Department of Biostatistics, University of Washington, Seattle, WA USA

**Keywords:** Founder effect, Bottleneck, Admixture, Genetic drift, Selection, Demography, Neurodegeneration, Alzheimer’s disease, Frontotemporal dementia, Motor neuron disease

## Abstract

**Background:**

The Colombian population, as well as those in other Latin American regions, arose from a recent tri-continental admixture among Native Americans, Spanish invaders, and enslaved Africans, all of whom passed through a population bottleneck due to widespread infectious diseases that left small isolated local settlements. As a result, the current population reflects multiple founder effects derived from diverse ancestries.

**Methods:**

We characterized the role of admixture and founder effects on the origination of the mutational landscape that led to neurodegenerative disorders under these historical circumstances. Genomes from 900 Colombian individuals with Alzheimer’s disease (AD) [*n* = 376], frontotemporal lobar degeneration-motor neuron disease continuum (FTLD-MND) [*n* = 197], early-onset dementia not otherwise specified (EOD) [*n* = 73], and healthy participants [*n* = 254] were analyzed. We examined their global and local ancestry proportions and screened this cohort for deleterious variants in disease-causing and risk-conferring genes.

**Results:**

We identified 21 pathogenic variants in AD-FTLD related genes, and *PSEN1* harbored the majority (11 pathogenic variants). Variants were identified from all three continental ancestries. *TREM2* heterozygous and homozygous variants were the most common among AD risk genes (102 carriers), a point of interest because the disease risk conferred by these variants differed according to ancestry. Several gene variants that have a known association with MND in European populations had FTLD phenotypes on a Native American haplotype. Consistent with founder effects, identity by descent among carriers of the same variant was frequent.

**Conclusions:**

Colombian demography with multiple mini-bottlenecks probably enhanced the detection of founder events and left a proportionally higher frequency of rare variants derived from the ancestral populations. These findings demonstrate the role of genomically defined ancestry in phenotypic disease expression, a phenotypic range of different rare mutations in the same gene, and further emphasize the importance of inclusiveness in genetic studies.

**Supplementary Information:**

The online version contains supplementary material available at 10.1186/s13073-022-01035-9.

## Background

The circumstances related to Latin America’s unique demographic history led to numerous genetic founders that expanded rare genetic variation. The regional populations of Colombia originated from varying proportions of a recent tri-continental admixture consisting of diverse indigenous peoples, Spanish invaders, and enslaved Africans, all of whom had been geographically separated for tens of thousands of years. During the Spanish conquest, these individuals suffered massive mortality from numerous infectious diseases, including smallpox, influenza, syphilis, hepatitis, measles, encephalitis, tuberculosis, diphtheria, cholera, typhus, scarlet fever, and meningitis, which created a narrow bottleneck with a minimum effective population size approximately 12 generations ago [1]. Survivors were geographically dispersed in a patchwork of relatively isolated small founder populations. Following the first decades of the Spanish invasion and European expansion throughout various territories, the second half of the sixteenth century saw a large and continuous growth of an admixed population, especially in the Andean region of the country (Additional file 1: Figure S1). The population growth amplified the effects of genetic drift confined to highly local settings that marked a fine-grained geographic map with a local genetic stamp [2].

Demographic history and local ancestry have gained significant interest in genomic studies aiming to understand the disease burden of underrepresented populations and transferability of risk scores from research done in European cohorts. However, most of these studies have focused on genome wide association studies (GWAS) and polygenic risk scores that usually rely on the sequencing of common genetic variants [3–5], while missing those rare alleles absent from European genomes [6]. Rare variants are likely to play a role in the problem of “missing heritability,” have larger effect sizes [7], and are more susceptible to population dynamics and genetic drift.

Rare mutations contribute to the occurrence of neurodegenerative disease, which prompted a search for individuals with young onset familial dementia and related neurodegenerative disorders. We suspected that genetic drift stamped local populations with unique sets of rare variants. Numerous rare genetic conditions converge under this phenotypic label, and therefore as a population indicator of rare variation, dementia represents a readily identifiable trait with a great deal of genetic variation. Among the many genes in which disease mutations fit the phenotypic label are *PSEN1* [MIM: 104311], *PSEN2* [MIM: 600759], *APP* [MIM: 104760], *C9orf72* [MIM: 614260], *GRN* [MIM: 138945], *MAPT* [MIM: 157140], *TARDBP* [MIM: 605078], *FUS* [MIM: 137070], *VCP* [MIM: 601023], *CHMP2B* [MIM: 609512], and *TBK1* [MIM: 604834] [8]. Rare variants in these genes offer novel perspectives on the breadth of their associated clinical phenotypes and the underlying molecular pathways. Here, we describe a cohort of 900 Colombian individuals with neurodegenerative diseases and report the genetic variants associated with neurodegeneration in the context of their ancestral origins and admixture.

## Methods

### Subjects

Participants were recruited or referred to the “Grupo de Neurociencias de Antioquia,” University of Antioquia, Colombia for “The Admixture and Neurodegeneration Genomic Landscape” (TANGL) study. The project was approved by the Institutional Review Board (IRB) of the Medical Research institute, School of Medicine, Universidad de Antioquia. Written informed consent following the guidelines of the Code of Ethics of the World Medical Association, Helsinki declaration, and Belmont Report was obtained from all participants or their legally authorized proxies. The recruitment targeted patients with early-onset dementia and families in which multiple first-degree relatives were affected. All the individuals were born in Colombia (Additional file 1: Figure S1). All subjects were evaluated following a standard protocol including physical and neurological examination, as well as population validated neuropsychological assessment [9, 10]. Family history was obtained from the patients and their relatives and was considered positive if at least one first or second degree relative presented dementia or motor neuron disease (MND). Families were classified as autosomal dominant if at least three first degree relatives suffered from dementia or MND in two consecutive generations. When patients had familial forms of dementia, their relatives with neurological and psychiatric disorders were recruited along with healthy family members. Nine hundred individuals from 566 families with high quality genomes were used for analyses (genetic sequencing and quality control procedures are detailed in the Genome Sequencing methods).

Based on their clinical diagnosis, participants were divided in four cohorts:

- The Alzheimer’s disease (AD) [MIM: 104300] cohort (*n* = 376) included individuals with early-onset AD (AAO ≤ 65 years) and individuals with autosomal dominant late onset AD. Patients with atypical presentations of AD, such as primary progressive aphasia–logopenic variant (lvPPA), posterior cortical atrophy, and spastic paraparesis associated with *PSEN1* pathogenic variants [MIM: 607822] were included in this cohort. AD was diagnosed according the NINCDS-ADRDA criteria [11].
- The frontotemporal lobar degeneration and motor neuron disease (FTLD-MND) spectrum cohort (*n* = 197) comprised patients with multiple presentations of frontotemporal lobar degeneration (FTLD) [MIM: 600274], which include behavioral variant of frontotemporal dementia (bvFTD), primary progressive aphasia-semantic variant (svPPA), primary progressive aphasia-non-fluent/agrammatic variant (navPPA), and FTLD with amyotrophic lateral sclerosis (FTLD-ALS). Diagnosis of FTLD variants was done according to Gorno-Tempini et al. 2011 [12] and Rascovsky et al. 2011 [13]. Patients with cortico-basal degeneration (CBD), progressive supranuclear palsy (PSP) [MIM: 601104] diagnosed according to The Movement Disorder Society Criteria [14], and with amyotrophic lateral sclerosis (ALS) [MIM: 105400], diagnosed according to Strong et al. 2017 [15], were included in this cohort.
- The early-onset dementia not otherwise specified (EOD) cohort (*n* = 73) included patients with early-onset dementia (AAO ≤ 65 years) that did not fully meet criteria for AD or FTLD at the time of evaluation and did not have secondary causes that explain their neurodegeneration. Some of these individuals were relatives of the patients from the other cohorts but presented with conditions such as Parkinson’s disease [MIM: 168600], bipolar disorder [MIM: 125480], or Lewy body disease [MIM: 127750].
- The Healthy participant cohort (*n* = 254) included individuals related and unrelated to the patients. These subjects had a Clinical Dementia Rating (CDR) score of 0 in their last examination and no evidence of neurodegenerative dementia or motor neuron disease.

The complete demographic information of the 900 individuals can be found in Table 1, Additional file 2: Table S1 and Additional file 3: Table S2.

**Table 1:** Demographic information of the included cohorts

Cohort	*n*	AAO		Female		APOE genotype no. (%)											
						ϵ2/ϵ2		ϵ2/ϵ3		ϵ2/ϵ4		ϵ3/ϵ3		ϵ3/ϵ4		ϵ4/ϵ4	
		Mean	Range	*n*	%	*n*	%	*n*	%	*n*	%	*n*	%	*n*	%	*n*	%
AD	376	59	30-90	249	66.2	-	-	15	4	4	1.1	168	45	139	37.1	49	13.1
FTLD-MND	197	58.8	21-82	92	46.7	1	0.5	18	9.1	-	-	122	62	49	24.9	7	3.6
EOD	73	54.5	25-75	49	67.1	-	-	2	2.7	-	-	39	53	20	27.4	12	16.4
Healthy	254	60	18-100^a^	159	62.6	2	0.8	25	9.8	1	0.4	159	45	61	23.9	4	1.6
				549	60.7	3	0.3	60	6.7	5	0.6	488	54.2	269	29.9	72	8

*AD* Alzheimer’s disease, *FTLD-MND* frontotemporal lobar degeneration and motor neuron disorder, *EOD* early-onset dementia not otherwise specified, *AAO* age at onset

^a^ Age at evaluation. There were three Individuals with uncalled *APOE* genotype (one from AD cohort and two healthy individuals)

### Genome sequencing

Peripheral blood from the participants was obtained by standard phlebotomy, and genomic DNA was isolated from leukocytes using the Gentra Puregene Blood Kit (Qiagen). Genome sequencing (WGS) was performed at the HudsonAlpha Institute for Biotechnology on either the Illumina HiSeq X platform, or the Illumina NovaSeq platform. A subset of individuals was sequenced at the Human Longevity Institute on the Illumina HiSeq X platform (119 samples). The combined dataset had a mean read depth of 34X and an average of 92% of bases covered at 20X. Sequencing libraries at HudsonAlpha were prepared by Covaris shearing, end repair, adapter ligation, and PCR using standard protocols. Library concentrations were normalized using KAPA qPCR prior to sequencing. Sequencing reads from both centers were aligned to the hg19 reference genome with bwa-0.7.12 [16]. BAMs were sorted and duplicates were marked with Sambamba 0.5.4 [17]. Indels were realigned, bases were recalibrated, and gVCFs were generated with GATK 3.3 [18]. Variants were called across all samples in a single batch with GATK 3.8 using the -newQual flag to minimize false negative singleton calls. The recall rate for GATK against truth sets is between 93 and 99% for single nucleotide variants and 85 and 98% for small (less than 50 bp) indel events [19]. Genome annotation was performed using SnpEff 4.3 [20] after splitting multi-allelic sites with Vt [21]. The genome was annotated with the gene definitions from human genome build Ensembl GRCh37.75 [22]. All single nucleotide variants and indels were annotated with CADD v1.3 [23]. Population database frequency annotations included 1000 Genomes Phase 3 (1000GP) [24], TOPMed Bravo [25] (lifted over from hg38 to hg19 using CrossMap 0.2.7 [26]), and several population database sets annotated using WGSA 0.7 [27] including ExAC [28], gnomAD [29], ESP [30], and UK10K [31]. Variants were also annotated with dbSNP release 151 [32].

Calls were filtered with vcftools (v0.1.12b) [33] to retain sites with quality scores equal or greater than 20 and mean read depth scores equal or greater than 30. KING (v2.2.4) [34] was used to verify disclosed familiar relationships and pedigree structures, and individuals with unexplained relatedness were removed. For duplicate samples and monozygotic twin pairs, only one genome was kept. PLINK v.1.90 [35, 36] was used to identify and exclude individuals with discordant X-chromosome sex and those with more than 5% missing data [37]. Mendel errors were set to missing before removing autosomal variants with missingness > 5% obtaining a total of 41,123,431 variants and 900 individuals from 566 families available for analysis (Additional file 1: Figure S2).

To compare the TANGL genomes to previously identified carriers of *PSEN1* c.428T>C (p.Ile143Thr) [38] from Colombia and *PSEN1* c.356C>T (p.Thr119Ile) from Colombia and Argentina [39], we sequenced additional individuals using the Array-8+ v1.0 Kit + neuro booster array consortium (NBA) content, beadchip 20042459 Illumina Global Diversity (Catalog 20031816). Imputation was performed using the TOPMed Imputation Panel and Server (version 1.3.3) [40], which includes 97,256 references samples and 308,107,085 variants and uses Minimac4 for imputation. Pre-imputation scripts (version 4.3.0 from William Rayner at the University of Oxford) were run using default settings, which filtered out palindromic single nucleotide variants (SNVs) with minor allele frequency (MAF) > 0.4 or variants with > 0.2 MAF difference from the TOPMed reference panel [41]. The Colombian carriers of these *PSEN1* variants had been recruited and evaluated by the Grupo de Neurociencias de Antioquia (GNA). The Argentinian sample was provided by the Neurodegenerative illnesses’ laboratory (Fleni-CONICET). The clinical assessment and sequencing of these individuals was done with written informed consent and approved by the IRB of the Medical Research Institute School of Medicine, Universidad de Antioquia, and the IRB from “Instituto de Investigaciones Neurológicas Raúl Carrea – FLENI.”

To compare the TANGL genomes to previously identified carriers of *MAPT* c.1189C>T (p.Pro397Ser) from Spain, we obtained exome sequencing data from an individual previously sequenced by the Alzheimer's disease and other cognitive disorders unit at Hospital Clínic de Barcelona. The exome from the Spanish c.1189C>T (p.Pro397Ser) carrier [42] was processed from fastq to VCF using a standard clinical alignment pipeline from the HudsonAlpha Institute for Biotechnology Clinical Services Laboratory that uses Sentieon version 201808.07 (a computational wrapper for common tools such as bwa), including alignment with Sentieon-BWA (version 201808.07; identical to bwa mem 0.7.15-r1140) and variant calling with Illumina Strelka2 (version 2.9.10) [43]. The use of this sample was approved by the IRB from the “Hospital Clinic de Barcelona.”

### Population structure analysis

We implemented protocols similar to those previously developed for ancestry estimation in admixed populations [3, 44]. We merged the 900 genomes (TANGL cohort) with the 1000 Genomes Project (1000GP) Phase 3 genomes generating the TANGL.1000GP dataset (*n* = 3404). Then, we created a subset including only the TANGL cohort, the non-admixed African Populations (AFR), *N* = 504, and European populations (EUR), *N* = 503. We merged these genomes with Native American samples (NAT), *N* = 43 from Mao et al. [45] inferred to have > 0.99 Native Ancestry, and created the TANGL.AFR.EUR.NAT dataset. After removing monomorphic variants, triallelic sites that were not due to a strand flip in either dataset and those sites with missingness greater than or equal to 1%, we retained 845,950 autosomal variants and 1950 individuals for further analysis.

### Global ancestry inference

A subset of unrelated samples from TANGL.AFR.EUR.NAT was selected by keeping only the proband of each family and, using KING (v2.2.4) [34] with “—related” and “--degree 3” settings to identify cryptic relatedness. Only sample pairs with kinship coefficient less than 0.044 were retained for TANGL, AFR and EUR. The NAT individuals showed significant relatedness between them, and the threshold for that population was set to “—degree 2” to retain the most NAT samples with kinship less than 0.0884. The final TANGL.AFR.EUR.NAT -Unrelated dataset comprised 1611 unrelated individuals (TANGL *N* = 566, AFR *N* = 501, EUR *N* = 503, NAT = 41).

We calculated global ancestry using ADMIXTURE (v.1.3.0) [46] independently for the unrelated TANGL individuals (*n* = 566) and for the TANGL.AFR.EUR.NAT-Unrelated cohort. As recommended by ADMIXTURE, PLINK (v.1.9) [35, 36] was used to perform pair-phased linkage disequilibrium (LD) pruning; excluding variants with an r^2^ value of greater than 0.2 with any other SNP within a 50-SNP sliding window, advancing by 10 SNPs each time (--indep-pairwise 50 10 0.2). The LD-pruned dataset contained 203,810 variants. We then performed an unsupervised analysis modeling from one to ten ancestral populations (*K* = 1–10) using the random seed option and replicating each calculation 20 times. We selected the run with the best Loglikehood value for each *K* and compared the cross validation (cv) error values to determine the model with the lowest cv value. Ancestral proportion statistics of mean and standard deviation were calculated using the statistical software R [47].

In addition, we determined mitochondrial and Y-chromosome haplogroups of the TANGL-unrelated cohort using HaploGrep 2 with Phylotree 17 [48], and yHaplo respectively [49].

### Local ancestry inference

We phased the combined TANGL.AFR.EUR.NAT dataset with SHAPEIT (v.2.r900) [50] using the haplotype reference panel of the 1000GP. We used the parameters –duohmm and a window of 5 MB (-W 5), which takes advantage of the inclusion of families, pedigree structure, and the large amount of IBD shared by close relatives, leading to increased accuracy [51]. We used the PopPhased version of RFMix (v1.5.4) [52] to estimate the local ancestry using the following flags: -w 0.2, -e 1, -n 5, --use-reference-panels-in-EM, --forward-backward as recommended by Martin et al. [3] for estimating local ancestry in admixed populations. To determine the carrier haplotype and local ancestry of a rare variant of interest, we used PLINK (v.1.9) [35, 36]. We identified other single nucleotide variants (SNVs) in linkage disequilibrium with the variant of interest and used them as tags to identify the carrier haplotypes in the phased dataset, and then searched for the local ancestry of the specific locus in the RFMix output.

### Principal component analysis (PCA)

For PCA, we used the subset of unrelated samples with LD-pruning of variants as described in the methods for “Global ancestry inference.” We performed a PCA using the *smartpca* package from EIGENSOFT (v7.2.1) [53], with 3 outlier removal iterations (numoutlieriter: 3) and flag “altnormstyle: NO” to match EIGENSTRAT normalization formulas [53]. The PCA results were plotted using the PCAviz package [54] for R. For the PCA with the Ancestral populations, we retained variants with MAF > 10%. For the PCA of the TANGL-unrelated cohort, we extracted a common variant set, retaining those with MAF > 10%, and then a lower frequency variant set, keeping only variants with MAF between 5 and 10%.

### Genetic screening for disease causing variants

Each individual was initially screened for pathogenic variants in the most recognized genes associated with AD and FTLD according to AD/FTLD mutation databases (https://www.molgen.vib-ua.be/ADMutations, https://www.alzforum.org/mutations); *PSEN1*, *PSEN2*, *APP*, *MAPT*, *GRN VCP*, *FUS, CHMP2B*, *TARDBP*, and *TBK1* (the molgen.vib-ua.be/ADMutations database is not available as of July 2021). For the present study, the terms “pathogenic” and “likely pathogenic” refer to variants that are both predicted to be disruptive or damaging to the protein function and causative for a disease according ACMG criteria [55].

A secondary genetic analysis was done to identify pathogenic and likely pathogenic variants in other genes associated with similar or overlapping phenotypes. For the secondary screening, we chose the disease-causing genes reported in the following OMIM phenotypic series and phenotypes: frontotemporal dementia and/or amyotrophic lateral sclerosis [MIM: PS105550, PS167320, PS105400], Parkinson disease [MIM: PS168600], adult-onset leukoencephalopathies [MIM: PS125310, 221820], and ceroid lipofuscinoses [MIM: PS256730]. We retained variants with MAF of 0.001 or less in the ExAC database if the gene had autosomal dominant or X-linked inheritance, and 0.01 or less if the gene had autosomal recessive inheritance. The remaining variants were discarded if they were more prevalent in controls than cases or if they had a CADD Phred score less than 20. The selected protein altering variants defined as nonsynonymous single nucleotide variants, splicing altering variants, insertions, or deletions were manually curated by searching in the databases described before as well as ClinVar [56] and LitVar [57]. The previously unreported (novel) variants were classified according to the guidelines published by the American College of Medical Genetics and Genomics and the Association for Molecular Pathology [55]. Variants in *PSEN1* and *PSEN2* were also classified according the Guerreiro algorithm [58]. Additionally, subjects were screened for *C9ORF72* [MIM: 614260] hexanucleotide expansion using repeat-primer following the protocol described in DeJesus-Hernandez et al. [59] because, while *C9ORF72* expansions are possible to detect from short-read PCR-free genomes [60], such events are not detectable from PCR positive genomes which were conducted here. We searched for large copy number variations using four callers: DELLY [61], ERDS [62], CNVnator [63], and BIC-seq2 [64]. Events called by multiple callers were inspected for validity using Integrative Genomics Viewer [65]. In contrast to GATK small variant calls, where recall rates against truth sets are known, there are not recall rates available for this employed combination of tools, though we note that there is a high false negative rate for all CNV callers from short read PCR-positive genome data; thus, the goal in CNV analysis was to have high confidence in those variants that were identifiable across all four callers at the expense of missing some true positives that may not pass these strict criteria. Better detection of expansions such as *C9ORF72* or heretofore unidentified similar events and/or better large indel detection will be aided by emerging use of long read sequencing which can help identify events that would be missed otherwise [66].

Neuropathologic assessment of *CSF1R* c.2068G>A (p.Gly690Ser) and *DNAJC5* c.347 T>G (p.Leu116Arg) carriers was performed at the Brain Bank of the Neuroscience Group of Antioquia following standardized protocols [67, 68]. Tissues were stained with hematoxylin-eosin, Luxol Fast blue, and periodic acid–Shiff (PAS). The brain donation and neuropathologic assessment were done with written informed consent and approved by the IRB of the Medical Research Institute School of Medicine, Universidad de Antioquia.

### Genetic screening for risk associated variants

We used publications in the literature to identify genes in which rare variants were associated with increased risk for AD and/or FTLD-MND with an odds ratio higher than 2. *TREM2* [69, 70] [MIM: 605086], *ABCA7* [69, 71, 72] [MIM: 107741], and *SORL1* [69, 73] [MIM: 602005] were selected as intermediate effect risk genes. We retained variants that were known to be risk conferring, led to premature truncation of the protein (PTV), or that were classified as strictly damaging (SD) according to previous published criteria [69]. Strictly damaging variants had MAF ≤ 0.01 in the ExAC database and were unanimously classified as deleterious by three different in silico prediction algorithms; SIFT [74], Polyphen-2 (Hum Div.) [75], and MutationTaster [76]^.^ In addition to this strategy, we included *ADAM10* [MIM: 602192] c.510G>T (p.Gln170His) and c.541A>T (p.Arg181Gly) variants as they have been reported to confer intermediate risk for AD [77, 78]. Variant nomenclature is according to the Human Genome Variation Society Recommendations [79]; the GenBank reference transcripts used for each disease causing and risk conferring variant can be found in Additional file 4: Table S3.

### Identity by descent

If any of the disease-conferring or risk-associated variants were shared by two or more unrelated individuals, we used hap-IBD [80] v1.0 to search for identity by descent (IBD) around the locus. Because this software detects IBD of 2 cM and higher, we additionally performed an alignment of the haplotypes carrying the variants of interest to search for smaller IBD segments between the TANGL and 1000 Genomes Project (1000GP) carriers. Autozygosity (homozygosity by descent) was determined using the same methods. Code and scripts used for the population structure and identity by descent analyses are publicly available [81].

## Results

### Population analysis of the genomes from the neurodegeneration cohort

Nine hundred Colombian individuals with high-quality genome sequences were included in “The Admixture and Neurodegeneration Genomic Landscape” (TANGL) study. The individuals were divided into four different cohorts: Alzheimer’s disease (AD), frontotemporal lobar degeneration and motor neuron disease (FTLD-MND), early-onset dementia not otherwise specified (EOD), and healthy participants (Table 1 and Additional file 2: Table S1). These 900 individuals represented 566 independent families, which were classified into the same four cohorts according to the diagnosis of the proband (Additional file 3: Table S2).

**Fig. 1:**
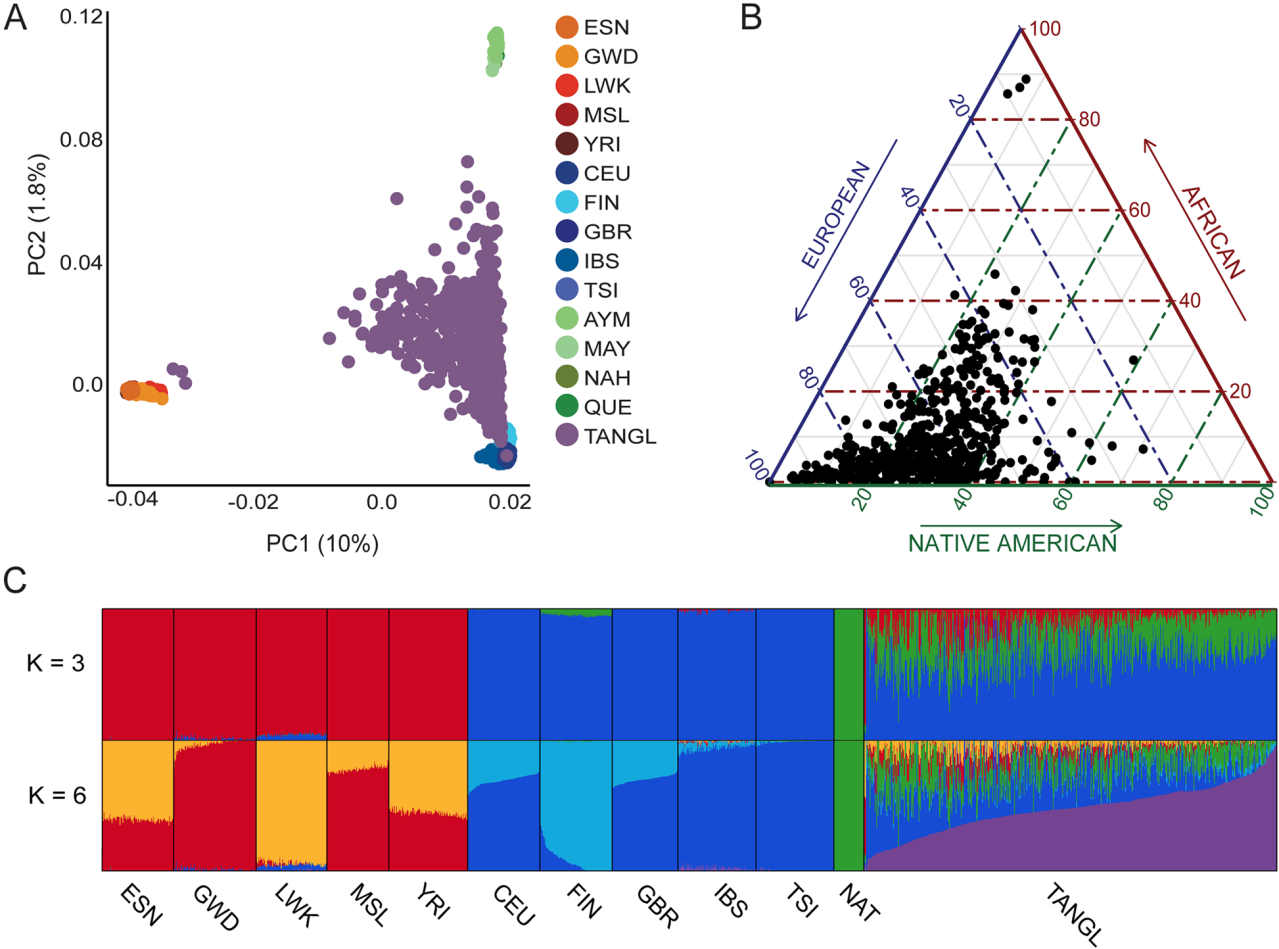
Population structure and admixture analyses of the TANGL cohort. **A** PC1 vs PC2 of the PCA of the TANGL cohort (purple) with the European (blue) and African (orange) individuals from the 1000GP and 43 Native American genomes (green). **B** Ternary plot representing the global ancestry of each of the individuals in the TANGL cohort values according to sum of local ancestries calculated by RFMix. **C** Q plot of ADMIXTURE results assuming 3 and 6 ancestral populations (K). ESN: Esan in Nigeria. GWD: Gambian in Western Divisions in the Gambia. LWK: Luhya in Webuye, Kenya. MSL: Mende in Sierra Leone. YRI: Yoruba in Ibadan, Nigeria. CEU: Utah Residents (CEPH) with Northern and Western European Ancestry. FIN: Finnish in Finland. GBR: British in England and Scotland. IBS: Iberian Population in Spain. TSI: Toscani in Italia. AYM: Aymara. MAY: Mayan, NAH: Nahuatl. QUE: Quechua. NAT: Native American

Because the sample set was highly selected, we first sought to determine the genomic similarity between the TANGL cohort and other Colombian individuals. We initially merged the TANGL and the 1000 Genomes Project (1000GP) phase 3 [82] datasets and performed a principal component analysis (PCA). The TANGL cohort had a similar distribution in the first three principal components (PC) to the “Colombians from Medellín” (CLM) of the 1000GP, allowing us to conclude that both populations are genetically similar (Additional file 1: Figure S3). To take a closer look into the ancestral origins of the TANGL cohort, we used the software ADMIXTURE to estimate the number of ancestral populations (*K*) from which the cohort arose. The lowest cross validation (cv) error was obtained when assuming the cohort was derived from three ancestral populations (*k* = 3), which agrees with the history of the tri-continental admixture after the Spanish conquest (Additional file 1: Figure S4). To analyze the global and local ancestry of the TANGL cohort, we merged the TANGL genomes with the European and African populations from the 1000GP and Native American genomes from Mao et al. [45] and repeated the ADMIXTURE analysis. In this joint dataset, *K* = 3 accurately differentiated Native American, European and African cohorts, but the lowest CV error was obtained for *K* = 6 (Fig. 1 and Additional file 1: Figure S5). Modeling for six ancestral populations allowed the detection of substructure within the African and European cohorts and created an additional cluster described by Moreno-Estrada et al. [44] as a “Latino-specific European component.” Consistent with previous studies [83], the ancestral population with the highest proportion in our cohort was European (mean of 64%, SD = 15%), followed by Native American (mean of 27%, SD = 11%), and African being the least represented (mean of 9%, SD = 11%) (Additional file 1: Figure S6). These individual admixture values (*Q*-values) at *K* = 3 correlated with the sum of local ancestries estimated by RFMix (Pearson’s *r* > 0.99), allowing us to conclude that the local ancestry inferred for each individual matches the percentages of global ancestry obtained by an orthogonal method) (Additional file 1: Figure S7). However, the regional differences in the fine structure of the Colombian population make these global ancestry proportions highly region dependent. For example, the three individuals whose global ancestry was nearly 90% African were from the Pacific coast of the country where former enslaved Africans settled and most of the population self identifies as Afro-Colombian (Additional file 1: Figure S1).

After calculating the proportions of global ancestry, we evaluated the TANGL cohort for sex biased admixture, a genetic trait previously described in the Colombian population [84, 85]. We used HaploGrep2 and yHaplo to determine mitochondrial and Y-chromosome haplogroups. The mitochondrial haplogroups of the probands (*n* = 566) were predominantly Native American (83.4%) while the Y-chromosome haplogroups (*n* = 224) were mostly of European and of Mediterranean origins (92.8%), thus supporting the conclusion than multiple cohorts of Colombian origin show sex-biased admixture with Native American maternal lineages and paternal lineages from Europe (Additional file 5: Table S4 and Additional file 6: Table S5). Overall, these analyses let us conclude that despite recruiting the TANGL cohort based upon neurodegenerative conditions from the Andes region of Colombia, it recapitulated the admixture patterns previously described in the country.

The TANGL cohort was distributed between the three ancestral populations in the PCA, clustering closer to Europeans and Native Americans. To determine if the clustering of the admixed individuals was driven by their percentages of global ancestry, we compared the values of the principal components (PC) with the percentage of global ancestry attributed to each of the three ancestral populations by ADMIXTURE. PC1 correlated with the percentage of African ancestry (Pearson’s r2 of 1), and PC2 showed a correlation with the level of Native American ancestry (Pearson’s r2 of 0.87) (Additional file 1: Figures S8, S9 and S10). To determine whether the Colombian population clustered according to their global ancestry without including the ancestral populations in the analyses, we retained the 566 unrelated probands from the TANGL cohort and performed two PCAs, one with common variants (MAF > 10%) and one with less frequent variants (MAF 5-10%). Both PCAs showed correlation of the PCs with the global admixture proportions, regardless of the inclusion of the ancestral population (Additional file 1: Figures S11, S12 and S13).

### Neurodegenerative disease variants in the TANGL cohort

#### AD-associated genes

The 900 genomes were initially examined for variants in AD-associated genes (*PSEN1*, *PSEN2*, and *APP*), and the protein altering variants were curated according to the ACMG guidelines for the interpretation of genetic variants [55] and the algorithm proposed by Guerreiro et al. [58] to determine pathogenicity (Additional file 1: Figures S14, S15 and Additional file 7: Supplementary methods).

Eleven deleterious variants were identified in the *PSEN1* gene (Table 2 and Additional file 8: Table S6 and Additional file 9: Table S7). Three of these were novel; two classified as definite pathogenic, c.485 T>G (p.Ile162Ser) c.667C>A (p.Gln223Lys); and one as probably pathogenic according to the Guerreiro algorithm, c.782C>T (p.Val261Ala). Four of these *PSEN1* variants had been previously identified in the Colombian population c.349C>G (p.Pro117Ala), c.428T>C (p.Ile143Thr), c.839A>C (p.Glu280Ala), and c.1247 T>C (p.Ile416Thr) [38, 86–88], and four had been described in families outside Colombia with diverse ancestries c.356C>T (p.Thr119Ile) [39], c.488A>G (p.His163Arg) [89], c.791C>T (p.Pro264Leu) [89] and c.851C>T (p.Pro284Leu) [90]. *PSEN1* c.839A>C (p.Glu280Ala) [86], of European origin, is the largest family in the world with familial Alzheimer’s disease and living nearby is a family with the *PSEN1* variant c.1247 T>C (p.Ile416Thr) [87] that originated in Africa.

**Table 2:** Pathogenic variants identified in disease causing genes

**Alzheimer disease genes**							
**Gene**	**Coding change**	**dbSNP/ClinVar**	**ExAC**	**SIFT**	**Polyphen**	**CADD**	**Local ancestry**
***APP***	g.(26253828_30011000)dup	SCV001751549	.	–	–	–	European
***PSEN1***	c.349C>G (p.Pro117Ala)	rs63750550	.	D	P	26.9	European
	c.356C>T (p.Thr119Ile)	rs1566630791	.	T	P	24.4	European
	c.428T>C (p.Ile143Thr)	rs63750004	.	D	D	26.8	European
	c.485T>G (p.Ile162Ser)	rs1898533739	.	D	D	32	Native American
	c.488A>G (p.His163Arg)	rs63750590	.	T	B	23.4	European
	c.667C>A (p.Gln223Lys)	rs1898776259	.	D	D	33	Native American
	c.782T>C (p.Val261Ala)	SCV001751539	.	D	P	25.9	Undetermined
	c.791C>T (p.Pro264Leu)	rs63750301	.	D	D	35	Native American
	c.839A>C (p.Glu280Ala)	rs63750231	.	D	D	29.3	European
	c.851C>T (p.Pro284Leu)	rs63750863	.	D	D	33	European
	c.1247T>C (p.Ile416Thr)	SCV001751540	.	D	P	25.9	African
***PSEN2***	c.487C>T (p.Arg163Cys)	rs200931244	.	D	D	35	African
**FTLD genes**							
**Gene**	**Coding change**	**dbSNP**	**ExAC**	**SIFT**	**Polyphen**	**CADD**	**Local ancestry**
***C9ORF72***	(GGGGCC)n Repeat Expansion	rs143561967		.	.	.	European
***GRN***	c.709-2A>G (p.Ala237fs)	rs63750548	.	.	.	23.1	European
***MAPT***	c.902C>T (p.Pro301Leu)	rs63751273	.	D	D	34	European
	c.1189C>T (p.Pro397Ser)	rs1295855402	.	D	D	25	European
***TARDBP***	c.881G>T (p.Gly294Val)	rs80356721	0.00000824	T	P	18.89	European
	c.1147A>G (p.Ile383Val)	rs80356740	0.00000865	T	B	0.308	European
***TBK1***	c.1257_1258del (p.Val421Cfs*26)	rs1392685429	.	.	.	.	European
	c.1717C>T (p.Arg573Cys)^+^	rs772820487	0.00003329	T	D	29.6	European
**ALS genes**							
**Gene**	**Coding change**	**dbSNP**	**ExAC**	**SIFT**	**Polyphen**	**CADD**	**Local ancestry**
***ANXA11***	c.904C>T (p.Arg302Cys)	rs142183550	0.0000412	D	D	31	Native American
***FIG4***	c.122T>C (p.Ile41Thr) ^+^	rs121908287	0.001	D	D	26.5	European
***HNRNPA2B1***	c.965G>A (p.Gly322Glu)	SCV001751542	.	D	D	23.6	Native American
***SOD1***	c.63C>G (p.Phe21Leu)	rs1555836170	.	T	D	22.9	Native American
***SQSTM1***	c.1175C>T(p.Pro392Leu)	rs104893941	0.0009	D	B	34	European
***TUBA4A***	c.820C>G (p.Pro274Ala)	rs1241875438	.	.	D	23.8	Native American
***TUBB4A***	c.811G>A (p.Ala271Thr)	rs587777074	0.000003992	.	P	22.8	Native American
***UBQLN2***	c.724G>A (p.Ala242Thr)	SCV001751543	.	D	D	25.9	Undetermined
**Other neurodegeneration associated genes**							
**Gene**	**Coding change**	**dbSNP**	**ExAC**	**SIFT**	**Polyphen**	**CADD**	**Local ancestry**
***CSF1R***	c.2068G>A (p.Gly690Ser)	rs141866247	0.0000165	T	D	23.1	Native American
***DNAJC5***	c.347T>G (p.Leu116Arg)	SCV001751544	.	D	P	27.2	African
***LRRK2***	c.4334C>G (p.Ser1445Cys)	rs1945001552	.	T	P	24.3	European

*ExAC* ExAC database minor allelic frequency. SIFT scores are D, deleterious, and T, tolerated. PolyPhen-2 scores are D, probably damaging, P, possibly damaging, and B, benign. CADD corresponds to the Phred score. Variants with + were identified in homozygous states. GenBank transcripts for each gene can be found in Additional file 4: Table S3

*PSEN1* c.782 T>C (p.Val261Ala) was identified in a singlet without confirmed paternity, and it was classified as likely pathogenic (ACMG criteria)/probably pathogenic (Guerreiro) despite the lack of family history due to the report of three different pathogenic mutations in the same codon c.780G>T (p.Val261Phe) [91], c.780G>A (p.Val261Ile) [92], andc.780G>C (p.Val261Leu) [93]. All the reported variants, except c.851C>T (p.Pro284Leu), presented as early-onset amnestic AD. The c.851C>T (p.Pro284Leu) carriers developed spastic paraparesis (SP), which is an atypical form of AD occasionally associated with certain *PSEN1* mutations [91, 94, 95]. All the families with pathogenic *PSEN1* mutations had autosomal dominant inheritance (Additional file 1: Figure S16); however, the singlet c.782 T>C (p.Val261Ala) was indeterminate. Among these *PSEN1* variants, six were of European origin, three were Native Americans, and one African (Table 2).

All the carriers of each variant, except c.791C>T (p.Pro264Leu), reported a known common ancestor (Additional file 1: Figure S16). Several families from the harbored the *PSEN1* c.791C>T (p.Pro264Leu) variant, but we could not connect them by family history. Therefore, to prove that c.791C>T (p.Pro264Leu) was the result of a founder effect, we used the hap-IBD software to identify identical by descent (IBD) segments between the variant carrying chromosomes. All the *PSEN1* c.791C>T (p.Pro264Leu) carrier haplotypes shared an IBD segment of 2.79 cM around the *PSEN1* locus, supporting the hypothesis of a common ancestor for all three families originating at about the same time (Additional file 1: Figure S17). *PSEN1* c.791C>T (p.Pro264Leu) has been described in multiple populations (France [89, 96–99], UK [100, 101], Turkey [102], and Japan [103]) suggesting that *PSEN1* c.791C>T (p.Pro264Leu) is a recurring mutation. While the European carriers of this variant often present SP [104], this phenotype was not observed in the Colombian carriers of the variant. To determine if this phenotypic heterogeneity is related to the ancestral haplotype wherein the variant arose, we used RFMix to estimate the ancestry of the variant carrier haplotype (Table 2 and S6). In the TANGL cohort, *PSEN1* c.791C>T (p.Pro264Leu) resided on a Native American haplotype, which suggests that the haplotype of origin may play a role in the different expressivity and clinical manifestations between the variant carriers. Six of the other pathogenic *PSEN1* variants resided on European haplotypes, two variants were present in Native American and one in an African background. The multi-ancestral origins of the *PSEN1* variants suggest that the admixture process contributed to the introduction of pathogenic variants to a population.

Two of the *PSEN1* variants described in this cohort had been previously identified in other families in Colombia [c.428T>C (p.Ile143Thr) [38], c.356C>T (p.Thr119Ile)], and in Argentina [c.356C>T (p.Thr119Ile) [39]]. We performed additional array genotyping to test for IBD between the members of these families and those from the TANGL cohort. The Colombian carriers of c.428T>C (p.Ile143Thr) and c.356C>T (p.Thr119Ile) showed IBD overlapping the *PSEN1* locus (Additional file 1: Figures S18 and S19). Interestingly, the Colombian individuals who harbored c.356C>T (p.Thr119Ile) with whom no shared ancestor could be determined by history carried a small IBD segment shared with the Argentinian carrier of the same variant (Additional file 1: Figure S20). The geographical expanse over which these variants reside could reveal small population migratory streams from Europe or within the South American continent.

In addition to the eleven pathogenic variants, we identified four benign variants in *PSEN1.* c.1279A>G (p.Ile427Val) and c.114C>A (p.His38Gln) that did not segregate with the illness, while c.118G>A (p.Asp40Asn) and c.953A>G (p.Glu318Gly) have been reported in cases and controls without a clear disease association [105–107]. Thus, most of the *PSEN1* missense variants in this cohort are pathogenic and have an age-dependent phenotype of amnestic AD. In contrast, the majority of the variants observed in *PSEN2* were either benign or had been previously classified as risk factors for AD. Only the variant c.487C>T (p.Arg163Cys), which had been described in a Chinese patient with AD [108], was classified as likely pathogenic (Additional file 1: Figure S21). Interestingly, this variant resided on an African haplotype in the Colombian carrier. No pathogenic variants were observed in *APP*; but one individual with AD had copy number variation (CNV) spanning *APP* [104] (chromosome 21 g.(26253828_30011000)dup, Additional file 1: Figure S22). These results confirm *PSEN1* as the most prevalent gene associated with genetic AD in our cohort, mostly as the result of founder effects, and that the current genetic burden of the TANGL cohort is influenced by the genetic diversity of its founders.

#### Variants in FTLD-MND associated genes

We performed the same curation process for FTLD-MND associated genes (*MAPT*, *C9ORF72*, *GRN*, *VCP*, *FUS*, *CHMP2B*, *TBK1*, *TARDBP*). Most of the individuals with genetic forms of FTLD-MND in the TANGL cohort had deleterious variants in *MAPT* and *TARDBP* (Table 2 and Additional file 8: Table S6 and Additional file 9: Table S7). The *MAPT* c.1189C>T (p.Pro397Ser) variant was identified in three independent families from the same geographic region that shared IDB segment of 2.89 cM overlapping the locus (Additional file 1: Figures S23 and S24). This variant had been previously reported in five apparently unrelated Spanish families [42], and like the Spanish counterpart, the Colombian *MAPT* c.1189C>T (p.Pro397Ser) carriers had variable expressivity of the illness (Additional file 9: Table S7 and Additional file 10: Table S8). To elucidate whether the Colombian *MAPT* c.1189C>T (p.Pro397Ser) carriers were IBD with the Spanish families, we used exome sequencing data from a Spanish patient to search for similarities in the variant carrying haplotype. We identified a minimal shared haplotype of 2.65 cM including the *MAPT* locus, which suggests that the Colombian families share a common ancestor with the Spanish carriers of *MAPT* c.1189C>T (p.Pro397Ser) (Additional file 1: Figure S25).

Two siblings with FTLD-MND born of consanguineous parents were homozygous for the *TBK1* c.1717C>T (p.Arg573Cys) variant (Additional file 1: Figure S26). Haploinsufficiency of *TBK1* has been previously associated with familial ALS and FLTD and is a known mechanism of pathogenicity [109]. Homozygosity of nonsense *TBK1* variants has been proven to be lethal in mice [110]. A second variant in *TBK1* was c.1257_1258del (p.Val421Cfs*26), identified in two unrelated individuals that shared an IBD segment of 3.1 cM including the *TBK1* locus (Additional file 1: Figure S27). We identified two variants in *TARDBP* that had been previously reported in European populations with diagnosis of ALS [111, 112], and in contrast with these cohorts, Colombian *TARDBP* c.1147A>G (p.Ile383Val) carriers had significant intra-familial variability with heterogeneous FTLD-MND spectrum disorders (Additional file 1: Figure S28). Our study identified only one carrier of *C9ORF72* expansion, a single carrier of a pathogenic variant in *GRN* (Additional file 1: Figure S29), and no disease-causing variants in *CHMP2B*, *FUS*, or *VCP*. While the frequency of the identified mutations differs from those reported in European descent cohorts [59, 113], all the identified pathogenic variants in these FTLD-MND associated genes resided on European haplotypes.

#### Other genes associated with ALS in the cohort

To explore the phenotypic and genetic overlap between FTLD and ALS, we searched for deleterious variants in nineteen additional genes associated with ALS, with or without FTLD (Additional file 1: Figure S14, S15 and Additional file 7: Supplementary methods). The *SQSTM1* [MIM: 601530] c.1175C>T (p.Pro392Leu) variant was present in 11 unrelated cases and two controls of the TANGL cohort. These cases were unrelated and were clinically heterogeneous: six had diagnosis of AD, three of FTLD, one of CBD, and one PSP (Table 2 and Additional file 8: Table S6). Eight of the eleven cases had family history of dementia or neurodegenerative disease, and none of them carried other pathogenic mutations in the explored disease-causing genes. This variant was initially reported in European individuals with familiar forms of FTLD, Paget’s disease of the bone, and ALS [114–116]. Later studies identified this variant both in cases and controls, suggesting that it may be a risk factor rather than causal for illness [117, 118].

The *SQSTM1* c.1175C>T (p.Pro392Leu) is the result of founder effects in Belgian, Dutch, and Spanish individuals [119], and it was present in five individuals from the European cohort of the 1000GP. We used HAP-IBD to search for IBD between the Colombian and the 1000GP carriers of *SQSTM1* c.1175C>T (p.Pro392Leu). Ten carriers of the TANGL cohort shared IBD segments > 2 cM overlapping the variant, which resided in a European haplotype as well (Additional file 1: Figure S30). To determine IBD at a smaller scale, we did a manual alignment of all the variant-carrying haplotypes and detected an IBD segment of ~ 1 cM between all the TANGL cohort and 1000GP European *SQSTM1* c.1175C>T (p.Pro392Leu) carriers (Additional file 1: Figure S31). This observation suggests that *SQSTM1* c.1175C>T (p.Pro392Leu) shows the signature of a founder effect that pre-dates the Spanish invasion. Variants with higher allelic frequency also show IBD between the TANGL cohort and with other carriers outside of Colombia.

In contrast to the pathogenic variants in the FTLD-MND associated genes, five of the eight disease associated variants identified in the ALS panel were of Native American origin while only two were of European ancestry (Table 2). However, most of these individuals with pathogenic and likely pathogenic variants in Native American haplotypes presented with FTLD phenotypes (Additional file 8: Table S6 and Additional file 9: Table S7). For example, the *TUBA4A* [MIM: 191110] c.820C>G (p.Pro274Ala) variant was identified in two independent families with positive family histories of dementia and diagnosis of bvFTD and EOD without motor neuron disease (Additional file 1: Figure S32). As described previously for other variants, these families shared a long IBD haplotype of 15.54 cM overlapping the locus, suggesting a recent common ancestor (Additional file 1: Figure S33). The *SOD1* [MIM: 147450] c.63C>G (p.Phe21Leu) variant was identified in one patient with sporadic navPPA who did not have any motor or ALS-associated symptoms. This variant and others in this same amino acid [c.62 T>G (p.Phe21Cys)] had been previously reported in patients with ALS [120, 121]. Additional likely pathogenic variants in *ANXA11* [MIM: 602572] and *HNRNPA2B1* [MIM: 600124] residing in Native American haplotypes were identified in patients with svPPA and bvFTD. These results further intertwine ALS and FTLD with several genes previously associated exclusively with ALS that may also be responsible for a FTLD phenotype in a different ancestral context. The genetic and clinical heterogeneity of ALS associated genes had been previously described in European population [122], but the inclusion of diverse individuals expands the extent of genetic overlap between FTLD and ALS.

A patient with PSP was homozygous by descent for a European haplotype harboring the *FIG4* [MIM: 609390], c.122 T>C (p.Ile41Thr). Although this gene has been associated with autosomal dominant forms of ALS, this same specific variant has been reported in compound heterozygosity with nonsense variants in European individuals with autosomal recessive cases of Charcot-Marie-Tooth’s disease [123] [MIM: 611228]. A family presenting with FTLD-ALS was shown to have a novel c.724G>A (p.Ala242Thr) variant in *UBQLN2* [MIM: 300264]. *UBQLN2*, found on the X-chromosome, is associated with ALS or FTLD-MND, with a lower penetrance in females [124]. The family with this mutation had late onset bvFTD presentation in the female carrier, while the male carrier had FTLD-MND (Additional file 1: Figure S34).

#### Other genes associated with neurodegenerative disorders

Several families with EOD were explained by variants in other non-AD-FTD-ALS genes (Additional file 1: Figures S14 and S15). A family with an unspecified autosomal dominant EOD had a novel mutation in *DNAJC5* [MIM: 611203] c.347 T>G (p.Leu116Arg) residing on an African haplotype. Their phenotype and postmortem brain tissue histopathology was compatible with adult-onset ceroid neuronal lipofuscinosis-4B (CNL4B) [MIM: 162350] (Additional file 1: Figure S35). A novel likely pathogenic variant in *LRRK2* [MIM: 609007] c.4334C>G (p.Ser1445Cys) was identified in a patient with a European background and non-motor symptoms in Parkinson’s disease and dementia. One patient with a family history of cancer and dementia carried the *CSF1R* [MIM: 164770] c.2068G>A (p.Gly690Ser) variant in a Native American haplotype. *CSF1R* mutations have been associated with Hereditary Diffuse Leukoencephalopathy with Spheroids (HDLS) [125] [MIM: 221820] A postmortem brain tissue examination supported HDLS diagnosis for the *CSF1R* c.2068G>A (p.Gly690Ser) variant carrier (Additional file 1: Figure S36). These families provide novel insights on genetic-phenotypic relationships.

Despite an extensive evaluation of known genes previously reported for Mendelian forms of dementia, we were not able to identify a disease-causing variant in all families with autosomal dominant inheritance of the illness. Of the 566 families included in the present study, 59 had autosomal dominant inheritance defined as three or more affected individuals in two consecutive generations (Additional file 11: Table S9). For the 18 families in which all individuals had early onset of symptoms (< 65 years), we could identify disease causing variants in all but three, and 13 of them carried pathogenic *PSEN1* variants. In families with both early and late onset cases, we identified disease causing variants in seven of 33. No disease-causing variant was identified in the 12 individuals from the eight families where everyone had late onset, but 10 of them carried at least one *APOE* [MIM: 107741] ϵ4 allele (two were *APOE* ϵ3/ϵ3, six were ϵ3/ϵ4, and four ϵ4/ϵ4). In conclusion, a pathogenic or likely pathogenic variant was identifiable in the families with autosomal dominant inheritance in which most of the affected individuals had disease onset before 65 years.

#### Genetic variation associated with AD risk genes

Both rare and common variants can have a small effect size on AD risk [126]. To explore rare variants conferring intermediate risk for the illness, we selected three genes (*TREM2*, *SORL1*, and *ABCA7*) that have shown odds ratio (OR) higher than two (OR > 2) in disease association studies [69] Using the criteria suggested by Bellenguez et al. [69], we identified 14 protein truncating variants (PTV) and 16 strictly damaging (SD) variants in *TREM2*, *SORL1*, and *ABCA7* (Table 3 and Additional file 12: Table S10).

**Table 3:** Variants in risk-associated genes

Gene	Coding change	dbSNP/ClinVar	Classification	ExAC	1000G	CADD	Local ancestry
***ABCA7***	c.2T>C	rs1347920426	PTV (nonsense)	.	.	24.9	Native American
	c.236A>C (p.Asn79Thr)	rs377401443	SD	4.16E-05	.	24.5	African
	c.1180_1190del (p.Leu396fs)	rs567222111	PTV (frameshift)	0.0005	0.0022	.	African
	c.1531G>T (p.Glu511*)	rs374932832	PTV (nonsense)	7.60E-05	.	39	African
	c.1776G>T (p.Trp592Cys)	SCV001751545	SD	.	.	26	African
	c.2124_2130del (p.Glu709fs)+	rs547447016	PTV (frameshift)	0.0024	0.0006	.	European
	c.2194C>T (p.Gln732*)	rs1030634619	PTV (nonsense)	.	.	36	European
	c.2552+11_2552+58del	rs1178315251	PTV (splice)	.	.	.	African
	c.2611G>C (p.Asp871His)	rs139251928	SD	0.0004	0.0014	24.8	African
	c.3781delC (p.Pro1261fs)	SCV001751546	PTV (frameshift)			.	Native American
	c.4208delT (p.Leu1403fs)	rs538591288	PTV (frameshift)	0.0011	.	.	European
	c.4465C>T (p.Arg1489*)	rs753664323	PTV (nonsense)	6.66E-05	.	39	European
	c.4886C>T (p.Ser1629Leu)+	rs184590335	SD	0.0012	0.0006	35	Native American
	c.4895C>T (p.Pro1632Leu)	rs143083561	SD	0.0002	0.0006	34	African
	c.5302delC (p.Leu1768fs)	rs1348650979	PTV (frameshift)	.	.	.	Native American
	c.5463+2T>C	rs374611445	PTV (splice)	2.81E-05	.	23.7	European
	c.5794C>T (p.Arg1932C)	rs114787084	SD	0.0002	0.0006	34	African
***SORL1***	c.994C>T (p.Arg332Trp)	rs772110877	SD	5.77E-05	.	35	European
	c.1432G>C (p.Ala478Pro)	SCV001751547	SD	.	.	28.2	European
	c.1496C>T (p.Ser499Leu)	rs764032259	SD	8.24E-06	.	35	European
	c.2200G>A (p.Asp734Asn)	rs148430425	SD	0.0011	.	34	European
	c.2230C>T (p.Arg744*)	rs1050845490	PTV (nonsense)	.	.	39	European
	c.2710C>T (p.Arg904Trp)	rs148966249	SD	4.12E-05	2.00E-04	33	Native American
	c.3679G>T (p.Gly1227Cys)	rs1765488318	SD	.	.	34	European
	c.4520C>T (p.Pro1507Leu)	rs1308522330	SD	.	.	26.2	Undetermined
	c.6550G>A (p.Ala2184Thr)	rs369618646	SD	4.16E-05	.	34	African
***TREM2***	c.140G>A (p.Arg47His)+	rs75932628	SD	0.0021	0.002	33	European
	c.469C>T (p.His157Tyr)+	rs2234255	SD	0.0036	0.0028	23.1	Native American
	NM_001271821 c.287C>A (p.Thr96Lys) c.572G>A(p.Trp191*) c.632T>C (p.Leu211Pro)	rs2234253 rs2234258 rs2234256	PTV (nonsense)				African
	c.594G>A (p.Trp198*)	rs1765488318	PTV (nonsense)	.	.	39	Undetermined
***ADAM10***	c.510G>C (p.Gln170His)+	rs61751103	SD	0.0012	0.0012	19.17	European

*PTV* protein truncating variant, *SD* strictly damaging, *ExAC* ExAC database minor allelic frequency. CADD corresponds to the Phred score. Variants denoted with a + were identified in homozygous states. GenBank transcripts for each gene can be found in Additional file 4: Table S3

The most common risk-conferring variants in the TANGL cohort resided on *TREM2*, with over a hundred individuals carrying SD or PVT in this gene (Additional file 12: Table S10). The most prevalent variant was c.469C>T (p.His157Tyr), with 50 heterozygous and seven homozygous carriers. All the c.469C>T (p.His157Tyr) carriers were IBD for a Native American haplotype. Two out of three algorithms classified His157Tyr as definitely pathogenic, while a meta-analysis determined *TREM2* c.469C>T (p.His157Tyr) has an OR = 3.65 [127], and therefore, it qualified for the present study. Additionally, we identified 33 *TREM2* c.140G>A (p.Arg47His) carriers in our cohort; three of them were homozygous for this variant (Additional file 12: Table S10). All the *TREM2* c.140G>A (p.Arg47His) carriers from the TANGL cohort shared an IBD European haplotype overlapping the *TREM2* locus, and this same variant-carrying haplotype was present in five European individuals from the 1000GP who showed IBD with the Colombian carriers (Additional file 1: Figure S37). Besides risk conferring variants in Native American and European haplotypes, an African *TREM2* haplotype [GenBank: NM_001271821] carrying c.572G>A (p.Trp191*), c.632 T>C (p.Leu211Pro), and c.287C>A (p.Thr96Lys) was identified in 10 individuals. This haplotype was previously associated with an increased risk in African-American cohorts [128]. Unlike the previous cases of homozygosity, one individual with early-onset AD was a compound heterozygote with both the Thr96Lys/Trp191*/Leu211Pro haplotype and the c.469C>T (p.His157Tyr) variant, suggesting that genetic risk factors from different ancestral origins may coexist in admixed individuals and populations.

Rare variants in *TREM2* are population specific. For example, *TREM2* c.140G>A (p.Arg47His) is associated with increased risk for AD in European descent populations [129, 130] but not in African [128] or Asian [131, 132], while *TREM2* c.469C>T (p.His157Tyr) shows association with AD in Asian [127, 133] but not in European [134] or African [128] cohorts. Interestingly, the c.469C>T (p.His157Tyr) variant was found in Colombia on a Native American haplotype, raising the possibility that this allele arrived from Asia to the American continent close to the time when the Americas were first populated 15,000–20,000 years ago. To support this hypothesis, we searched for this variant in the Human Genome Dating database [135], which uses coalescent modeling to estimate the time to the most recent common ancestor (TMRCA) between the variant carriers and the age of the variant. The estimated age of the c.469C>T (p.His157Tyr) allele is 1265 generations (95% confidence interval of 1108.5–1430.9), which corresponds to 31,625 years by setting one generation equivalent to 25 years (https://human.genome.dating/snp/rs2234255). In contrast, the c.140G>A (p.Arg47His) variant emerged more recently, as it was estimated to be 425 generations old or 10,625 years (https://human.genome.dating/snp/rs75932628), dating to a time before gene flow from Europe to the Americas occurred. These results lead us to conclude that the disease burden in this population is not only affected by the recent admixture after the conquest of the Americas, but was also affected by migrations [136] during the original populating of the continent.

Risk-conferring variants in *ABCA7* and *SORL1* were less prevalent than those in *TREM2*. Most of the variants detected in *ABCA7* consisted in PTV and resided on African haplotypes (Additional file 1: Figure S37). The majority in *SORL1* were SD variants of European origin, two homozygous carriers of *ABCA7* variants c.2124_2130del (p.Glu709fs) and c.4886C>T (p.Ser1629Leu), and a compound heterozygote of risk variants from different ancestral origins. There were no compound heterozygous or homozygous variants for *SORL1*, and the c.6550G>A (p.Ala2184Thr) variant was only found in a healthy centenarian. Additionally, a search for risk associated variants in *ADAM10* [77, 78], identified c.510G>C (p.Gln170His) in ten individuals, including one homozygous patient. These reported variants in *TREM2*, *SORL1*, *ABCA7*, and *ADAM10* were IBD in carriers of the same variant (Additional file 1: Figures S37, S38, S39 and S40). In summary, the characteristics we described for disease-causing variants such as IBD between carriers, multiple ancestral origins of deleterious variants within the same gene, and autozygosity were present in variants with higher allelic frequencies in risk-associated genes.

The high allelic frequency of some risk conferring variants in the TANGL cohort allowed the detection of individuals who were homozygous by descent and raised the hypothesis of consanguinity between their parents, as was the case for the two families with recessive dementias [*TBK1* c.1717C>T (p.Arg573Cys) and *FIG4* c.122 T>C (p.Ile41Thr)]. We used Hap-IBD and manual haplotype alignment to estimate the autozygosity of the homozygous individual for risk-associated variants in *ABCA7* [c.2124_2130del (p.Glu709fs) and c.4886C>T (p.Ser1629Leu)], *TREM2* [c.140G>A (p.Arg47His) and c.469C>T (p.His157Tyr)] and *ADAM10* [c.510G>C (p.Gln170His)]. Five individuals from three families who were the offspring of related parent had autozygous segments > 30 cM overlapping the risk associated variant (Additional file 13: Table S11). The remaining individuals had smaller autozygous segments, suggesting background relatedness of the population due to a small effective population size or bottlenecks [137, 138].

## Discussion

Genetic drift has been one of the main forces shaping human genomic variation [139, 140]. While populations that emerge from a bottleneck will harbor reduced genetic variation, over time, such a population can accumulate higher numbers of deleterious variants due to random fluctuations in allele frequencies [141]. Furthermore, deleterious allele frequencies decrease more slowly in smaller populations because natural selection acts on fitness differences and therefore requires genetic variation [141]. The Colombian tri-continental admixture among the Native Americans, Europeans, and Africans combined a portion of the genetic disease burden that was previously limited to each of these ancestral populations. Within the backdrop of an admixed population, numerous infectious diseases extracted a very steep mortality. As a consequence, the small isolated settlements that survived the bottleneck rapidly expanded locally during the colonial period [1]. These multiple isolated bottlenecks each with their own rare variants added to the diversity over the entire population. The TANGL cohort recapitulated the admixture patterns previously described in the Colombian population, suggesting that the country´s demographic history is likely to underlie the modern clustering of familial neurodegenerative diseases arising from multi-ancestral rare disease-associated alleles.

In this cohort, most familial early-onset AD cases were caused by variation in the *PSEN1* gene. We identified eleven different pathogenic *PSEN1* variants from multiple ancestral origins, nearly all attributed to founder effects. The *PSEN1* mutations emerged from a small effective population in each of the early settlements that constituted a patchwork of bottlenecks dispersed throughout the country. Because people tended to remain geographically isolated, the rare variants represent a local genetic footprint. Survivors who emerged from the bottleneck had escaped the large number of infectious diseases responsible for decimating the population. During the historical period of colonization, populations in these settlements grew rapidly as the incidence of diseases diminished, which favored the segregation of potentially damaging variants at higher rates. The question arises as to whether the *PSEN1* mutations could be under positive selection or are the mutations completely explained by drift. Because *PSEN1* mutant phenotypes do not appear until after the age of child-bearing, it is unnecessary to invoke trade-off effects for maintaining the mutation in the population. Positive selection for Alzheimer risk in the context of infectious burden has been previously attributed to the *APOE* ϵ4 risk allele [142]. *PSEN1* mutations cause the production of excess amyloid-beta, which may function as an anti-microbial peptide (AMP) [143]. In this manner, *PSEN1* mutations may have been positively selected as protection against the enormous mortality of infectious diseases. AMPs function as an ancient component of the innate immune system that target bacteria, mycobacteria, enveloped viruses, fungi, and protozoans [144]. Amyloid beta is active against at least eight common and clinically relevant microorganisms, and several anti-amyloid-beta clinical trials have reported increased rate of infections among the participants [143, 145]. However, given the short ~ 500-year interval since the selective pressure occurred and the ~ 100-year pulse-like nature of the selection, the possibility of positive selection must remain speculative. Without a sufficient time interval for the mutation to spread widely through the population, the only indirect support for positive selection might consider the collective fitness conferred by all of the *PSEN1* mutations due to their shared phenotypic effect of increasing amyloid beta as an AMP. Whether these mutations represent a statistical excess will require further study, but given the population size at the time to which the mutations can be historically traced (see ancestry data for each mutation), it is likely that the mutations derived from a small effective population, thus supporting their possible over-representation. A comparison comes from large catchment groups for clinics with an interest in familial dementias—one in Alabama had no *PSEN1* cases in their series [146] and another in San Francisco had six *PSEN1* cases (personal communication, Jennifer Yokoyama, University of California San Francisco). In one study that sought early-onset Alzheimer patients from 28 university hospitals across France spanning the dates 1993 to 2016, 17 sporadic cases carried a *PSEN1* mutation [104]. However, any comparison with our cases is problematic because ten of these arose de novo, which was not the case in the TANGL cohort, and some were of unknown pathogenicity.

In addition to the *PSEN1* variants, we identified multiple rare variants causing autosomal dominant early-onset dementia. Variants were usually found in one locality and likely derived from a common ancestor (Additional file 1: Figure S41). Previous studies had reported disease causing variants for other neurological disorders with the signature of founder effects; among these are four different cerebral autosomal dominant arteriopathy with subcortical infarcts and leukoencephalopathy (CADASIL) [MIM: 125310] associated variants in *NOTCH3* [MIM: 600276, c.307C>T(p.Arg103Cys), c.421C>T (p.Arg141Cys), c.484 T>A (p.Cys162Ser), c.1363 T>C (p.Cys455Arg)] [147, 148], a familial episodic pain syndrome [MIM: 615040] with a variant in *TRPA1* [MIM: 604775, c.2564A>G (p.Asn855Ser)] [149], Huntington’s disease [150] [MIM: 143100], a Parkinson disease variant in *LRRK2* [c.6055G>A (p.Gly2019Ser)] [151], blepharophimosis-ptosis-epicanthus inversus syndrome (BPES) [MIM: 110100] type 1 with a *FOXL2* [MIM: 605597, c.157C>T (p.Gln53*)] variant and BPES type 2 with *FOXL2* in-frame 30 bp duplication (c. 909–938dup) [152], a complex ataxia due to a *KIF1A* variant [MIM: 601255, variant c.304G>C (p.Gly102Arg)], generalized epilepsy with febrile seizures plus (GEFS+) [MIM: 604403] with *SCN1A* [MIM: 182389 c.5225A>G (p.Asp1742Gly)] variant [153], and non-syndromic hearing loss [MIM: 220290] due to a *GJB2* variant [MIM: 121011 c.35delG (p.Gly12Valfs∗] [154] . Founder effects can also be detected in other non-neurologic conditions: *BRCA1/2* variants [MIM: 113705, 600185] among Colombian women with breast and ovary cancer increased the prevalence of these variants in the studied population [155]. Most of these mutations map to small distinct locales that when, taken together, demonstrate the remarkable overlap of the genetic and geographic maps.

This study underscores the numerous genetic insights that can emerge from Latin American populations. Another example is the putative modifier gene—homozygosity of the Christchurch variant in ApoE3—that may strongly delay the onset of Alzheimer’s disease [156]. This gene variant and many of the rare large effect size mutations reported here arose due to the unique genetic history of the region. Ongoing interest in Latin American genetic studies, akin to all genetic studies in under-represented populations, must consider the ethical implications of the research. Over the many years these were obtained, the research was conducted with the full involvement of the community and extensive interactions with and informed consent from the contributing families.

## Conclusions

Demographic history plays a significant role in shaping a population’s genetic risk for disease. The genetic complexity of the dementias offers a phenotypic heading for a search to uncover genetic variation for the familial dementias. In the Colombian population, founder effects led to a large number of ancestral disease-causing alleles from each of three admixed continents. We also observed a confluence of rare variants arising from different ancestral origins in dementia risk-conferring genes. Variants of different ancestries combined to create a heterogeneous landscape for the genetic risk of dementia. In addition to the significant role of admixture and drift, we raise the question of whether positive selection of *PSEN1* mutations could contribute to the large number of these in a relatively small effective population size. *PSEN1* variants lead to excess of amyloid-beta, which may function as anti-microbial protein and may have protected against the massive mortality due to infectious diseases during the conquest and colonization of the Americas. This work reinforces the need to include diverse populations for gene-trait association studies including populations that underwent bottlenecks as a source for gene discovery.

## Supplementary Information

**Additional file 1: Figure S1.** Demographic information of the TANGL cohort and the Colombian population. **Figure S2.** Pipeline for whole genome sequence data quality control (QC). **Figure S3.** Principal Component Analysis of whole genomes from 1000 Genomes project and the TANGL cohort. **Figure S4.** Cross validation error for unsupervised ADMIXTURE clustering analysis of the TANGL cohort probands. **Figure S5.** Cross Validation Error for unsupervised ADMIXTURE clustering of the multi-ancestral dataset (TANGL genomes with the European and African populations from the 1000GP and Native American genomes from Mao et al. **Figure S6.** Global ancestry proportions of the TANGL cohort calculated by ADMIXTURE and sum of RFMix local ancestry estimation. **Figure S7.** Correlation of global ancestry proportions calculated for each individual by two different software, RFMix sum of local ancestries vs ADMIXTURE. **Figure S8.** Principal component analyses of the African and European cohorts of the 1000GP, along with 43 Native American genomes and the TANGL cohort. **Figure S9.** Principal component analyses of the African and European cohorts of the 1000GP, along with 43 Native American genomes and the TANGL cohort colored according to their proportions of global ancestry. **Figure S10.** Correlation of the principal component 1 and 2 values and the global ancestry proportions. For the TANGL.AFR.EUR.NAT cohort. **Figure S11.** Principal component analyses of the TANGL cohort colored according to their proportions of global ancestry. **Figure S12.** Correlation of the principal component 1 and 2 values and the global ancestry proportions for the TANGL cohort using common variants (MAF >10%). **Figure S13.** Correlation of the principal component 1 and 2 values and the global ancestry proportions for the TANGL cohort using common variants (MAF 5-10%). **Figure S14**. Pipeline of the curation of disease-causing variants in the TANGL cohort. **Figure S15.** Variant filtering of disease-causing variants in the TANGL cohort. **Figure S16.** Pedigrees of the families with pathogenic variants in *PSEN1* (NM_000021). **Figure S17.** Pairwise identity by Descent (IBD) segments in the chromosomes that harbor the *PSEN1* NM_000021 c.791C>T (p. Pro264Leu) variant. **Figure S18.** Pairwise identity by Descent (IBD) segments in the chromosomes that harbor the *PSEN1* NM_000021 c.428T>C (p.Ile143Thr) variant. **Figure S19.** Pairwise identity by Descent (IBD) segments in the chromosomes that harbor the *PSEN1* NM_000021 c.356C>T (p.Thr119Ile) variant in Colombian individuals. **Figure S20.** Pairwise identity by Descent (IBD) segments carrying the *PSEN1* NM_000021 c.356C>T (p.Thr119Ile) variant in Colombian and Argentinian individuals. **Figure S21.** Pedigrees of the family with a pathogenic variant in *PSEN2* (NM_000447). **Figure S22.** Depth and allele balance indicate a duplication including *APP*. **Figure S23.** Pedigrees of the families with pathogenic variants in *MAPT* (NM_005910). **Figure S24.** Pairwise identity by Descent (IBD) segments in the chromosomes that harbor the *MAPT* NM_005910 c.1189C>T (p.Pro397Ser) variant. **Figure S25.** Pairwise identity by Descent (IBD) segments in the chromosomes that harbor the *MAPT* NM_005910 c.1189C>T (p.Pro397Ser) variant from Colombian and Spanish families. **Figure S26.** Pedigrees of the families with pathogenic variants in *TBK1* (NM_013254). **Figure S27.** Pairwise identity by Descent (IBD) segments in the chromosomes that harbor *TBK1* NM_013254 c.1257_1258del (p.Val421Cfs) variant. **Figure S28.** Pedigree of the family with a pathogenic variant in *TARDBP* (NM_007375). **Figure S29.** Pedigree of the family with a pathogenic variant in *GRN* (NM_002087). **Figure S30.** Pairwise identity by Descent (IBD) segments in the chromosomes that harbor *SQSTM1* NM_003900 c.1175C>T (p.Pro392Leu) variant in the TANGL cohort. **Figure S31.** Alignment of the haplotypes that harbor *SQSTM1* NM_003900 c.1175C>T (p.Pro392Leu) variant in the TANGL and the 1000GP cohort. **Figure S32.** Pedigrees of the families with pathogenic variants in *TUBA4A* (NM_006000). **Figure S33**. Pairwise identity by Descent (IBD) segments in the chromosomes that harbor *TUBA4A* NM_006000 c.820C>G (p.Pro274Ala) variant. **Figure S34.** Pedigrees of the families with pathogenic variants in *UBQLN2* (NM_0013444) identified by the present study. **Figure S35.** Histological characterization of ceroid neuronal lipofuscinosis-4B (CNL4B) and Pedigree of the family. **Figure S36.** Histological characterization of hereditary diffuse leukoencephalopathy with spheroids (HDLS). Bottom row and  Pedigree of the family. **Figure S37.** Alignment of the haplotypes that carry Strictly Damaging and Protein Truncating Variants in *TREM2* present in more than 1 individual. **Figure S38.** Alignment of the haplotypes that carry Strictly Damaging and Protein Truncating Variants in *ABCA7* present in more than 1 individual. **Figure S39.** Alignment of the haplotypes that carry Strictly Damaging and Protein Truncating Variants in *SORL1* present in more than 1 individual. **Figure S40.** Alignment of the haplotypes that carry Strictly Damaging and Protein Truncating Variants in *ADAM10* present in more than 1 individual. **Figure S41.** Maps of Colombia representing the place of origin of the families with disease causing variants.

**Additional file 2: Table S1.** Demographic information of the included cohorts and their respective sub-cohorts.

**Additional file 3: Table S2.** Demographic information of the probands from included cohorts and their respective sub-cohorts.

**Additional file 4: Table S3.** GenBank accession numbers for the genes reported in the present study.

**Additional file 5: Table S4.** Mitochondrial haplogroups of the probands.

**Additional file 6: Table S5.** Y chromosome haplogroups of the male probands.

**Additional file 7:.** Supplementary methods.

**Additional file 8: Table S6.** Pathogenic variants identified in disease causing genes with additional information of the carriers.

**Additional file 9: Table S7.** Phenotypic information of the carriers of pathogenic variants in disease causing genes.

**Additional file 10: Table S8.** Neuropsychological battery performance in *MAPT* c.1189C>T (p.Pro397Ser) carriers vs non-carriers according to their age groups and clinical diagnosis.

**Additional file 11: Table S9.** Family history of dementia and or motor neuron disease from the 566 probands.

**Additional file 12: Table S10.** Additional information of the carriers of Protein Truncating Variants (PVT) and Strictly Damaging variants (SD) in risk conferring genes.

**Additional file 13: Table S11.** Homozygosity by descent (HBD) in carriers of disease causing and risk conferring variants.

## Data Availability

The genetic data obtained from the TANGL cohort (Raw data and BAM and VCF files aligned to hg19) have been deposited in the Grupo de Neurociencias de Antioquia (GNA) genetic data repository, Institutional repository of the Universidad de Antioquia (doi:10.5062/F4N58JNW) [157]. The Institutional Review Board (IRB) of the Medical Research Institute at the School of Medicine Universidad de Antioquia has restricted the deposition of the TANGL dataset to an institutional repository within the University of Antioquia. The TANGL dataset can be accessed and used by qualified researchers in collaborative projects involving the GNA. The application form for data access can be downloaded from the DOI link and should be emailed to juliana.acosta@gna.org.co. Applications are evaluated by GNA Neurogenetics Data Access Committee and response if given within 15 calendar days from application reception date. Novel “disease causing” and “risk conferring” variants that were not present in dbSNP and/or ClinVar databases were submitted to the National Center for Biotechnology Information ClinVar database [56]; https://www.ncbi.nlm.nih.gov/clinvar/ (accession numbers SCV001751539, SCV001751540, SCV001751542, SCV001751543, SCV001751544, SCV001751545, SCV001751546, SCV001751547, SCV001751549). The code used for the data analyses and plotting can be found at: https://github.com/acostauribe/TANGL (doi:10.5281/zenodo.5809622) [81].

## Abbreviations

1000GP: 1000 Genomes Project
ACMG: American College of Medical Genetics
AD: Alzheimer’s disease
AFR: African
ALS: Amyotrophic lateral sclerosis
AMP: Anti-microbial peptide
BPES: Blepharophimosis-ptosis-epicanthus inversus syndrome
bvFTD: Behavioral variant of frontotemporal dementia
CADASIL: Cerebral autosomal dominant arteriopathy with subcortical infarcts and leukoencephalopathy
CBD: Cortico-basal degeneration
CDR: Clinical Dementia Rating
CLM: Colombians from Medellín
CNL4B: Ceroid neuronal lipofuscinosis-4B
CNV: Copy number variation
CV: Cross validation
DNA: Deoxyribonucleic acid
EOD: Early-onset dementia not otherwise specified
EUR: European
FTLD: Frontotemporal lobar degeneration
GEFS+: Generalized epilepsy with febrile seizures plus
GWAS: Genome wide association studies
HDLS: Hereditary diffuse leukoencephalopathy with spheroids
IBD: Identity by descent
LD: Linkage disequilibrium
lvPPA: Logopenic variant of primary progressive aphasia
MAF: Minor allele frequency
MND: Motor neuron disease
NAT: Native American
navPPA: Non-fluent/agrammatic variant of primary progressive aphasia
OMIM: Online Mendelian Inheritance in Men database
OR: Odds ratio
PC: Principal component
PCA: Principal component analysis
PSP: Progressive supranuclear palsy
PTV: Protein truncating variants
SD: Strictly damaging
SNVs: Single nucleotide variants
SP: Spastic paraparesis
svPPA: Semantic variant of primary progressive aphasia
TANGL: The Admixture and Neurodegeneration Genomic Landscape
TMRCA: Time to the most recent common ancestor
WGS: Whole genome sequencing
