# Supplementary material for: A neurodegenerative disease landscape of rare mutations in Colombia due to founder effects
Source: Genome Med. 2022 Mar 8;14:27. doi: 10.1186/s13073-022-01035-9 (PMC8902761; doi:10.1186/s13073-022-01035-9)
Supplement: Supplementary file 1 — Additional file 1: Figure S1. Demographic information of the TANGL cohort and the Colombian population. Figure S2. Pipeline for whole genome sequence data quality control (QC). Figure S3. Principal Component Analysis of whole genomes from 1000 Genomes project and the TANGL cohort. Figure S4. Cross validation error for unsupervised ADMIXTURE clustering analysis of the TANGL cohort probands. Figure S5. Cross Validation Error for unsupervised ADMIXTURE clustering of the multi-ancestral dataset (TANGL genomes with the European and African populations from the 1000GP and Native American genomes from Mao et al. Figure S6. Global ancestry proportions of the TANGL cohort calculated by ADMIXTURE and sum of RFMix local ancestry estimation. Figure S7. Correlation of global ancestry proportions calculated for each individual by two different software, RFMix sum of local ancestries vs ADMIXTURE. Figure S8. Principal component analyses of the African and European cohorts of the 1000GP, along with 43 Native American genomes and the TANGL cohort. Figure S9. Principal component analyses of the African and European cohorts of the 1000GP, along with 43 Native American genomes and the TANGL cohort colored according to their proportions of global ancestry. Figure S10. Correlation of the principal component 1 and 2 values and the global ancestry proportions. For the TANGL.AFR.EUR.NAT cohort. Figure S11. Principal component analyses of the TANGL cohort colored according to their proportions of global ancestry. Figure S12. Correlation of the principal component 1 and 2 values and the global ancestry proportions for the TANGL cohort using common variants (MAF >10%). Figure S13. Correlation of the principal component 1 and 2 values and the global ancestry proportions for the TANGL cohort using common variants (MAF 5-10%). Figure S14. Pipeline of the curation of disease-causing variants in the TANGL cohort. Figure S15. Variant filtering of disease-causing variants in the TANGL cohort. Figure [file 13073_2022_1035_MOESM1_ESM.pdf]

ADDITIONAL FILE 1
SUPPLEMENTARY FIGURES

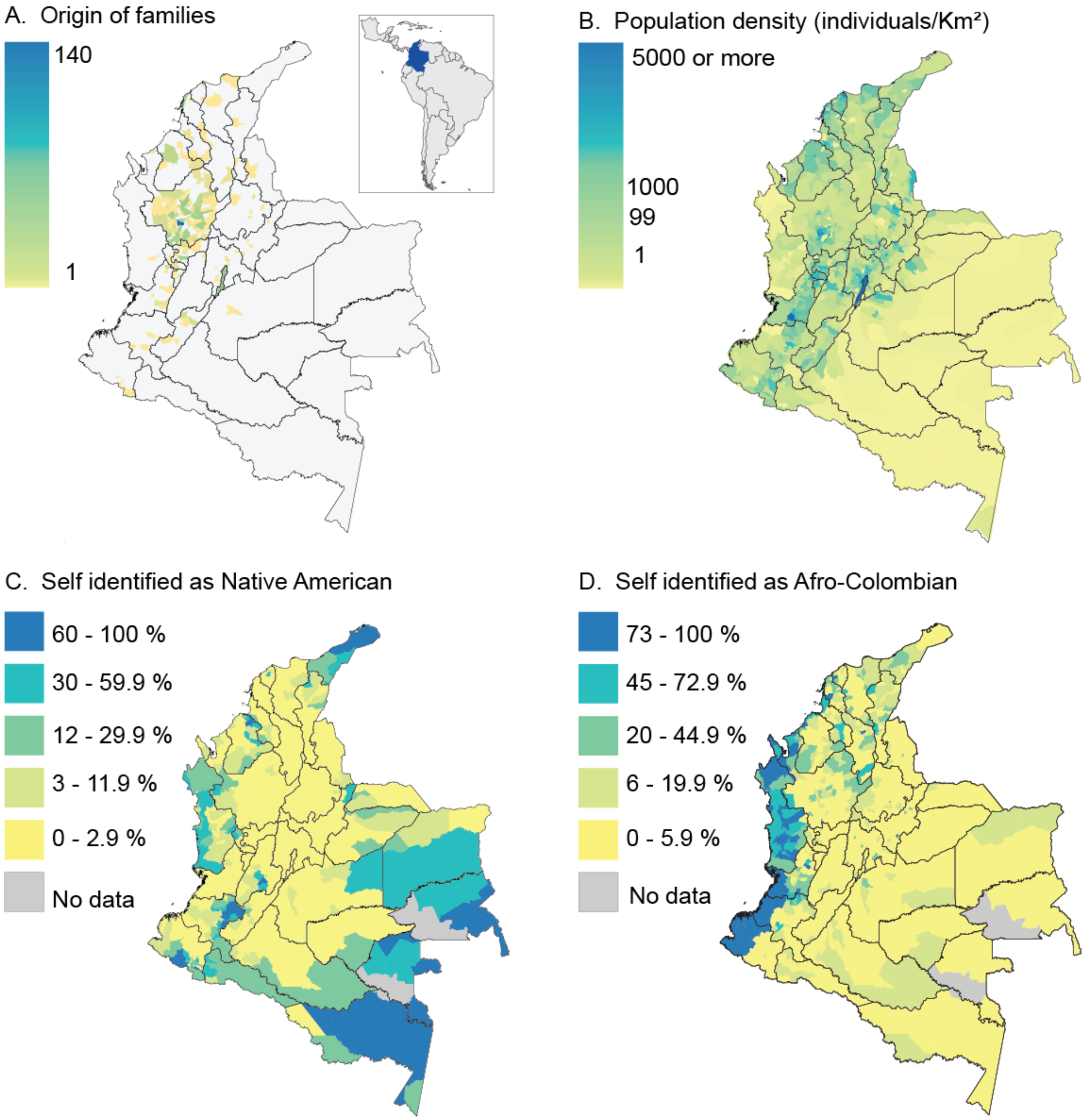

Figure S1.
Demographic information of the TANGL cohort and the Colombian population:
(A) Place of origin of the included families in the map of Colombia. **(B)** Population density in Colombia according to the 2018 census **(C and D)** Colombian population that self identifies as Native American or Afro-Colombian according to the 2005 census. Census data from 2018 and 2005 is publicly available by the "Departamento Administrativo Nacional de Estadística DANE": www.dane.gov.co

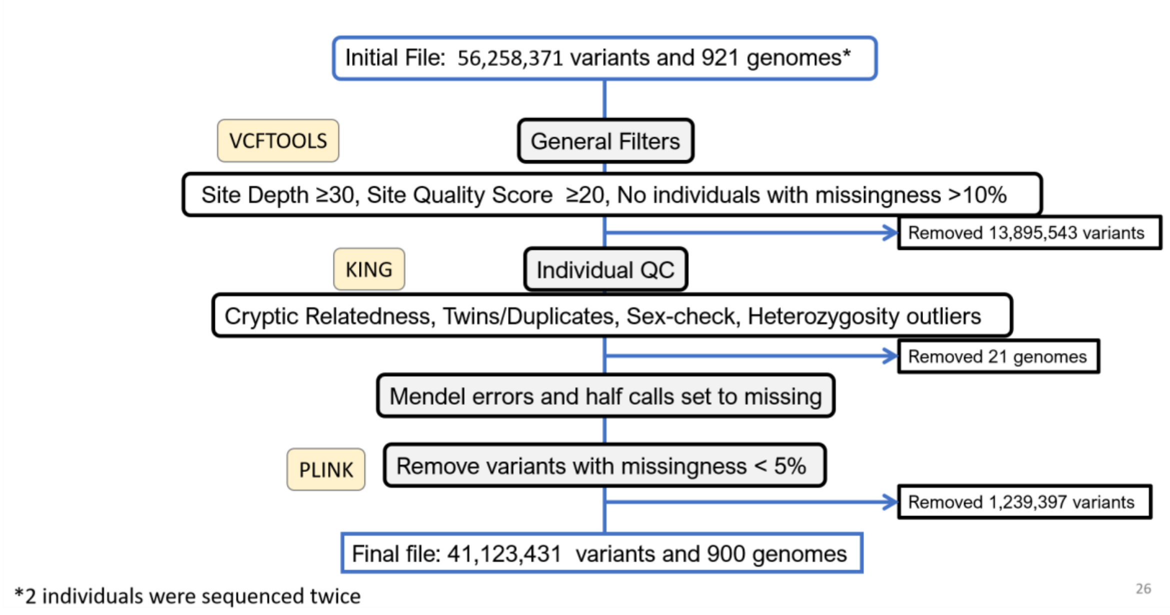

Figure S2:
Pipeline for whole genome sequence data quality control (QC)

The initial cohort included 919 individuals, but only 900 high quality genomes from 900 individuals were used for analyses in this project.

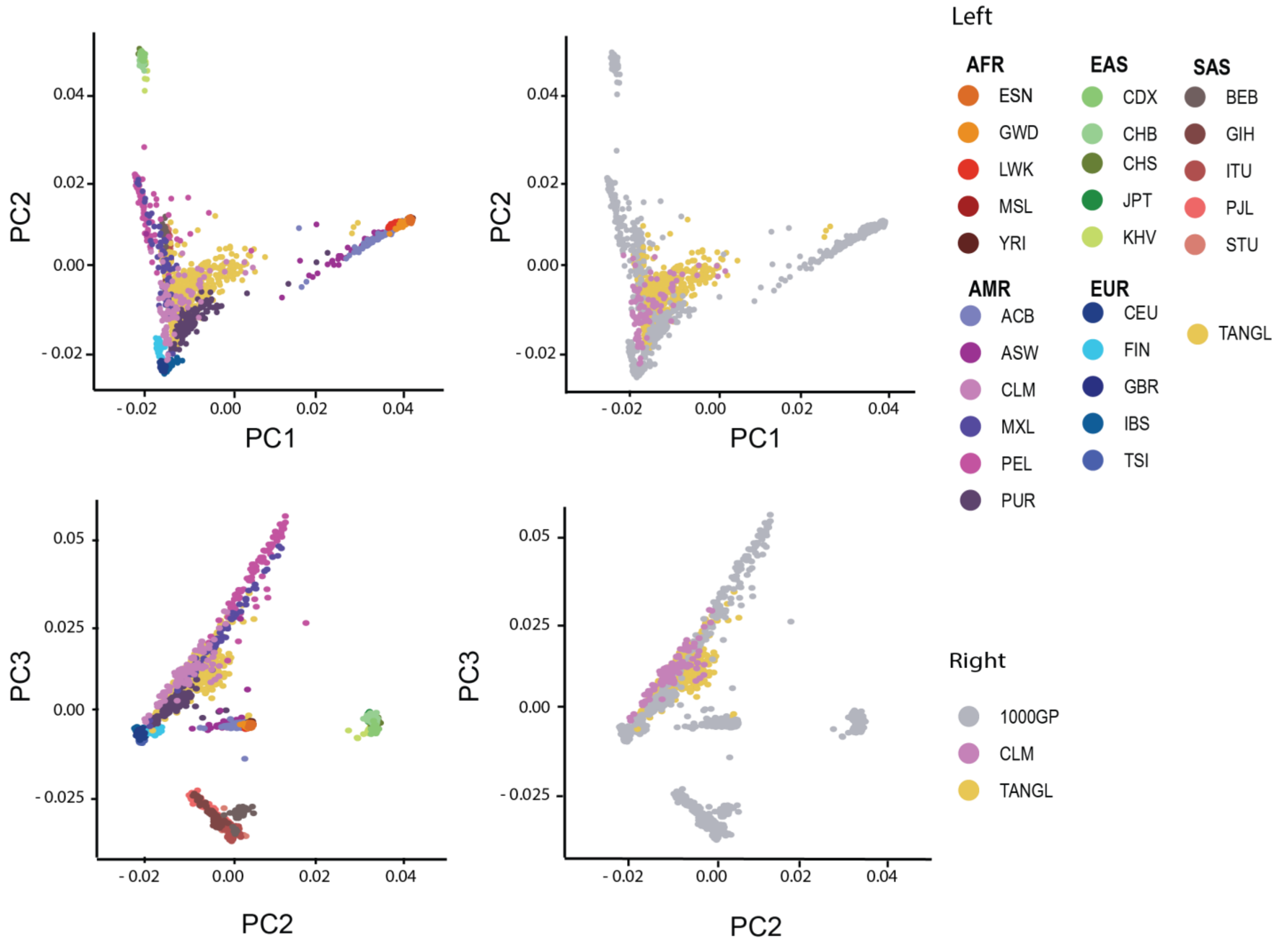

Figure S3.
Principal Component Analysis of whole genomes from 1000 Genomes project and the TANGL cohort.
ESN: Esan in Nigeria. GWD: Gambian in Western Divisions in the Gambia. LWK: Luhya in Webuye, Kenya. MSL: Mende in Sierra Leone. YRI: Yoruba in Ibadan, Nigeria. ACB: African Caribbean in Barbados. ASW: Americans of African Ancestry SW USA. CLM: Colombians from Medellin, Colombia. MXL: Mexican Ancestry from Los Angeles USA. PEL: Peruvians from Lima, Peru. PUR: Puerto Ricans from Puerto Rico. CDX: Chinese Dai in Xishuangbanna, China. CHB: Han Chinese in Beijing, China. CHS: Southern Han Chinese. JPT: Japanese in Tokyo, Japan. KHV: Kinh in Ho Chi Minh City, Vietnam. CEU: Utah Residents with Northern and Western European Ancestry. FIN: Finnish in Finland. GBR: British in England and Scotland. IBS: Iberian Population in Spain. TSI: Toscani in Italia. BEB: Bengali from Bangladesh. GIH: Gujarati Indian from Houston, Texas. ITU: Indian Telugu from the UK. PJL: Punjabi from Lahore, Pakistan. STU: Sri Lankan Tamil from the UK

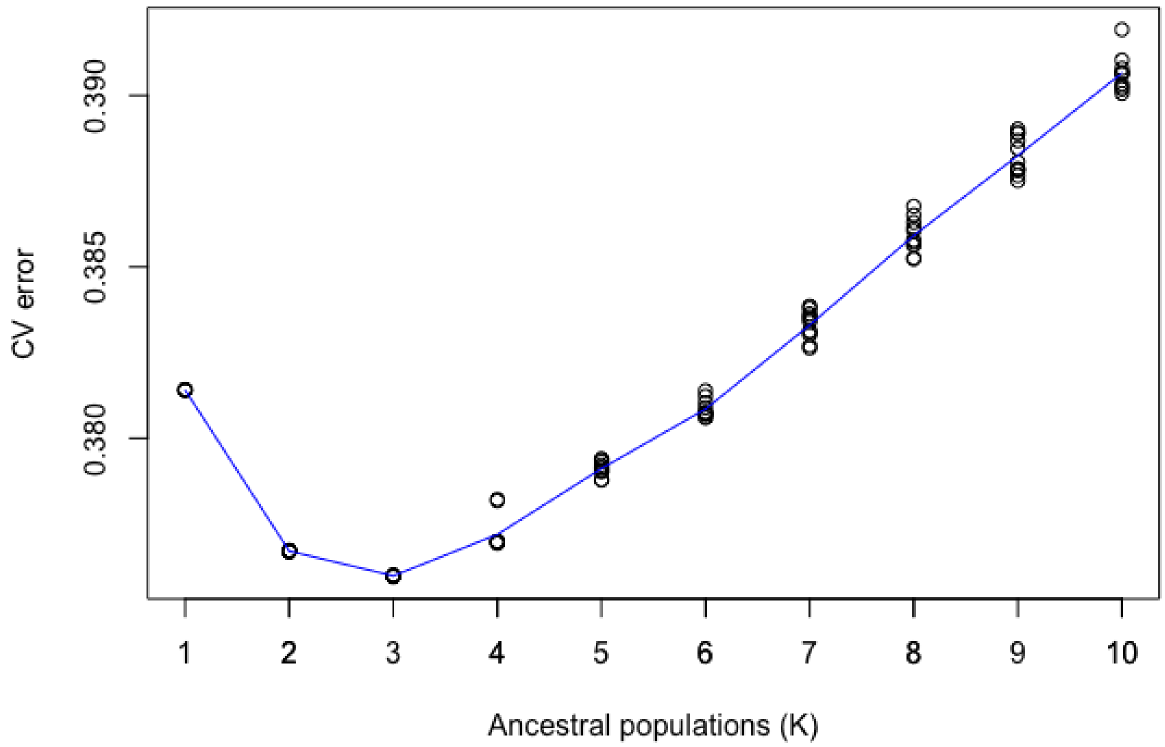

Figure S4.
Cross validation error for unsupervised ADMIXTURE clustering analysis of the TANGL cohort probands.
Y-axis represents the Cross-validation error calculated by each iteration of ADMIXTURE. Minimum cross validation error was identified at K = 3.

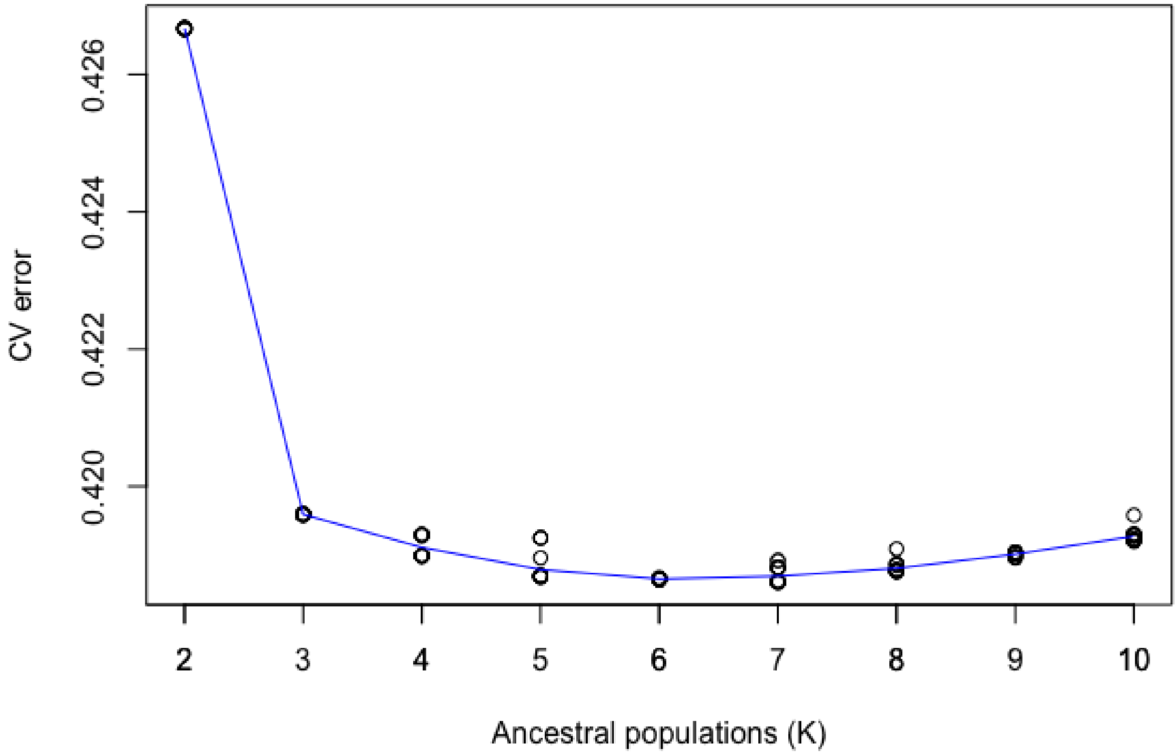

Figure S5.
Cross Validation Error for unsupervised ADMIXTURE clustering of the multi-ancestral dataset (TANGL genomes with the European and African populations from the 1000GP and Native American genomes from Mao et al[34]).
Y axis represents the Cross-validation error calculated by each iteration of ADMIXTURE. Minimum cross validation error was identified at K = 6.

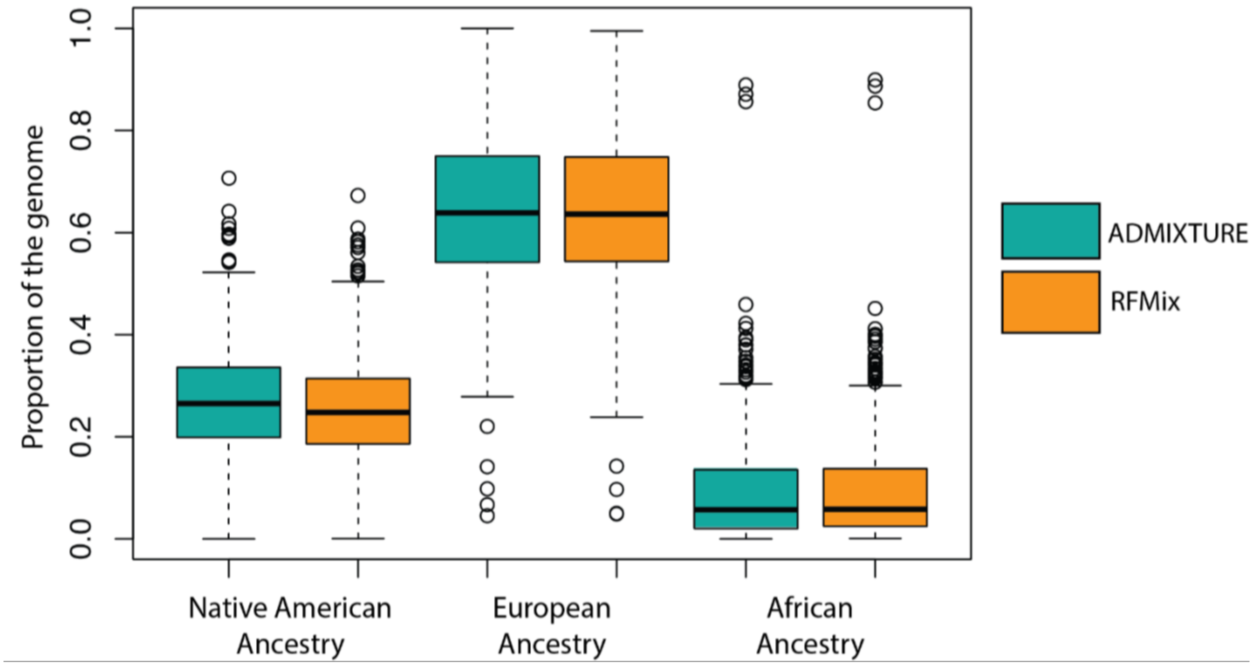

Figure S6.
Global ancestry proportions of the TANGL cohort calculated by ADMIXTURE and sum of RFMix local ancestry estimation.

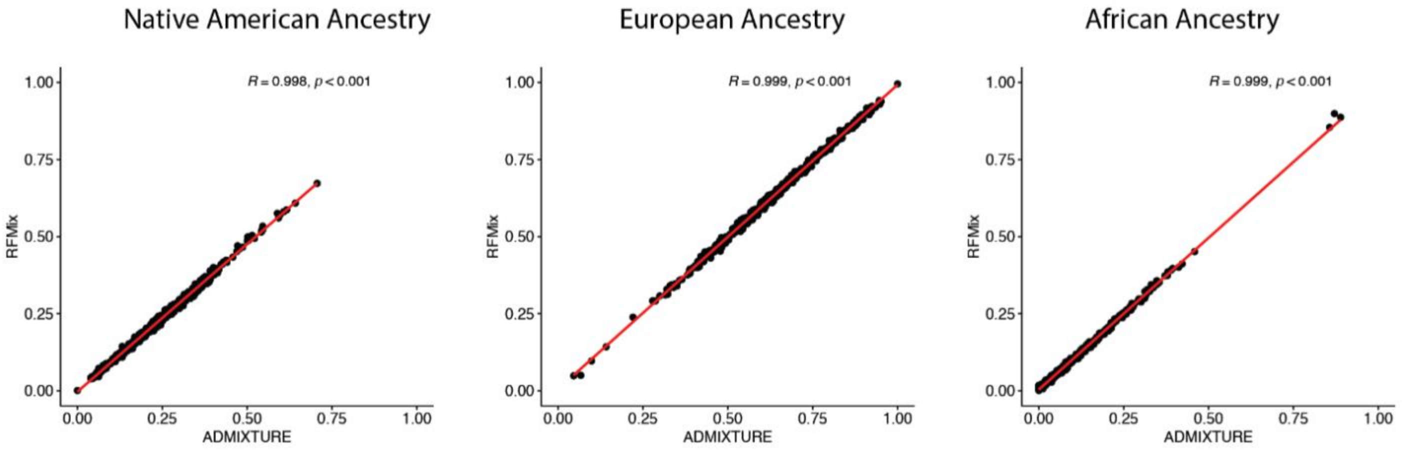

Figure S7
Correlation of global ancestry proportions calculated for each individual by two different software, RFMix sum of local ancestries (Y axis) vs ADMIXTURE (X axis).
R: Pearson correlation coefficient

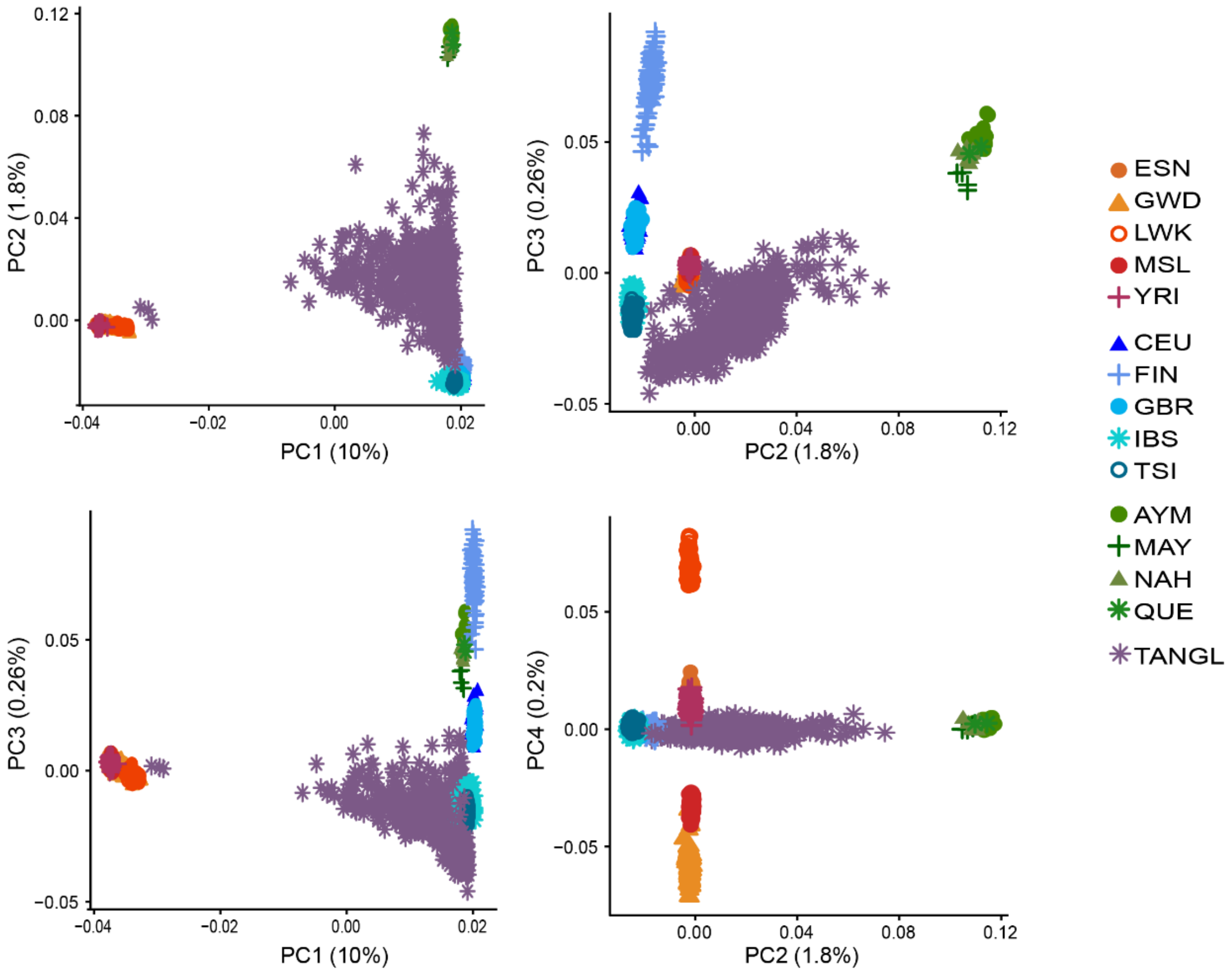

Figure S8

Principal component analyses of the African and European cohorts of the 1000GP, along with 43 Native American genomes and the TANGL cohort.

African are displayed in orange, European in blue, native American in Green and TANGL in purple. ESN: Esan in Nigeria. GWD: Gambian in Western Divisions in the Gambia. LWK: Luhya in Webuye, Kenya. MSL: Mende in Sierra Leone. YRI: Yoruba in Ibadan, Nigeria. CEU: Utah Residents (CEPH) with Northern and Western European Ancestry. FIN: Finnish in Finland. GBR: British in England and Scotland. IBS: Iberian Population in Spain. TSI: Toscani in Italia. AYM: Aymara. MAY: Mayan, NAH: Náhuatl. QUE: Quechua.

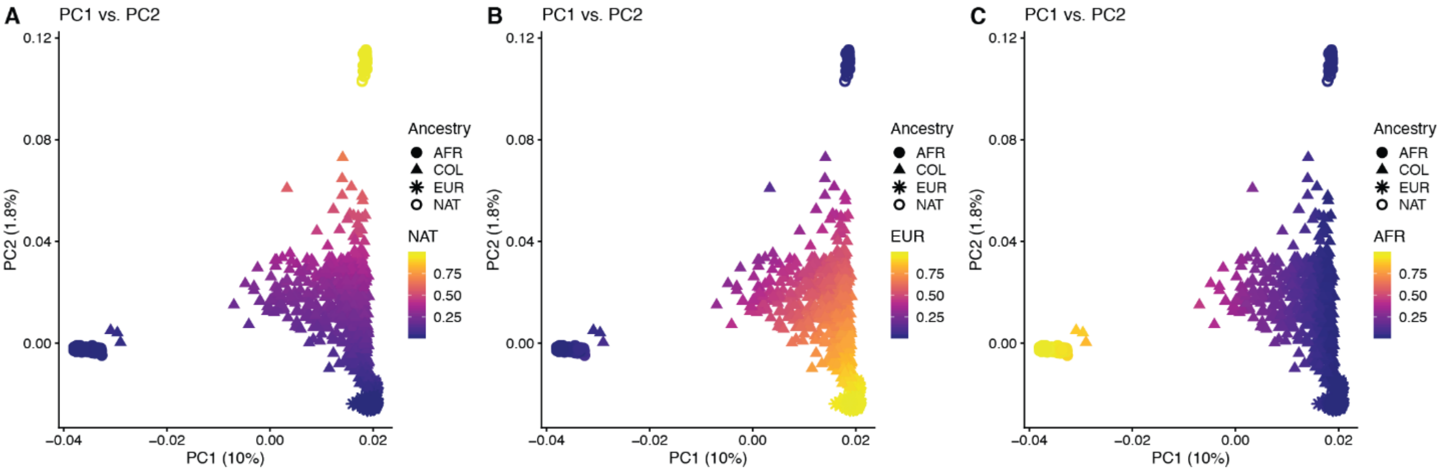

Figure S9

Principal component analyses of the African and European cohorts of the 1000GP, along with 43 native American genomes and the TANGL cohort colored according to their proportions of global ancestry.

Individuals are colored according to their proportion of the genome estimated to be Native American **(A)**, European **(B)** and African **(C)** by ADMIXTURE at K=3. AFR: African, COL: Colombian, EUR: European, NAT: Native American

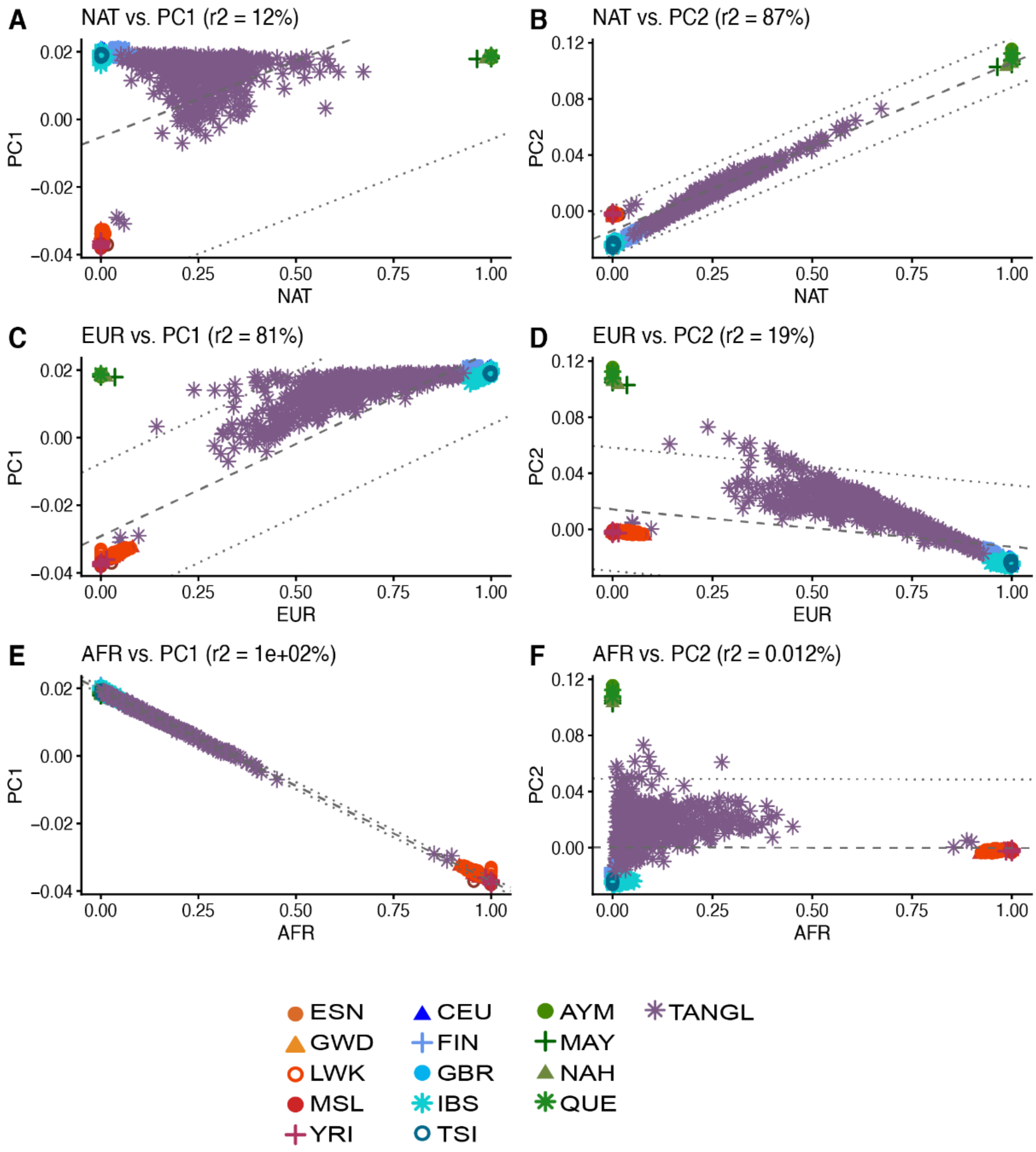

Figure S10

Correlation of the principal component 1 and 2 values and the global ancestry proportions. For the TANGL.AFR.EUR.NAT cohort

Correlation of global ancestry proportion of the genome estimated to be Native American **(A and B)**, European **(C and D)** and African **(E and F)** by ADMIXTURE at K=3 with principal component 1 **(A, C and E)** and principal component 2 **(B, D and F)**. AFR: African (orange), EUR: European (blue), NAT: Native American (green). r2: Pearson´s coefficient of determination**.** ESN: Esan in Nigeria. GWD: Gambian in Western Divisions in the Gambia. LWK: Luhya in Webuye, Kenya. MSL: Mende in Sierra Leone. YRI: Yoruba in Ibadan, Nigeria. CEU: Utah Residents (CEPH) with Northern and Western European Ancestry. FIN: Finnish in Finland. GBR: British in England and Scotland. IBS: Iberian Population in Spain. TSI: Toscani in Italia. AYM: Aymara. MAY: Mayan, NAH: Náhuatl. QUE: Quechua.

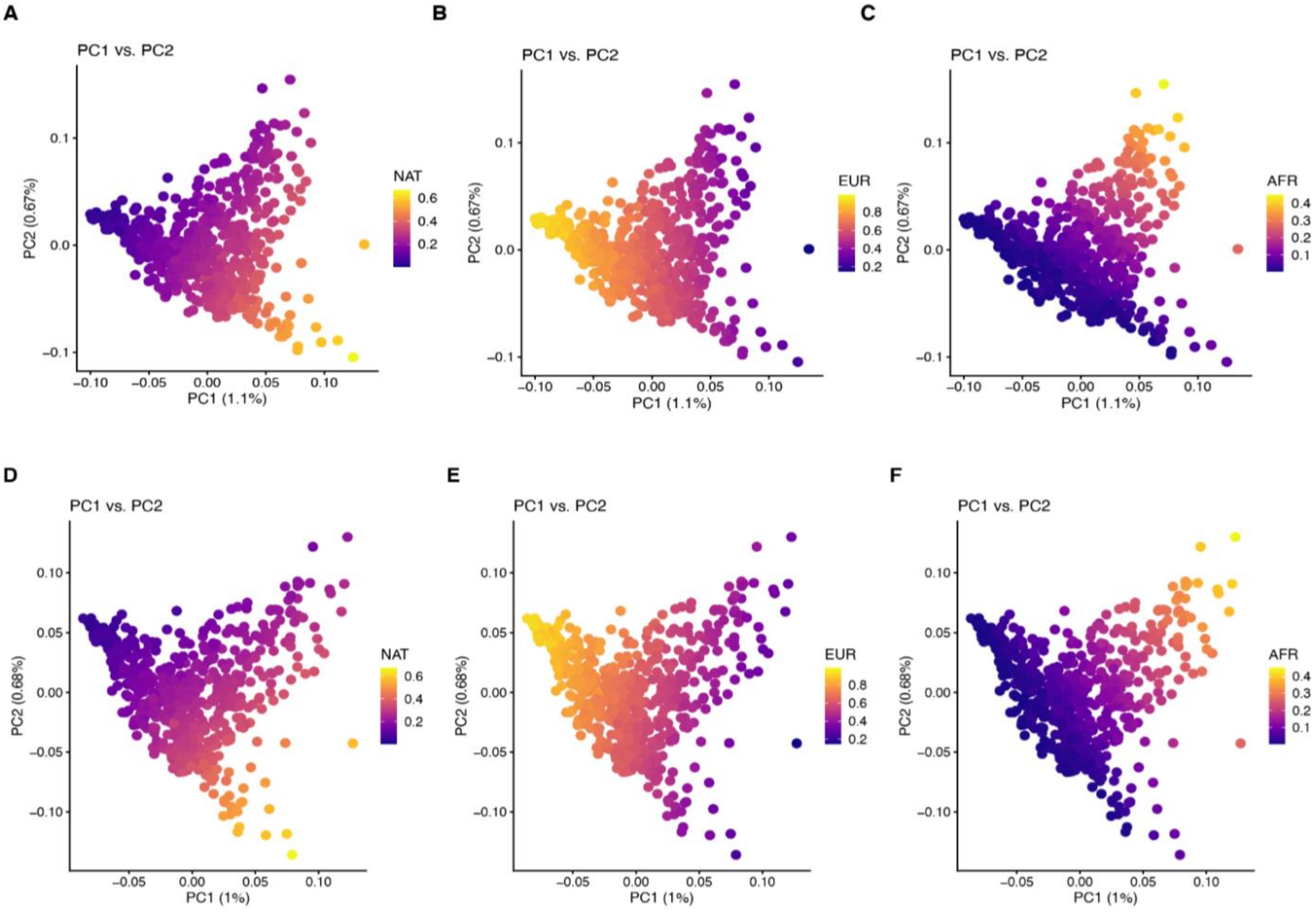

Figure S11

Principal component analyses of the TANGL cohort colored according to their proportions of global ancestry.

Principal component analyses TANGL cohort retaining variants with MAF >10% **(A, B and C)** and MAF between 5 – 10% **(D, E and F)**. Individuals are colored according to their proportion of the genome estimated to be Native American **(A and D)**, European **(B and E)** and African **(C and F)** by ADMIXTURE at K=3. AFR: African, COL: Colombian, EUR: European, NAT: Native American

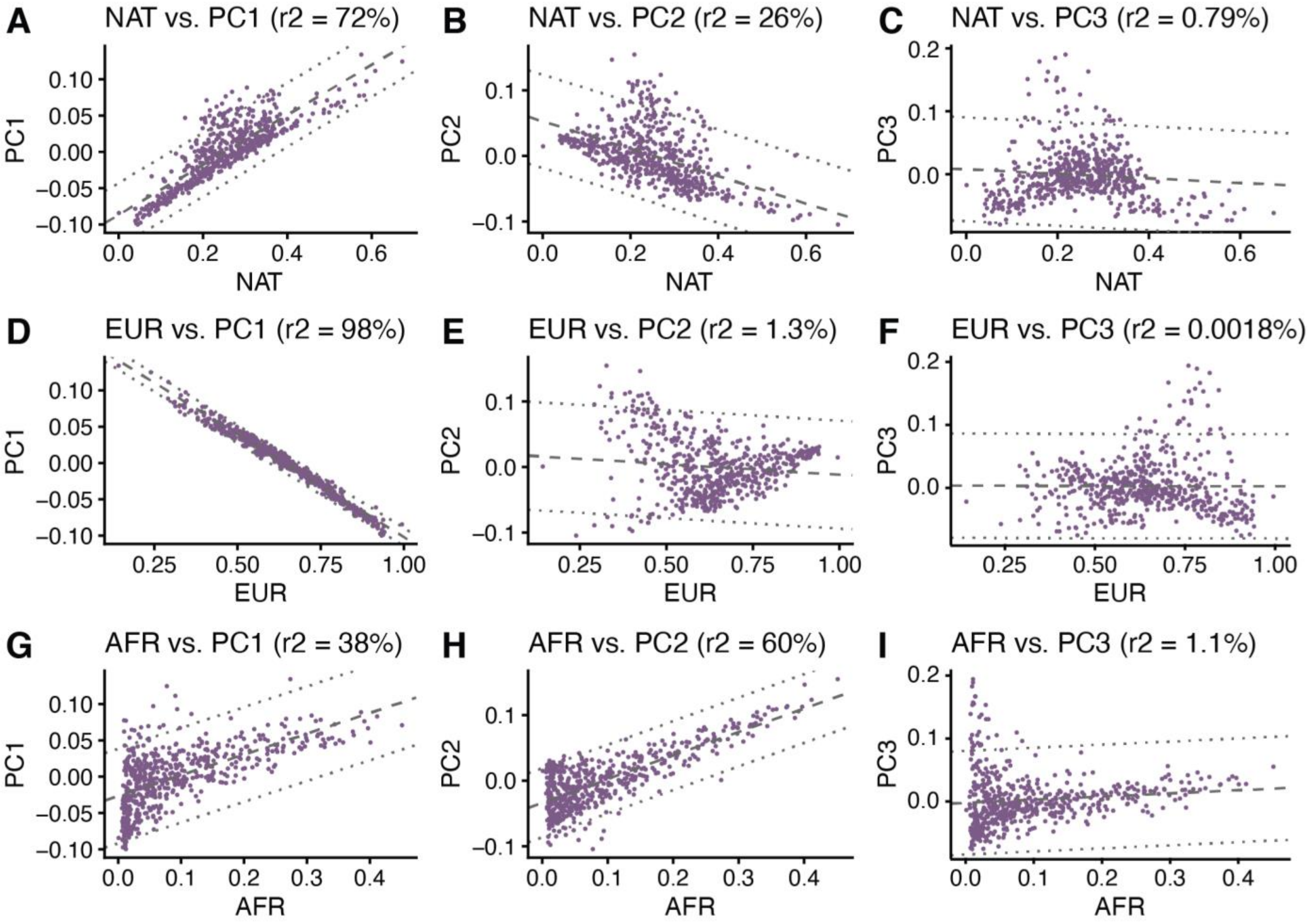

Figure S12

Correlation of the principal component 1 and 2 values and the global ancestry proportions for the TANGL cohort using common variants (MAF >10%).

Correlation of global ancestry proportion of the genome estimated to be Native American **(A, B and C)**, European **(D, E and F)** and African **(G, H and I)** by ADMIXTURE at K=3 with Principal components calculated with variants of MAF >10%. Ancestral proportion vs principal component 1 **(A, D and G)**, principal component 2 **(B, E and H)** and principal component 3 **(C, F and I)**. AFR: African, EUR: European, NAT: Native American. r2 : Pearson's coefficient of determination**.**

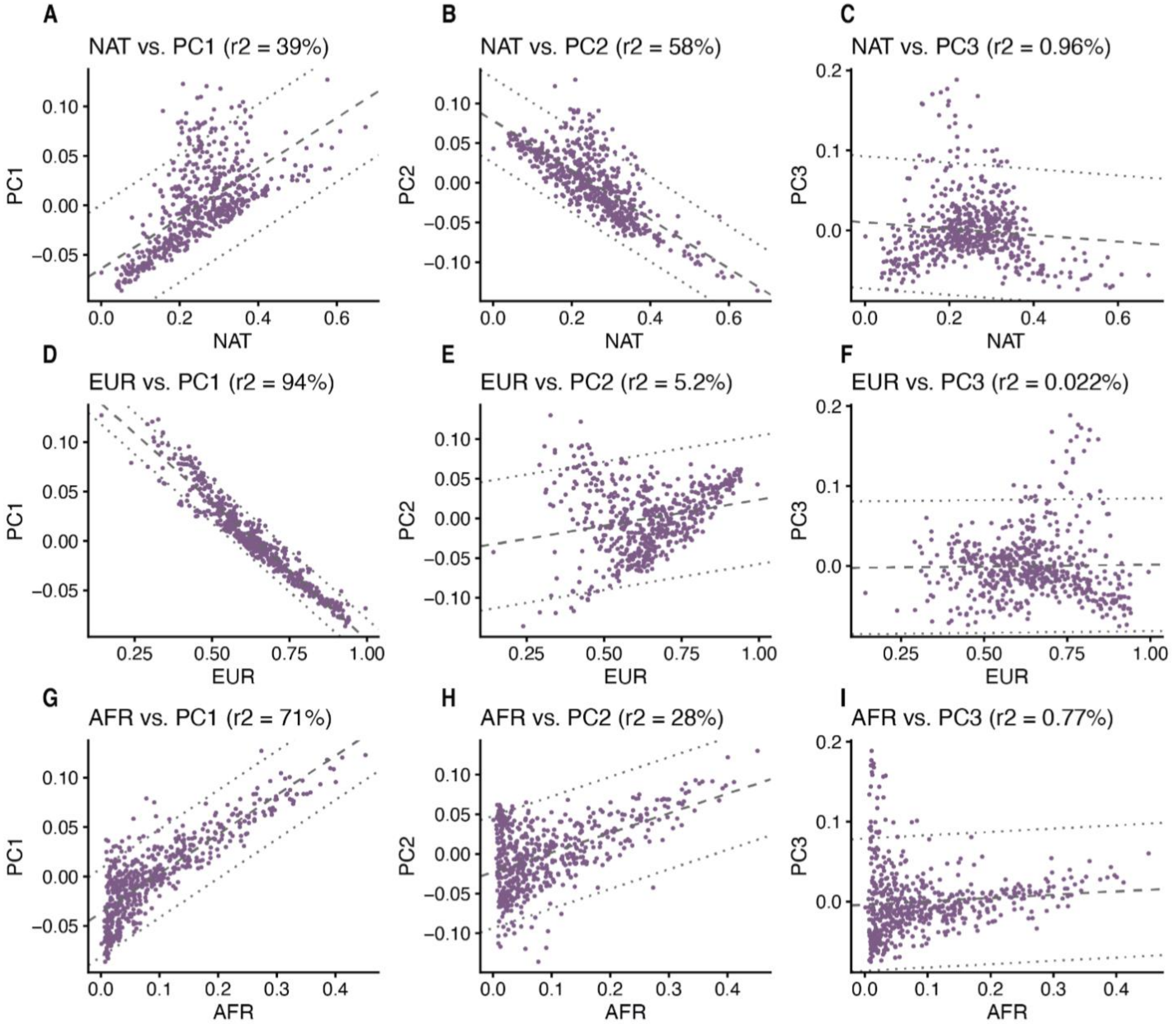

Figure S13

Correlation of the principal component 1 and 2 values and the global ancestry proportions for the TANGL cohort using common variants (MAF 5-10%).

Correlation of global ancestry proportion of the genome estimated to be Native American **(A, B and C)**, European **(D, E and F)** and African **(G, H and I)** by ADMIXTURE at K=3 with Principal components calculated with variants of MAF 5 - 10%. Ancestral proportion vs principal component 1 **(A, D and G)**, principal component 2 **(B, E and H)** and principal component 3 **(C, F and I)**. AFR: African, EUR: European, NAT: Native American. r2 : Pearson´s coefficient of determination**.**

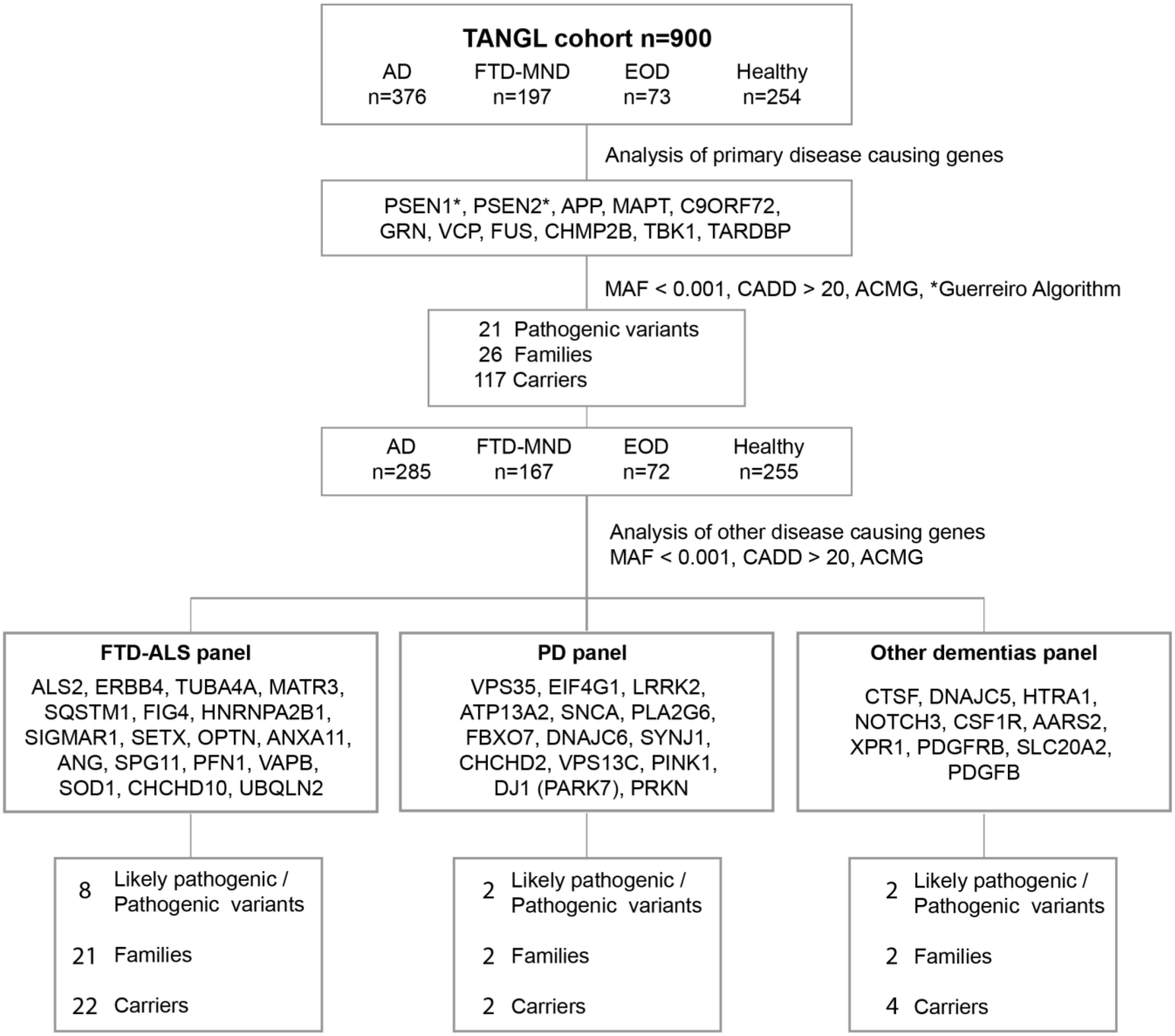

Figure S14

Pipeline of the curation of disease-causing variants in the TANGL cohort.

Genes associated with AD and FTD were selected from the AD/FTD mutation databases (https://www.molgen.vib-ua.be/ADMutations and https://www.alzforum.org/mutations). A secondary genetic analysis was done to identify possibly pathogenic variants in other genes associated with similar or overlapping phenotypes. For the secondary screening, the disease-causing genes included were those reported in the following OMIM phenotype series and phenotypes: Frontotemporal dementia and/or Amyotrophic Lateral Sclerosis (PS105550, PS167320, PS105400), Parkinson disease (PS168600), Adult-Onset Leukoencephalopathies (PS125310, 221820) and, Ceroid lipofuscinoses (PS256730).

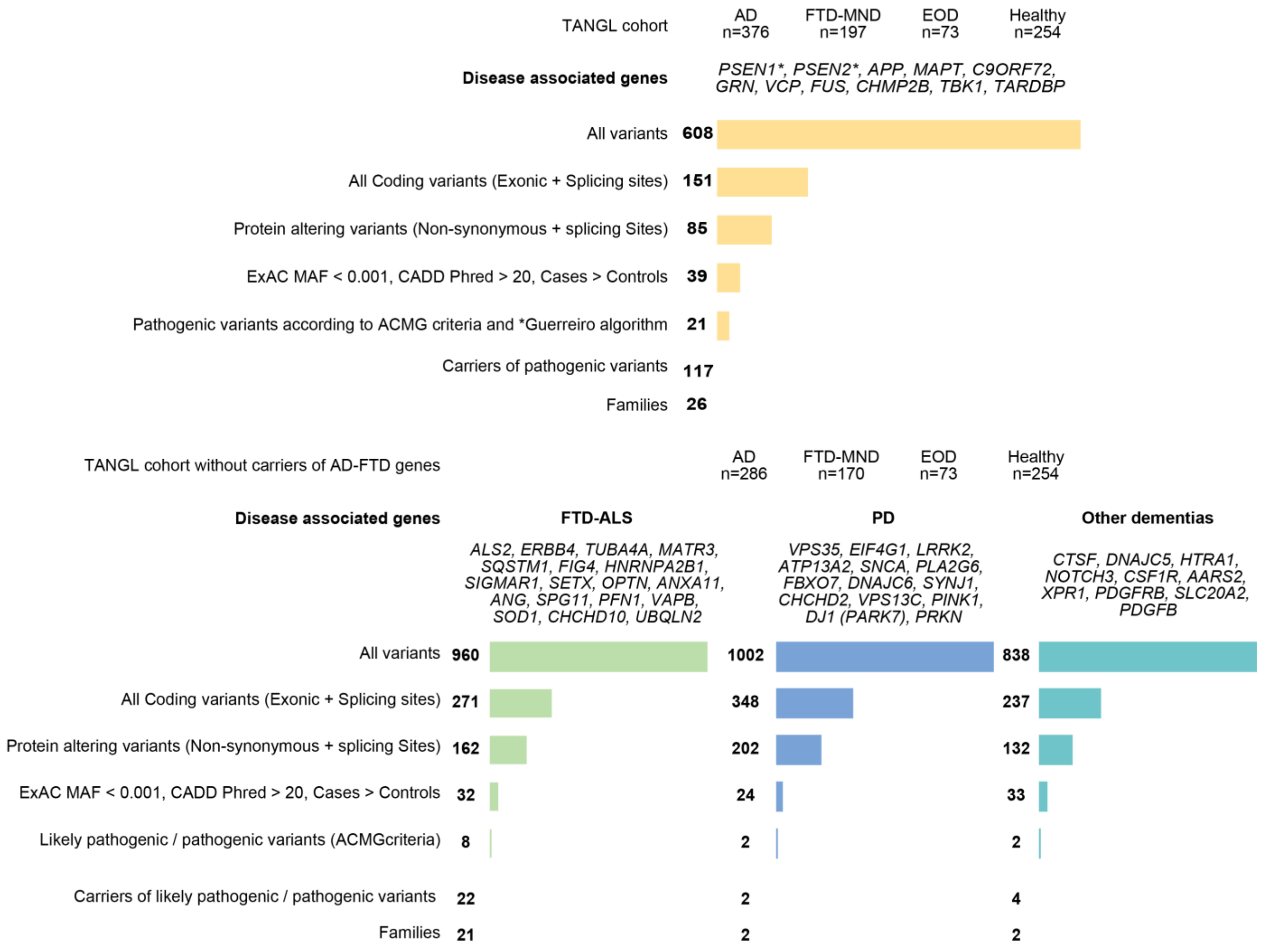

Figure S15

Variant filtering of disease-causing variants in the TANGL cohort

Top row: Genes associated with AD and FTD were selected from the AD/FTD mutation databases (https://www.molgen.vib-ua.be/ADMutations and https://www.alzforum.org/mutations). **Bottom row:** Variants in other genes associated with similar or overlapping phenotypes. Frontotemporal dementia and/or Amyotrophic Lateral Sclerosis (OMIM PS105550, PS167320, PS105400), Parkinson disease (OMIM PS168600), Adult-Onset Leukoencephalopathies (OMIM PS125310, 221820) and, Ceroid lipofuscinoses (OMIM PS256730).

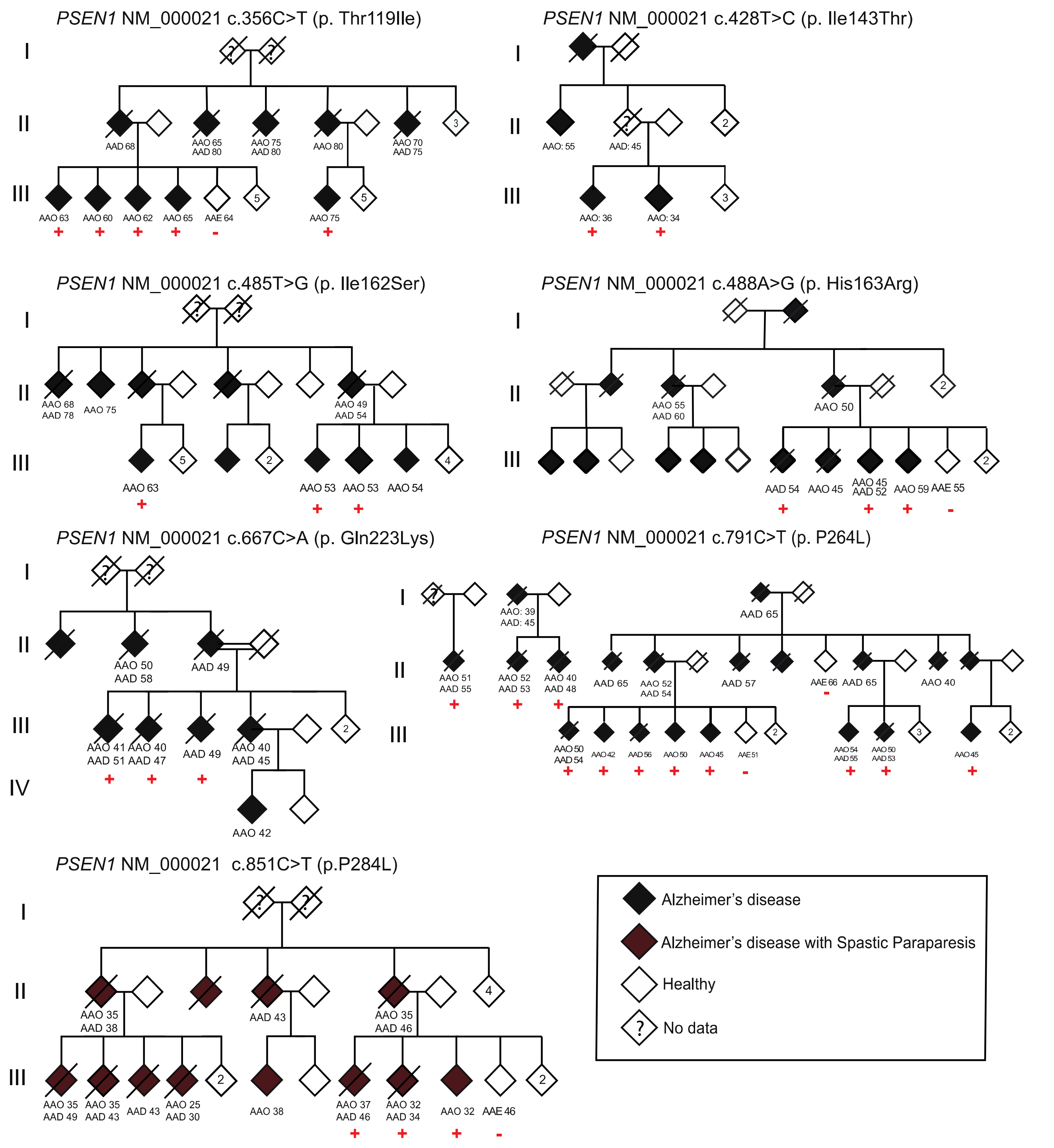

Figure S16

Pedigrees of the families with pathogenic variants in *PSEN1* (NM_000021).

Affected individuals are represented as filled icons; empty icons represent asymptomatic individuals. Deceased individuals are crossed out. Numbers inside icons indicate number of siblings. The gender of the individuals was omitted for privacy. AAO: Age at onset, AAD: Age at death. + known carriers, - known non-carriers. Pedigrees of the Colombian families with *PSEN1* Pro117Ala, Glu280Ala and Ile 416Thr have been documented elsewhere.

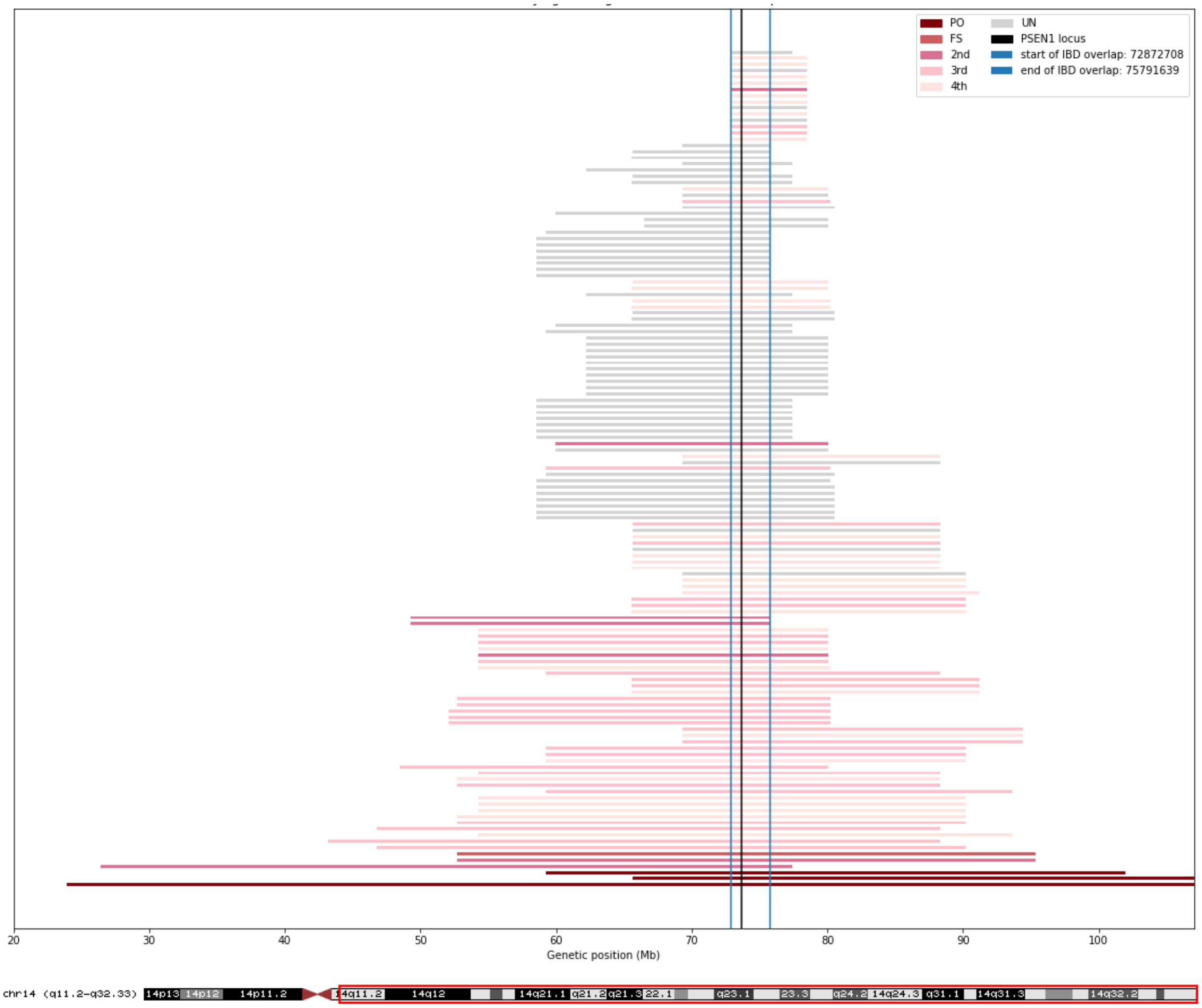

Figure S17
Pairwise identity by Descent (IBD) segments in the chromosomes that harbor the *PSEN1* NM_000021 c.791C>T (p. Pro264Leu) variant.
Horizontal lines represent IBD haplotypes >2cM detected using Hap-IBD. The blue vertical lines indicate the portion of IBD segments shared among all the carriers, and the black vertical line indicates the locus of the *PSEN1* gene in chromosome 14. Each horizontal line represents a pairwise IBD segment between two carriers and is colored according to their relationship coefficient (r) calculated by KING. PO: Parent-offspring (r=0.5), FS: Full siblings(r=0.5), 2nd: Second degree of relatedness (r=0.25), 3rd: third degree of relatedness (r=0.125), 4th: Fourth degree of relatedness (r=0.0625). UN: Individuals beyond 4th degree of relatedness (r<0.0625)

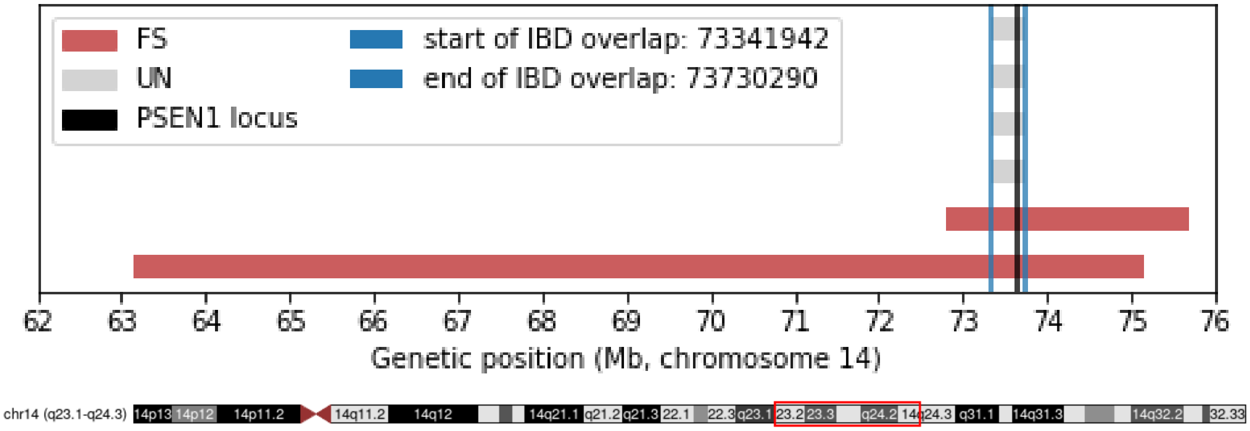

Figure S18

Pairwise identity by Descent (IBD) segments in the chromosomes that harbor the *PSEN1* NM_000021 c.428T>C (p.Ile143Thr) variant.

Horizontal lines represent IBD haplotypes >2cM detected using Hap-IBD. The blue vertical lines indicate the portion of IBD segments shared among all the carriers, and the black vertical line indicates the locus of the *PSEN1* gene in chromosome 14. Each horizontal line represents a pairwise IBD segment between two carriers and is colored according to their relationship coefficient (r) calculated by KING. FS: Full siblings(r=0.5), UN: Individuals beyond 4th degree of relatedness (r<0.0625)

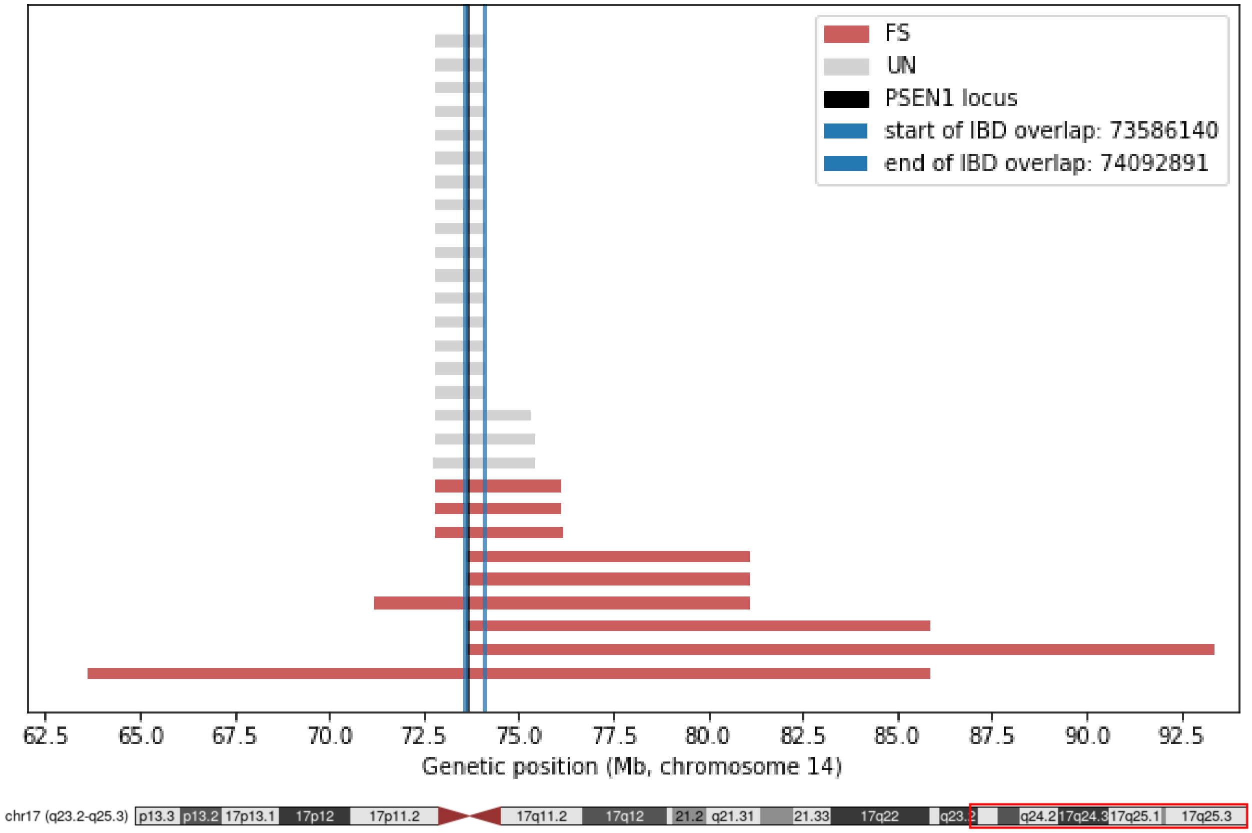

Figure S19

Pairwise identity by Descent (IBD) segments in the chromosomes that harbor the *PSEN1* NM_000021 c.356C>T (p.Thr119Ile) variant in Colombian individuals.

Horizontal lines represent IBD haplotypes >2cM detected using Hap-IBD. The blue vertical lines indicate the portion of IBD segments shared among all the carriers, and the black vertical line indicates the locus of the *PSEN1* gene in chromosome 14. Each horizontal line represents a pairwise IBD segment between two carriers and is colored according to their relationship coefficient (r) calculated by KING. FS: Full siblings(r=0.5), UN: Individuals beyond 4th degree of relatedness (r<0.0625)

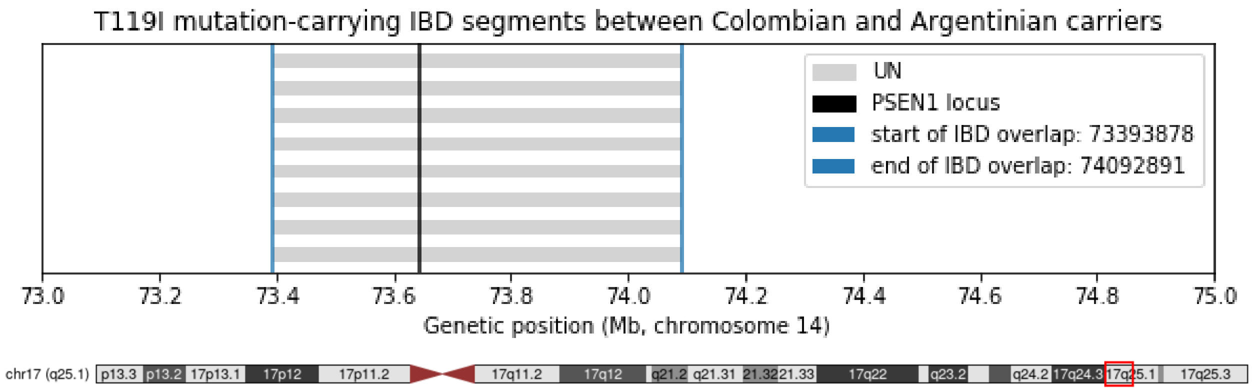

Figure S20

Pairwise identity by Descent (IBD) segments carrying the *PSEN1* NM_000021 c.356C>T (p.Thr119Ile) variant in Colombian and Argentinian individuals.

Each Horizontal line represents a shared haplotype >2cM, IBD between the Argentinian sample and a Colombian carrier of the same variant. The IBD segments were detected using Hap-IBD. The blue vertical lines indicate the portion of IBD segments shared among all the carriers, and the black vertical line indicates the locus of the *PSEN1* gene in chromosome 14. Each horizontal line represents a pairwise IBD segment between two carriers and is colored according to their relationship coefficient (r) calculated by KING. UN: Individuals beyond 4th degree of relatedness (r<0.0625)

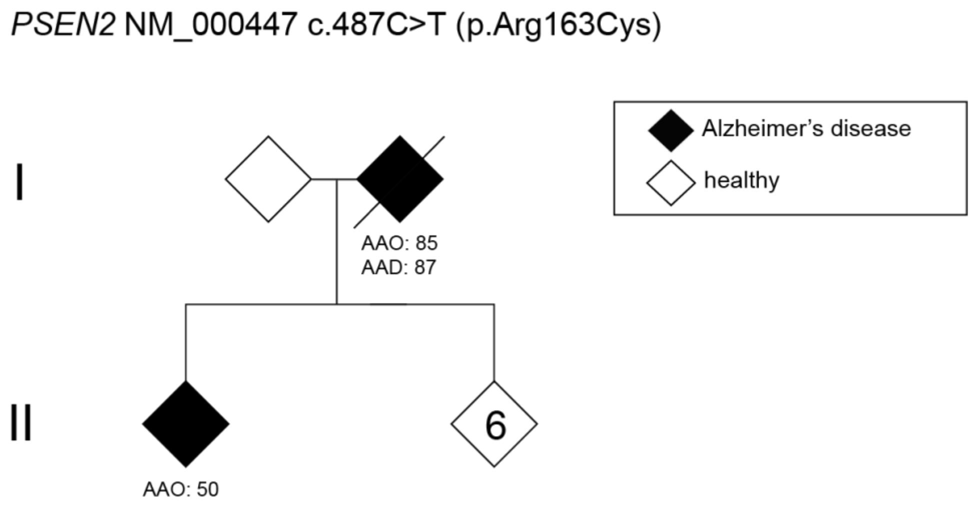

Figure S21
Pedigrees of the family with a pathogenic variant in *PSEN2* (NM_000447).
Affected individuals are represented as filled icons; empty icons represent asymptomatic individuals. Deceased individuals are crossed out. Numbers inside icons indicate number of siblings. The gender of the individuals was omitted for privacy. AAO: Age at onset, AAD: Age at death. No cosegregation data available for this family

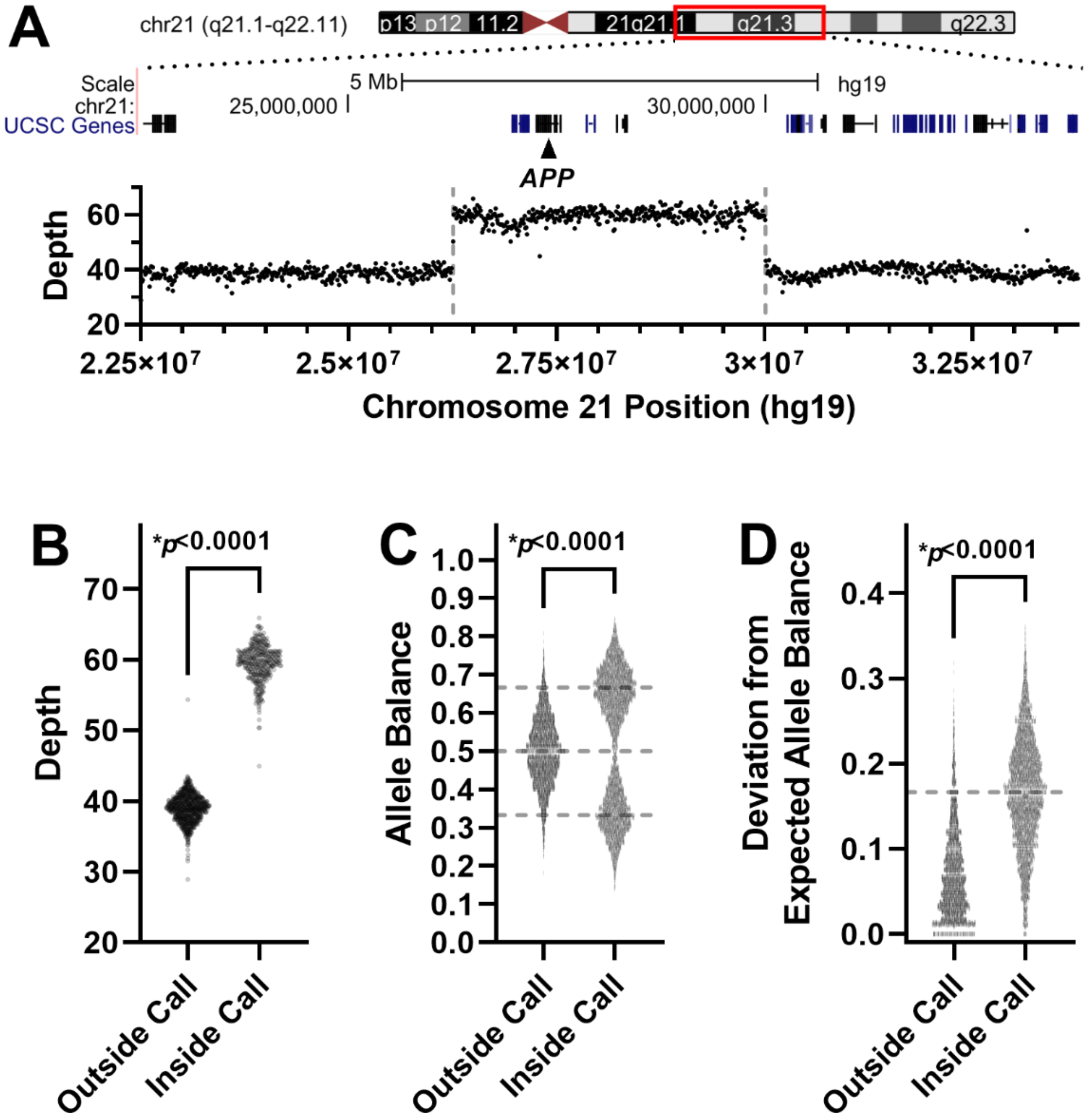

Figure S22
Depth and allele balance indicate a duplication including *APP*.
(A) Sequencing read depth demonstrating a 3.76 MB duplication that includes the full *APP* gene (hg19 position). This copy number variant was called by four independent callers (DELLY, ERDS, CNVnator, and BIC-seq2). **(B)** Sequencing depth is significantly higher within the called region ($p<0.0001$, Mann-Whitney). **(C)** The distribution of allele balance is significantly different within the called region ($p<0.0001$, Kolmogorov-Smirnov). **(D)** Displayed another way, as deviation of the expected allele balance of 0.5, the distribution of allele balance is significantly different within the called region ($p<0.0001$, Mann-Whitney).

MAPT NM_005910 c.902C>T (p.Pro301Leu)

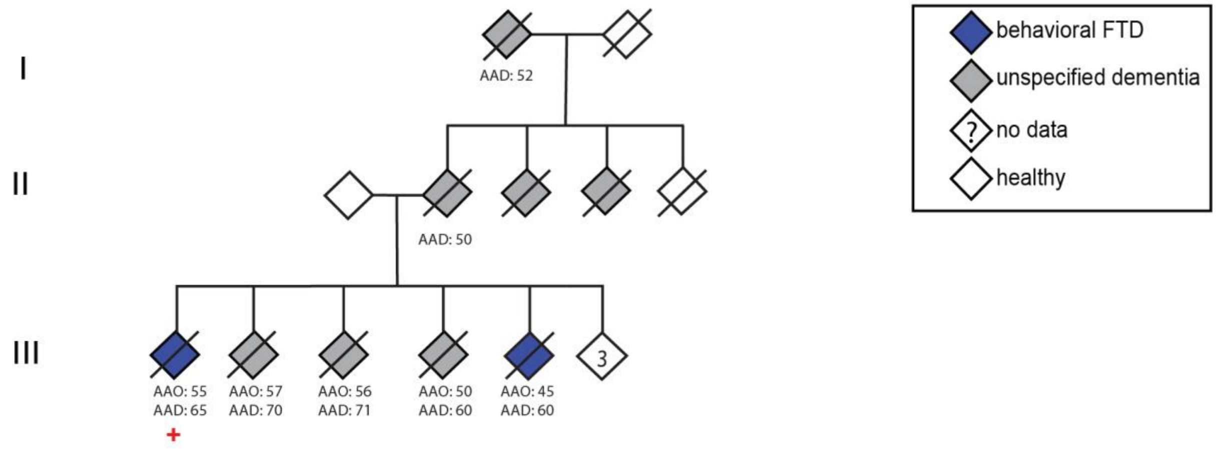

MAPT NM_005910 c.1189C>T (p.Pro397Ser)

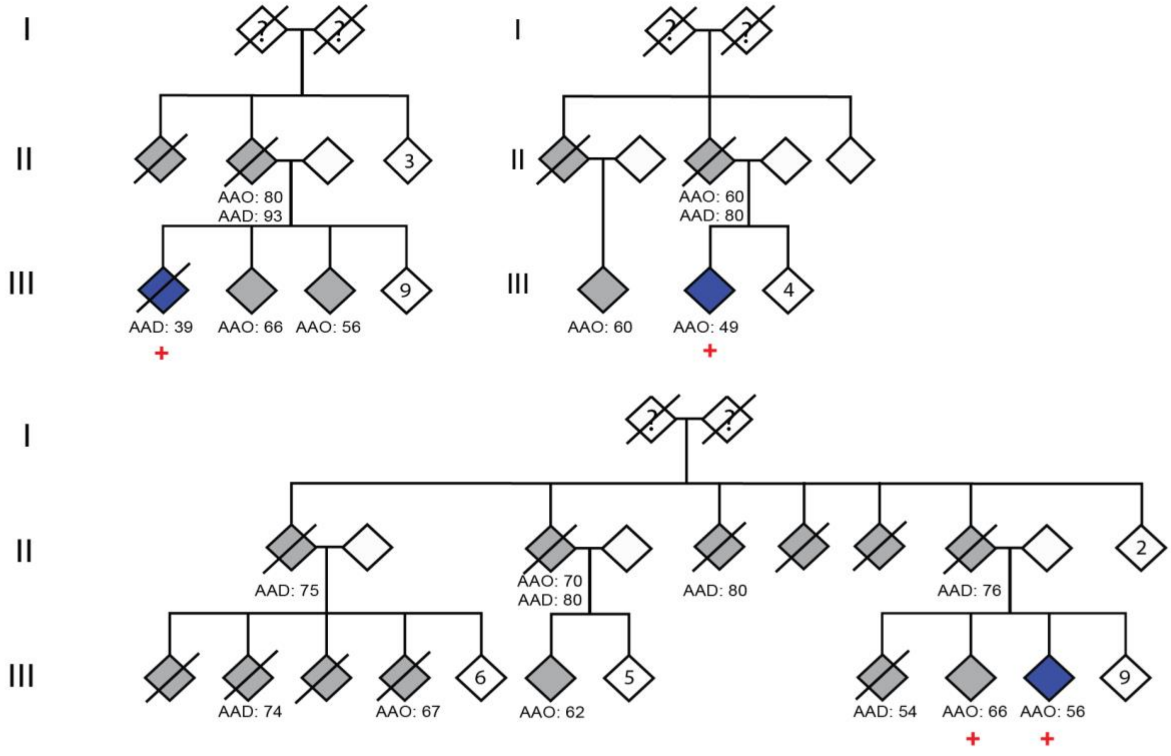

Figure S23
Pedigrees of the families with pathogenic variants in MAPT (NM_005910).
Affected individuals are represented as filled icons; empty icons represent asymptomatic individuals, numbers inside icons represent number of healthy siblings. Deceased individuals are crossed out. + known carriers, - known non-carriers. The gender of the individuals was omitted for privacy AAO: Age at onset, AAD: Age at death, FTD: Frontotemporal Dementia.

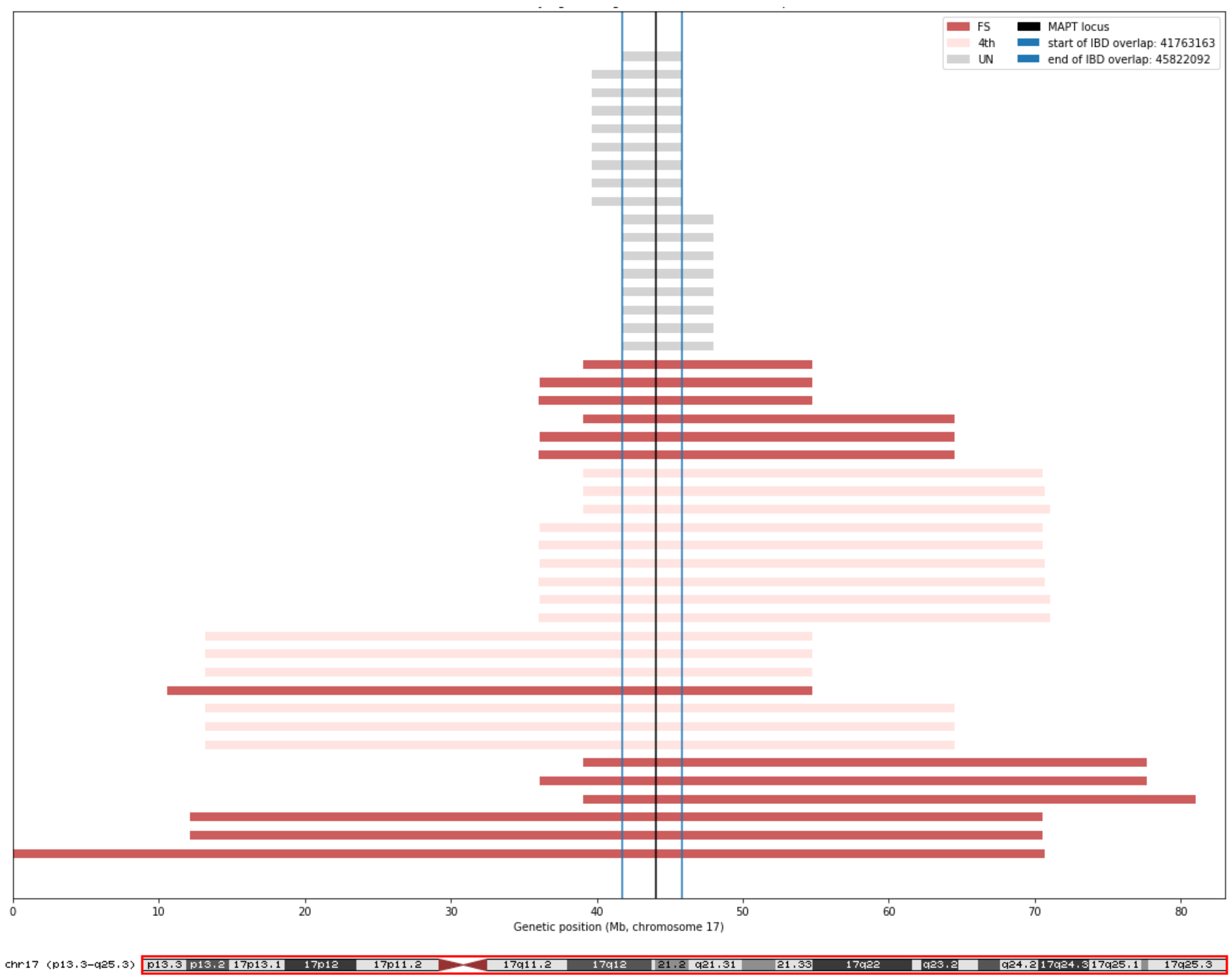

Figure S24

Pairwise identity by Descent (IBD) segments in the chromosomes that harbor the MAPT NM_005910 c.1189C>T (p.Pro397Ser) variant.

Horizontal lines represent IBD haplotypes >2cM detected using Hap-IBD. The blue vertical lines indicate the portion of IBD segments shared among all the carriers, and the black vertical line indicates the locus of the *MAPT* gene in chromosome 17. Each horizontal line represents a pairwise IBD segment between two carriers and is colored according to their relationship coefficient (r) calculated by KING. FS: Full siblings(r=0.5), 3rd: third degree of relatedness (r=0.125). UN: Individuals beyond 4th degree of relatedness (r<0.0625)

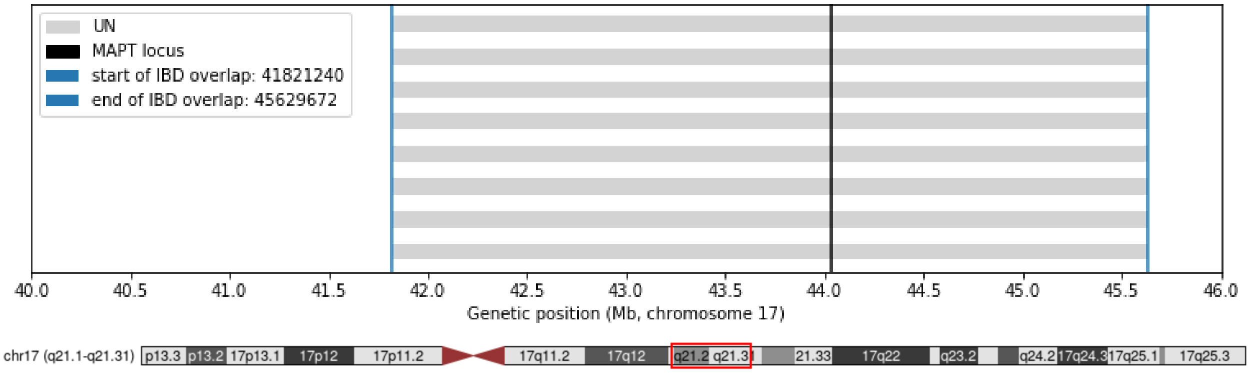

Figure S25

Pairwise identity by Descent (IBD) segments in the chromosomes that harbor the MAPT NM_005910 c.1189C>T (p.Pro397Ser) variant from Colombian and Spanish families.

Horizontal lines represent IBD haplotypes >2cM detected using Hap-IBD. The blue vertical lines indicate the portion of IBD segments shared among all the carriers, and the black vertical line indicates the locus of the *MAPT* gene in chromosome 17. Each horizontal line represents a pairwise IBD segment between two carriers and is colored according to their relationship coefficient (r) calculated by KING. FS: Full siblings(r=0.5), 3rd: third degree of relatedness (r=0.125). UN: Individuals beyond 4th degree of relatedness (r<0.0625)

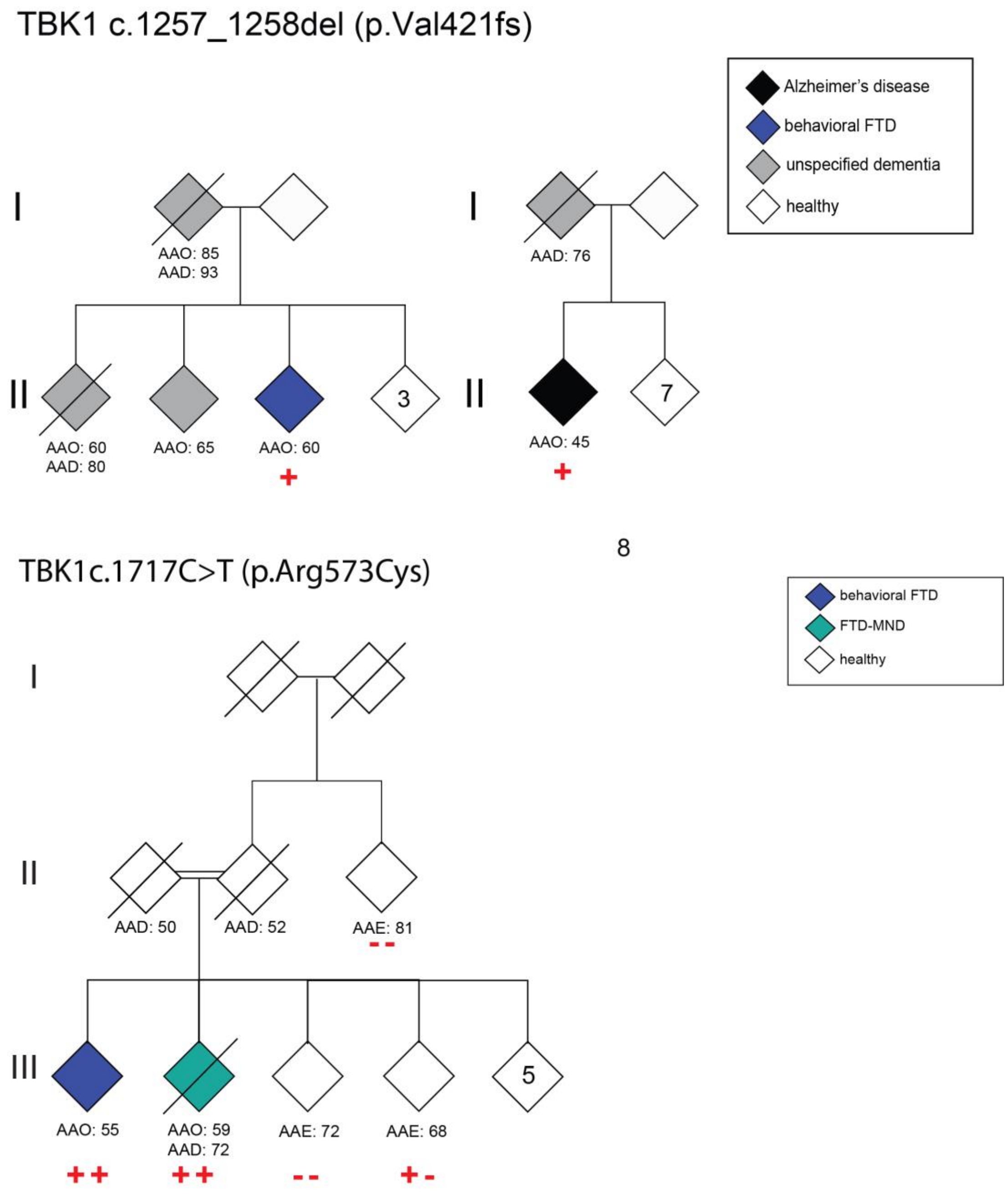

Figure S26

Pedigrees of the families with pathogenic variants in *TBK1* (NM_013254).

Affected individuals are represented as filled icons; empty icons represent asymptomatic individuals, numbers inside icons represent number of healthy siblings. Deceased individuals are crossed out. The gender of the individuals was omitted for privacy. AAO: Age at onset, AAD: Age at death, AAE: age at evaluation FTD: Frontotemporal Dementia, FTD-MND: Frontotemporal dementia with motor neuron disease. For c.1257_1258del (p.Val421Cfs*26) + represents known heterozygous carriers. For c.1717C>T (p.Arg573Cys) ++ represents homozygous alternate, +- heterozygous and - - homozygous reference individuals.

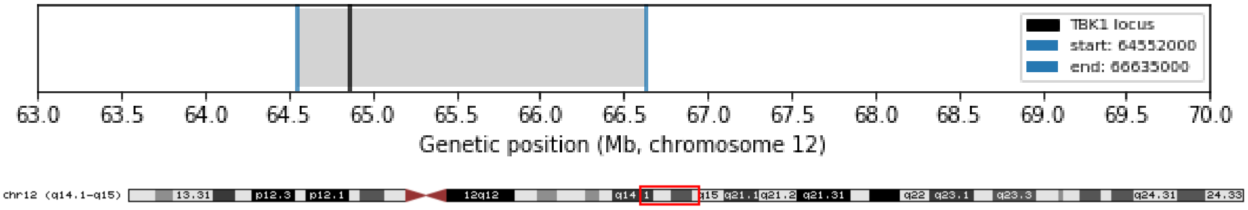

Figure S27

Pairwise identity by Descent (IBD) segments in the chromosomes that harbor TBK1 NM_013254 c.1257_1258del (p.Val421Cfs) variant.

The horizontal gray line represents the pairwise IBD segment >2cM detected using Hap-IBD. The blue vertical lines indicate the portion of IBD segment shared among both carriers, and the black vertical line indicates the locus of the *TBK1* gene in chromosome 12.

TARDBP NM_007375 c.1147A>G (p.Ile383Val)

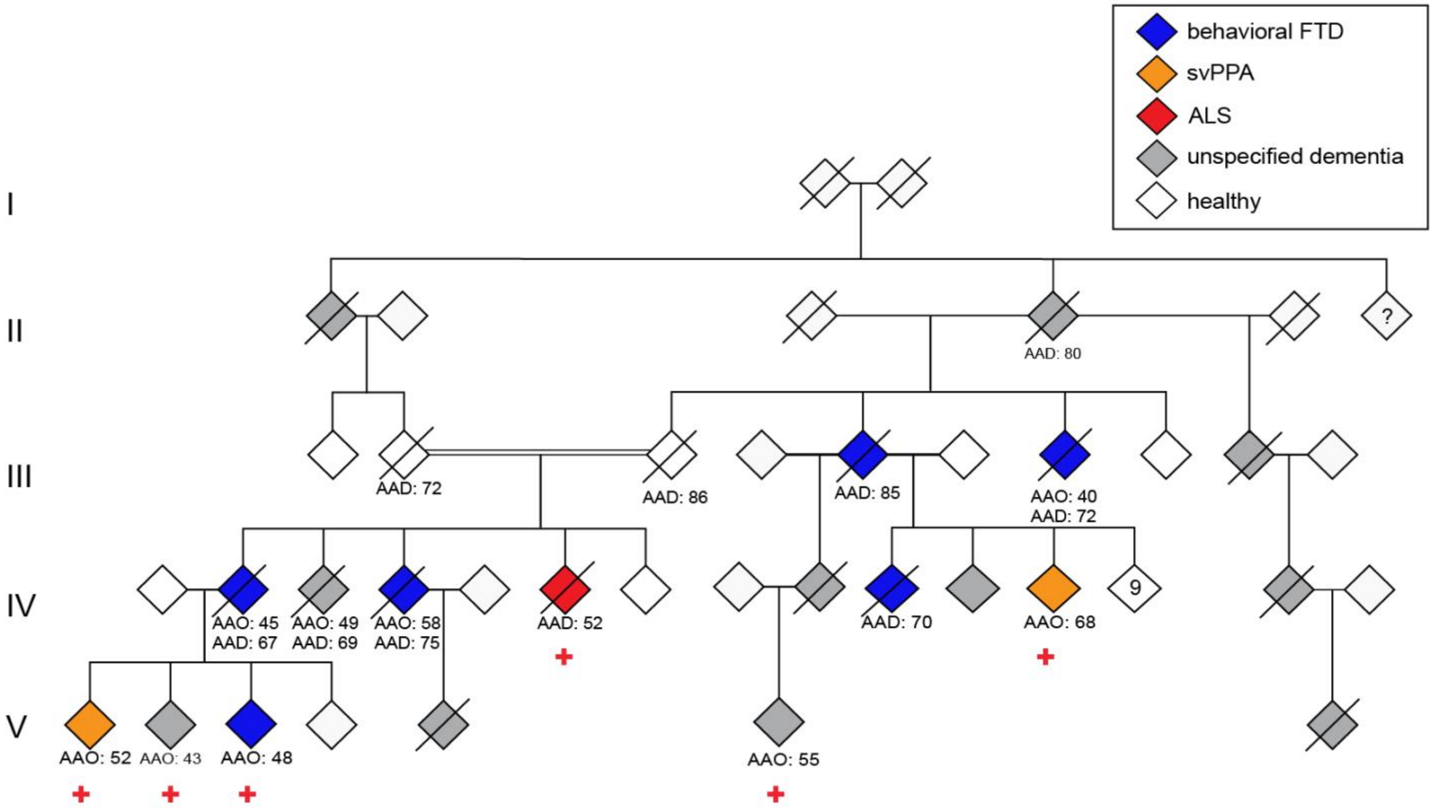

Figure S28
Pedigree of the family with a pathogenic variant in *TARDBP* (NM_007375).
Affected individuals are represented as filled icons; empty icons represent asymptomatic individuals, numbers inside icons represent number of healthy siblings. Deceased individuals are crossed out. The gender of the individuals was omitted for privacy. AAO: Age at onset, AAD: Age at death, FTD: Frontotemporal Dementia, svPPA: Semantic variant of primary progressive aphasia, ALS: Amyotrophic lateral sclerosis. + known carriers, - known non-carriers.

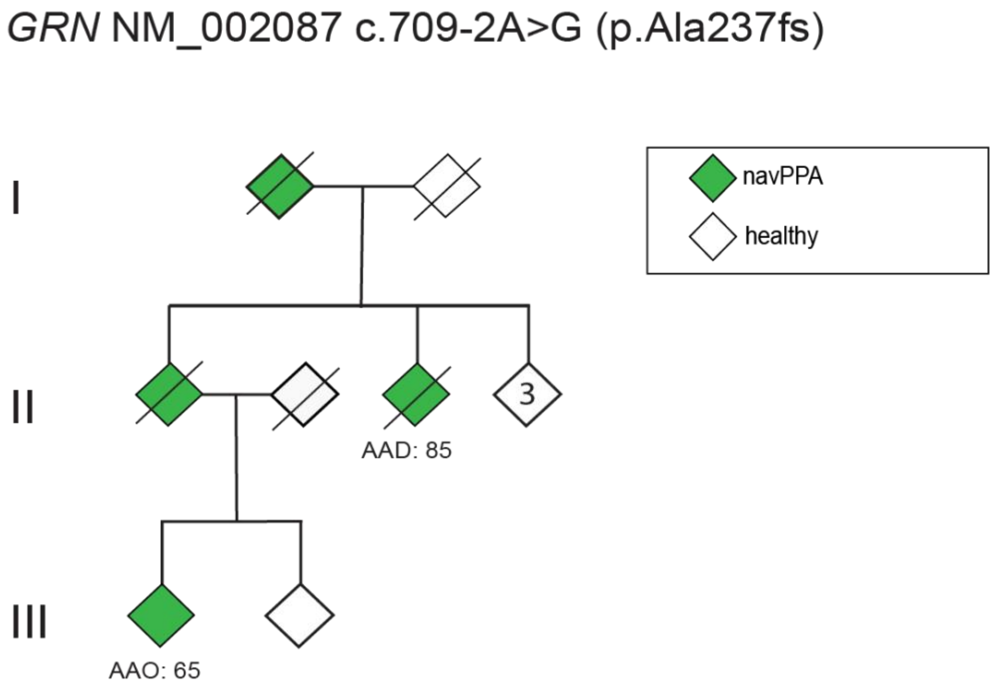

Figure S29
Pedigree of the family with a pathogenic variant in *GRN* (NM_002087).
Affected individuals are represented as filled icons; empty icons represent asymptomatic individuals, numbers inside icons represent number of healthy siblings. Deceased individuals are crossed out. The gender of the individuals was omitted for privacy. AAO: Age at onset, AAD: Age at death, navPPA: primary progressive aphasia-non-fluent/agrammatic variant. No cosegregation data available for this family

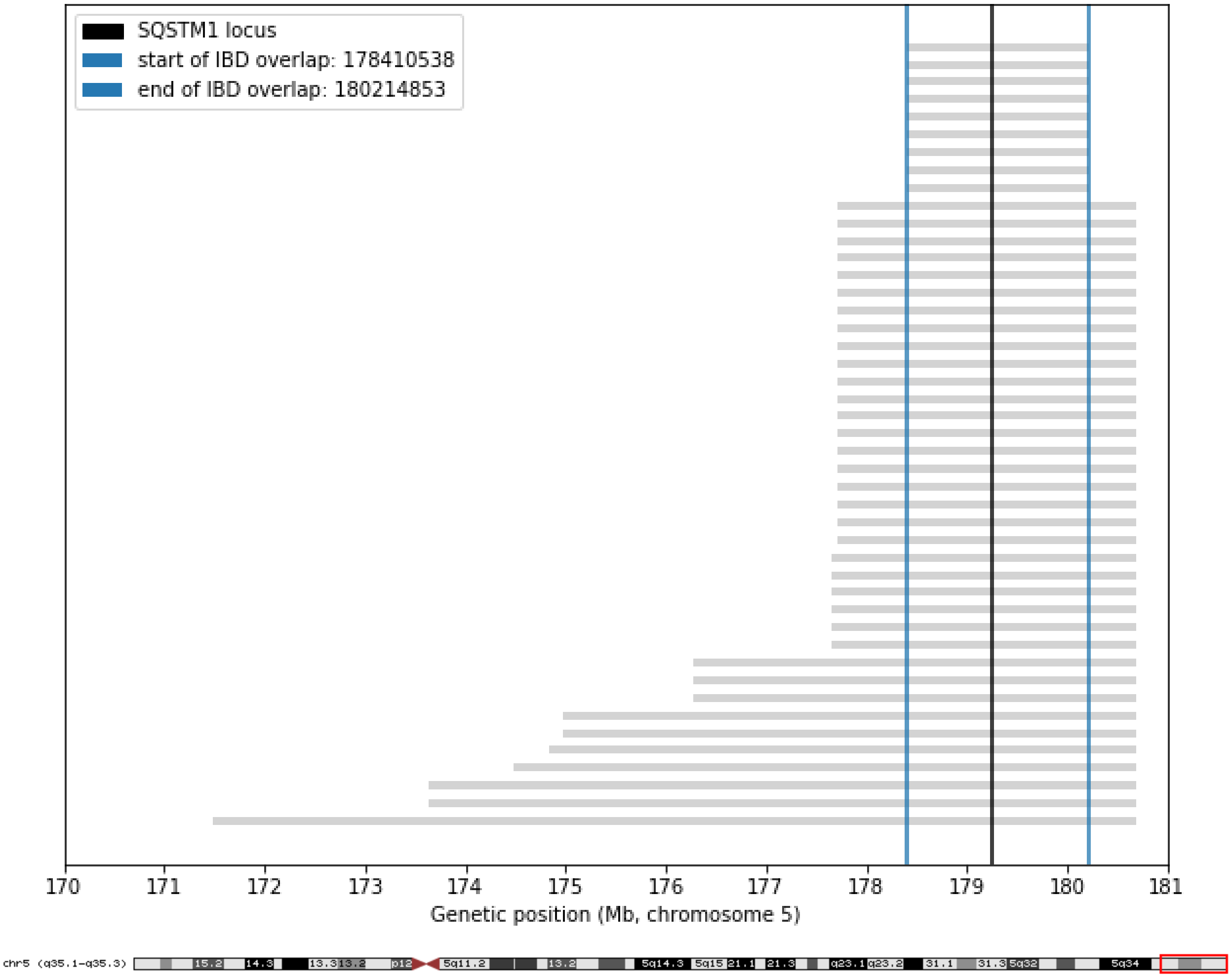

Figure S30

Pairwise identity by Descent (IBD) segments in the chromosomes that harbor *SQSTM1* NM_003900 c.1175C>T (p.Pro392Leu) variant in the TANGL cohort.

Horizontal lines represent IBD haplotypes >2cM detected using Hap-IBD. The blue vertical lines indicate the portion of IBD segments shared among all the carriers, and the black vertical line indicates the locus of the *SQSTM1* gene in chromosome 5. Each horizontal line represents a pairwise IBD segment between two carriers and is colored according to their relationship coefficient (r) calculated by KING. Gray lines represent unrelated individuals (UN) or beyond 4th degree of relatedness (r<0.0625). There was no IBD segment >2cM between the TANGL and the 1000GP *SQSTM1* Pro392Leu carriers.

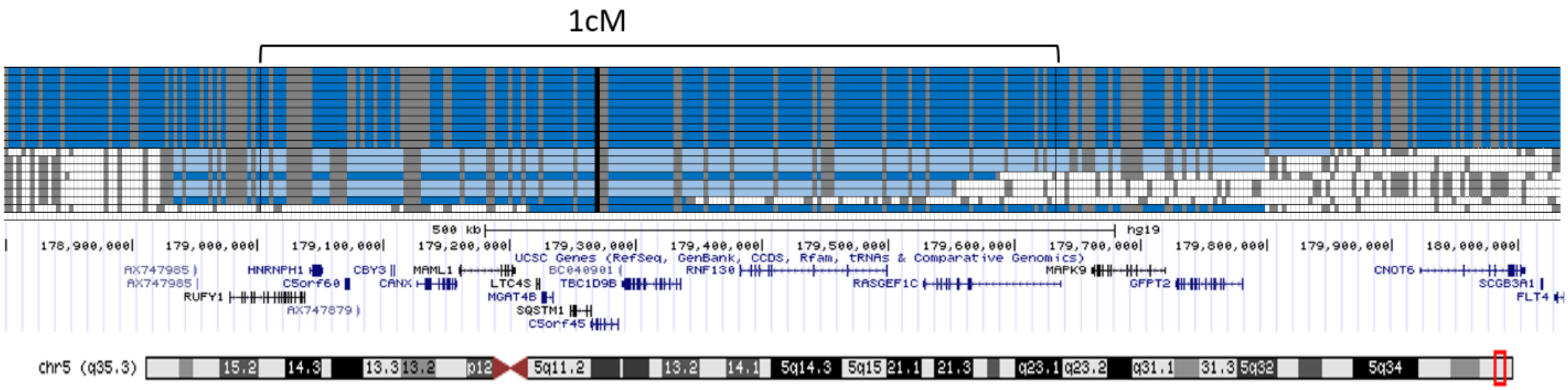

Figure S31

Alignment of the haplotypes that harbor *SQSTM1* NM_003900 c.1175C>T (p.Pro392Leu) variant in the TANGL and the 1000GP cohort.

Alignment of thirteen variant carrying haplotypes from the TANGL cohort (dark blue) and 1000GP (light blue). Black vertical line represents the location of the variant of interest. cM: centimorgans

TUBA4A NM_006000 c.820C>G (p.Pro274Ala)

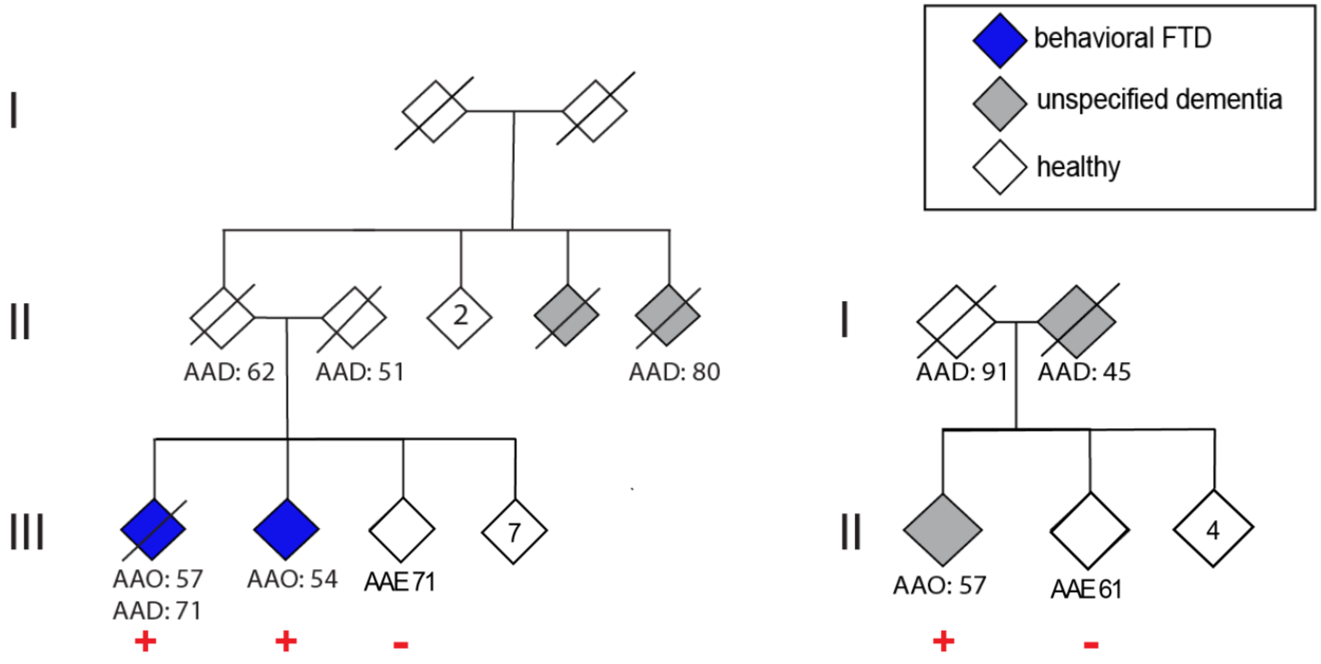

Figure S32
Pedigrees of the families with pathogenic variants in *TUBA4A* (NM_006000).
Affected individuals are represented as filled icons; empty icons represent asymptomatic individuals, numbers inside icons represent number of healthy siblings. Deceased individuals are crossed out. The gender of the individuals was omitted for privacy. AAO: Age at onset, AAD: Age at death, AAE: Age at evaluation FTD: frontotemporal dementia. + known carriers, - known non-carriers.

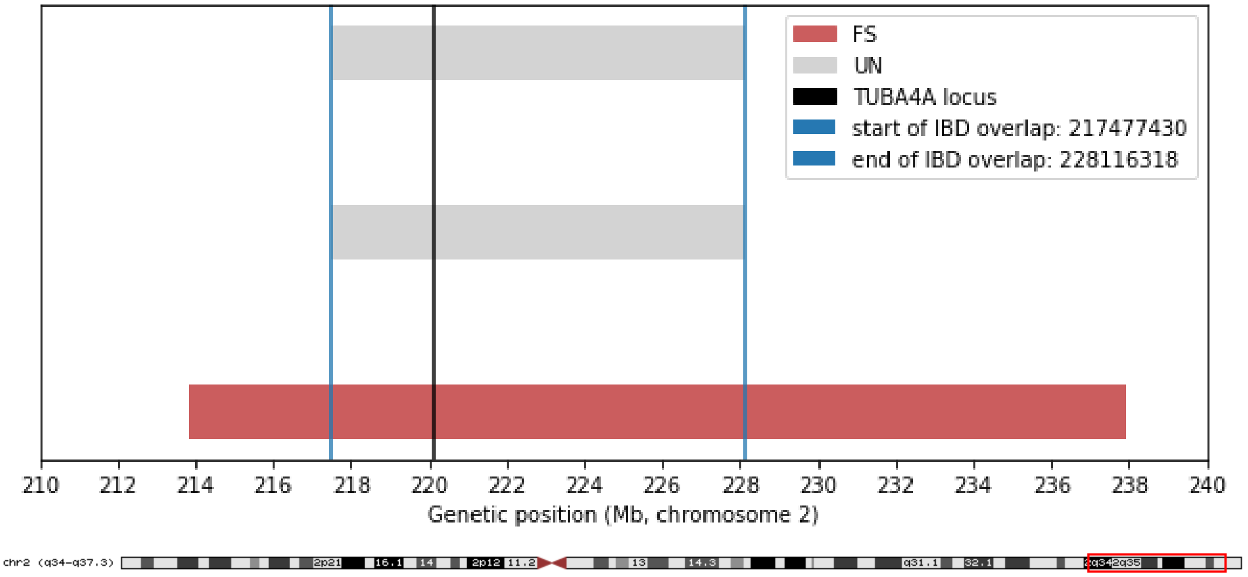

Figure S33

Pairwise identity by Descent (IBD) segments in the chromosomes that harbor TUBA4A NM_006000 c.820C>G (p.Pro274Ala) variant.

Horizontal lines represent IBD haplotypes >2cM detected using Hap-IBD. The blue vertical lines indicate the portion of IBD segments shared among all the carriers, and the black vertical line indicates the locus of the *TUBA4A* gene in chromosome 2. Each horizontal line represents a pairwise IBD segment between two carriers and is colored according to their relationship coefficient (r) calculated by KING. FS: Full siblings(r=0.5), UN: Individuals beyond 4th degree of relatedness (r<0.0625)

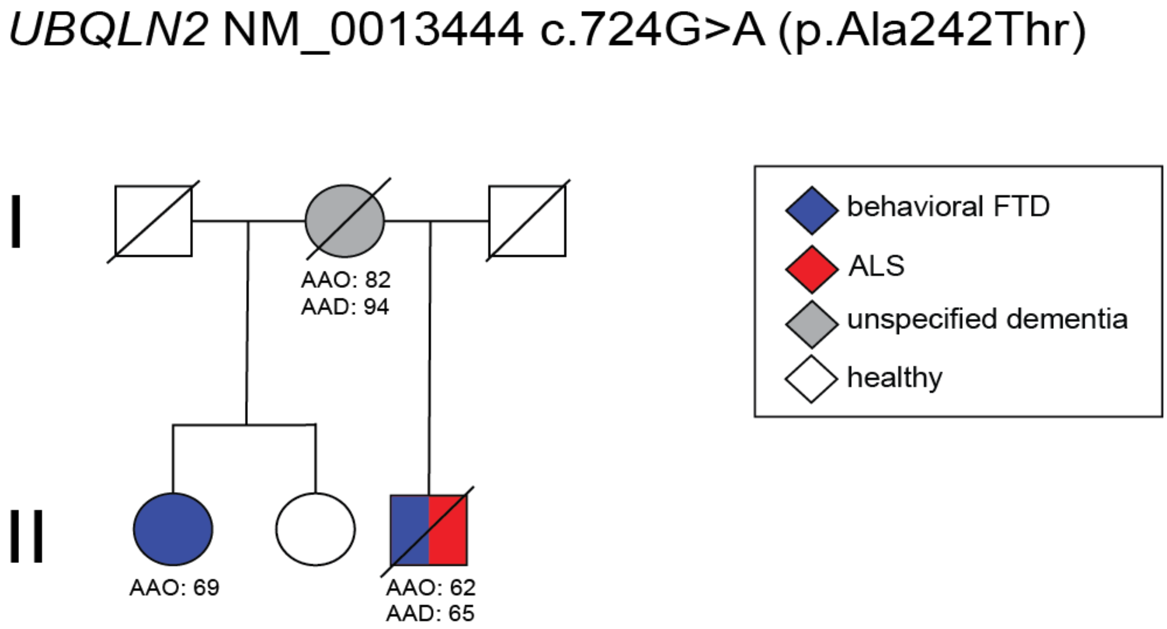

Figure S34
Pedigrees of the families with pathogenic variants in *UBQLN2* (NM_0013444) identified by the present study.
Affected individuals are represented as filled icons; empty icons represent asymptomatic individuals, numbers inside icons represent number of healthy siblings. Deceased individuals are crossed out. Females are represented by circles, and males by squares. AAO: Age at onset, AAD: Age at death, FTD: frontotemporal dementia. ALS: Amyotrophic lateral sclerosis. No cosegregation data available for this family

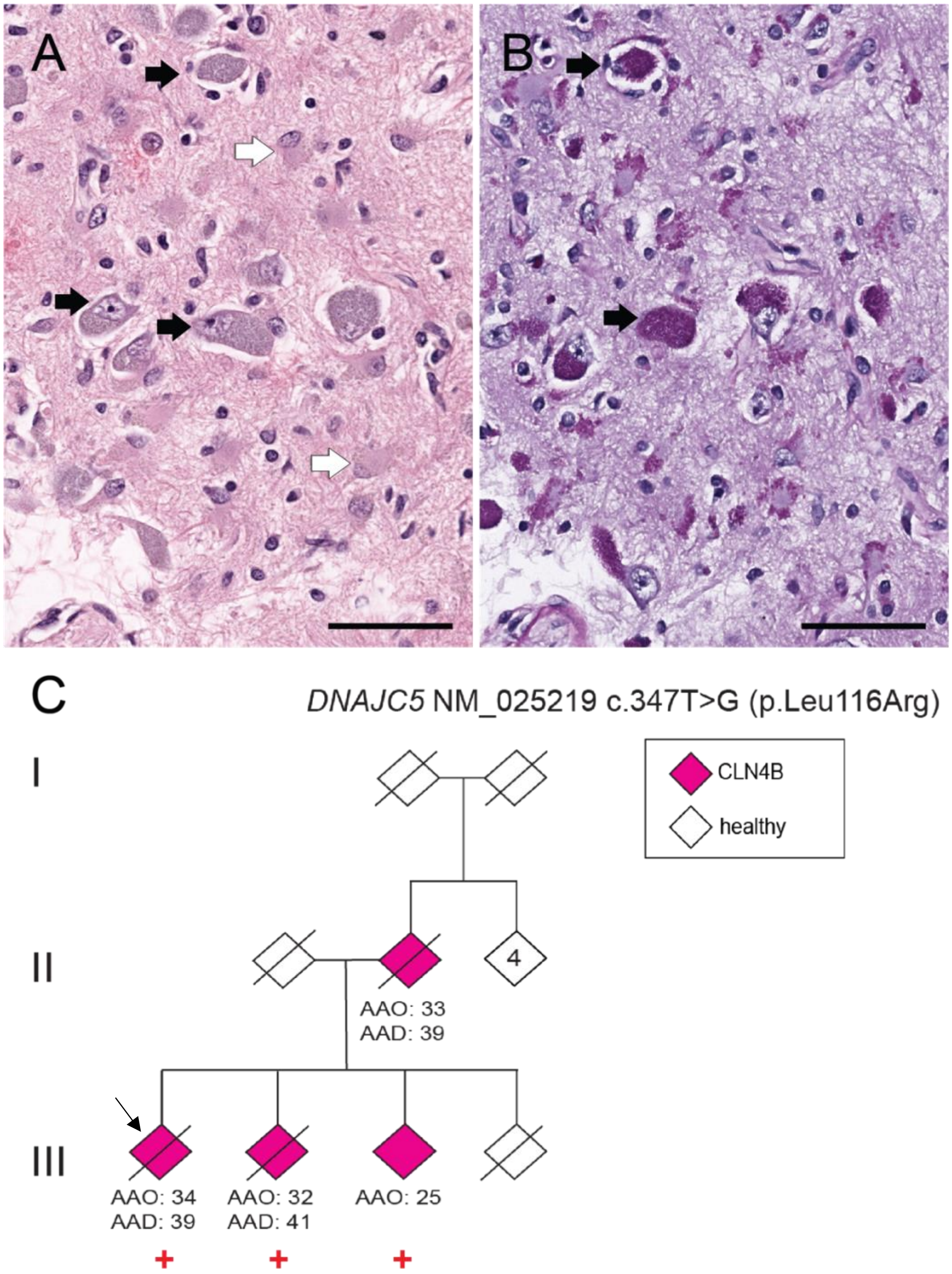

Figure S35

Top row: Histological characterization of ceroid neuronal lipofuscinosis-4B (CNL4B).

Staining with hematoxylin eosin – HE (A) and periodic acid Schiff - PAS(B). A. Foamy neuronal cytoplasmatic inclusions that displace cell nucleus (black arrows), and reactive astrocytes (white arrows) in hippocampus CA1. [Scale bar 50um] B. PAS positive neuronal cytoplasmic inclusions (black arrows) [Scale bar 50um]

Bottom row (C): Pedigree of the family

Affected individuals are represented as filled icons; empty icons represent asymptomatic individuals, numbers inside icons represent number of healthy siblings. Deceased individuals are crossed out. The gender of the individuals was omitted for confidentiality. AAO: Age at onset, AAD: Age at death. Proband is indicated with an arrow. + known carriers.

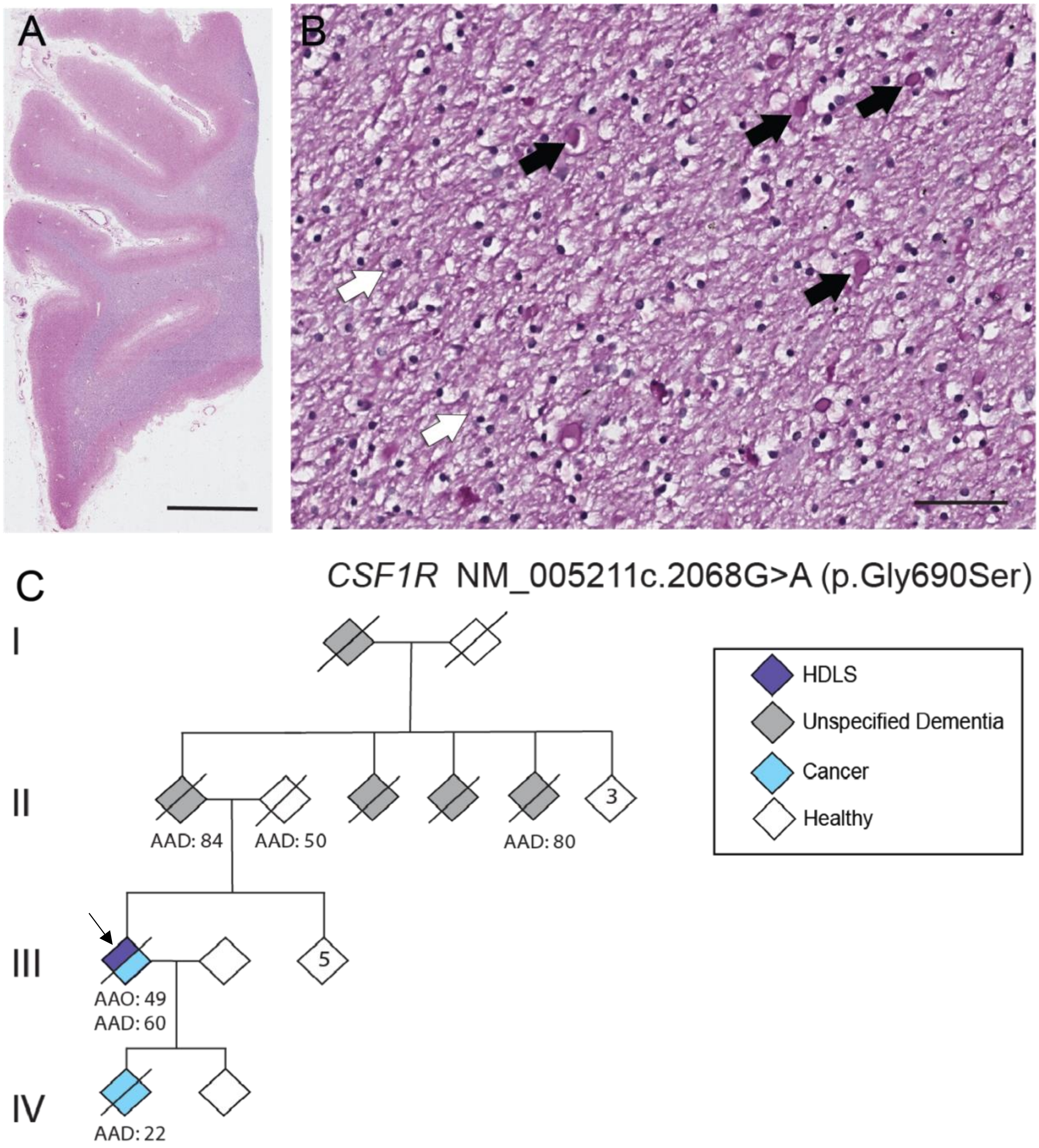

Figure S36

Top row: Histological characterization of hereditary diffuse leukoencephalopathy with spheroids (HDLS). Staining with Luxol fast blue (A) and periodic acid Schiff - PAS(B). A. Rarefied white matter and myelin loss in the inferior parietal lobe. [Scale bar 5mm] B. PAS positive axonal spheroids (black arrows) and foamy macrophages (white arrows). [Scale bar 50um]

Bottom row (C): Pedigree of the family

Affected individuals are represented as filled icons; empty icons represent asymptomatic individuals, numbers inside icons represent number of healthy siblings. Deceased individuals are crossed out. The gender of the individuals was omitted for confidentiality. AAO: Age at onset, AAD: Age at death. Proband is indicated with an arrow.

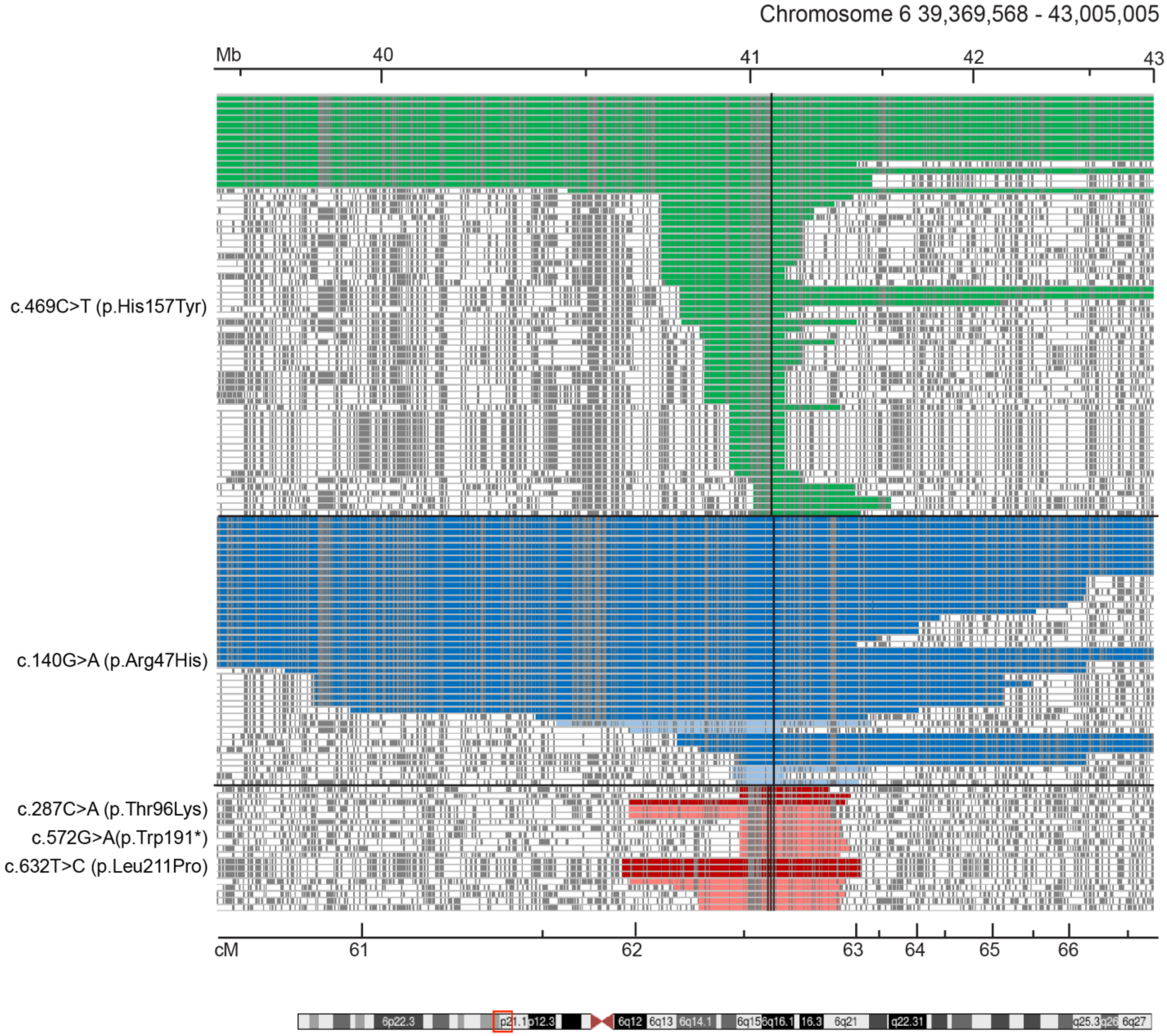

Figure S37

Alignment of the haplotypes that carry Strictly Damaging and Protein Truncating Variants in TREM2 present in more than 1 individual.

Each haplotype is represented as a row and each SNP is a column. Haplotypes are colored according the ancestral origin of the variant, showing the areas where the carriers are identical by descent (IBD). Red: African origin, Blue: European, Green: Native American. Darker shaded haplotypes correspond to individuals from the TANGL cohort, and lighter shaded haplotypes correspond to individuals from the African or European cohorts of the 1000GP. Mb: Megabases, cM: Centimorgans

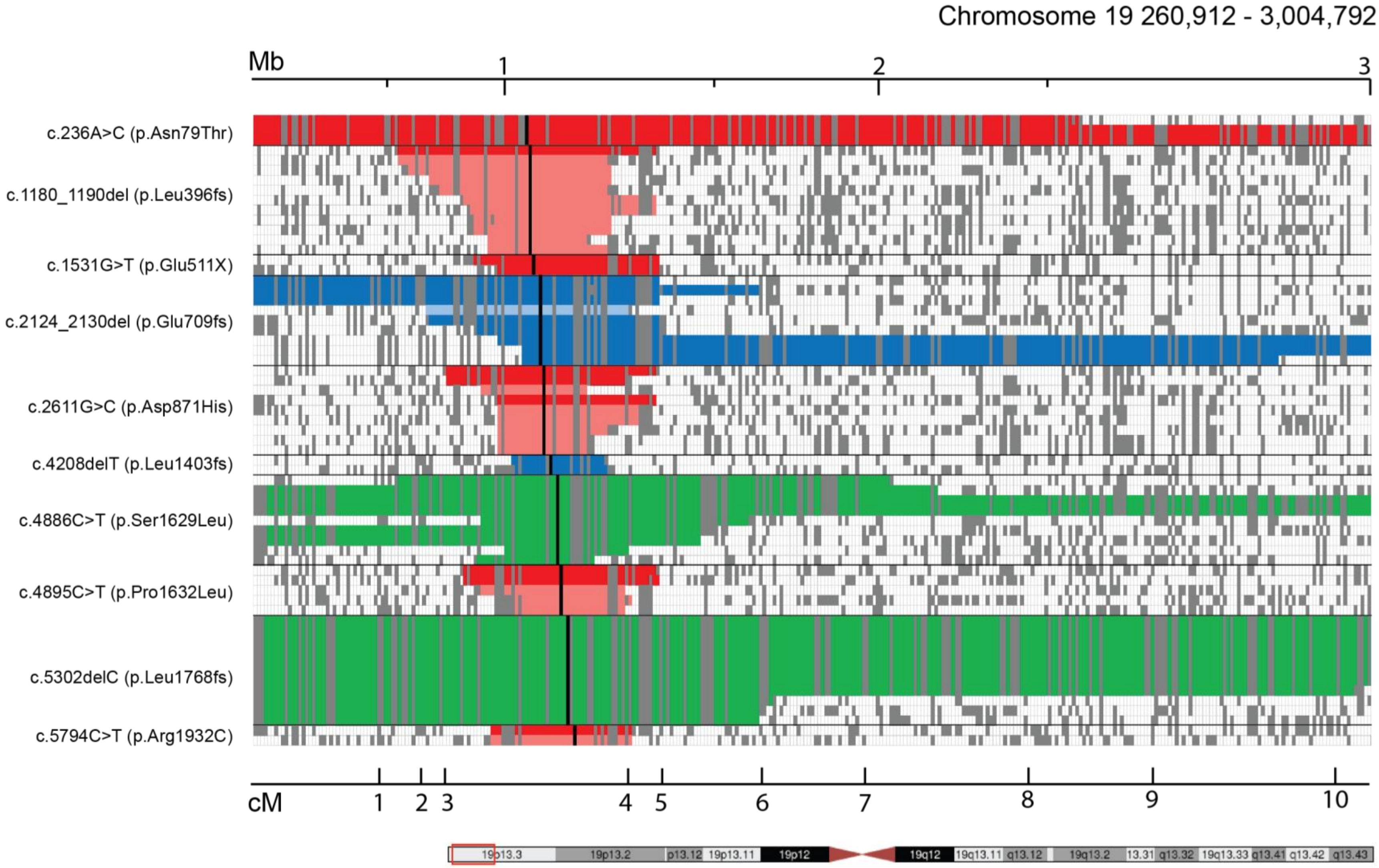

Figure S38

Alignment of the haplotypes that carry Strictly Damaging and Protein Truncating Variants in ABCA7 present in more than 1 individual.

Each haplotype is represented as a row and each SNP is a column. Haplotypes are colored according the ancestral origin of the variant, showing the areas where the carriers are identical by descent (IBD). Red: African origin, Blue: European, Green: Native American. Darker shaded haplotypes correspond to individuals from the 1TANGL cohort, and lighter shaded haplotypes correspond to individuals from the African or European cohorts of the 1000GP. Mb: Megabases, cM: Centimorgans

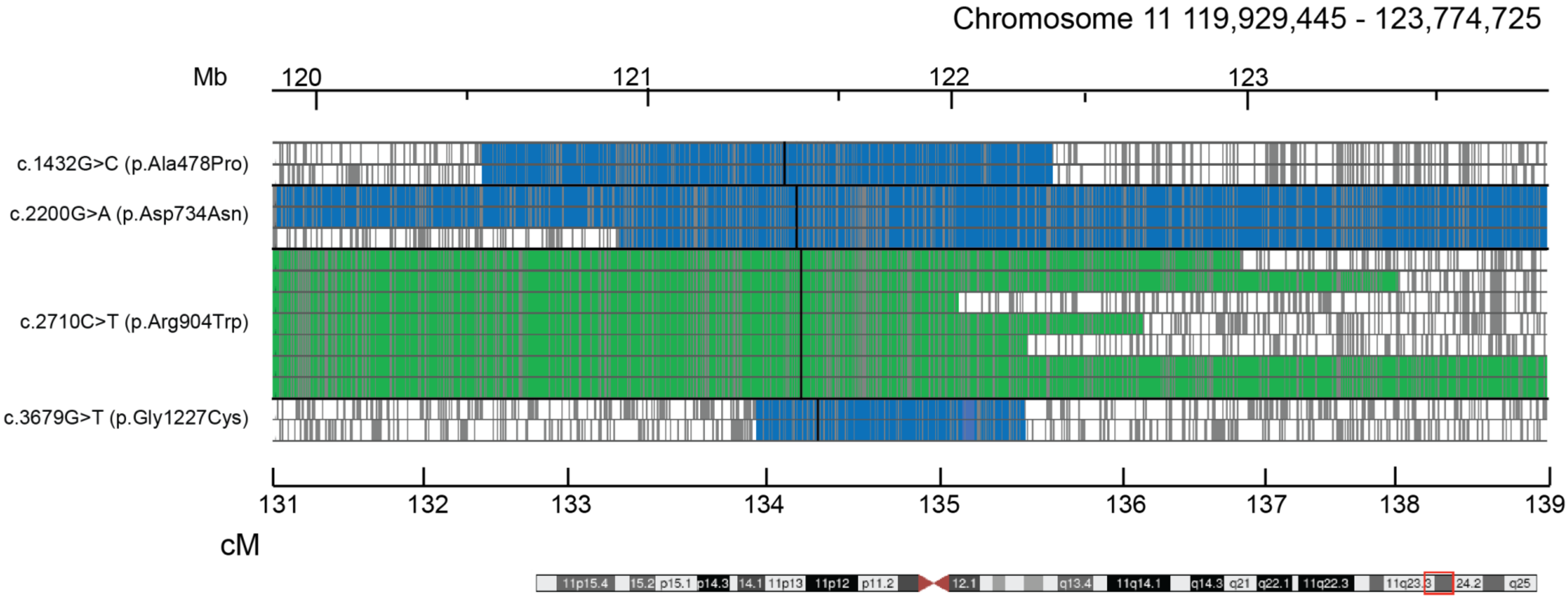

Figure S39

Alignment of the haplotypes that carry Strictly Damaging and Protein Truncating Variants in SORL1 present in more than 1 individual.

Each haplotype is represented as a row and each SNP is a column. Haplotypes are colored according the ancestral origin of the variant, showing the areas where the carriers are identical by descent (IBD). Blue: European, Green: Native American. Aligned shaded haplotypes correspond to individuals from the TANGL cohort. Mb: Megabases, cM: Centimorgans

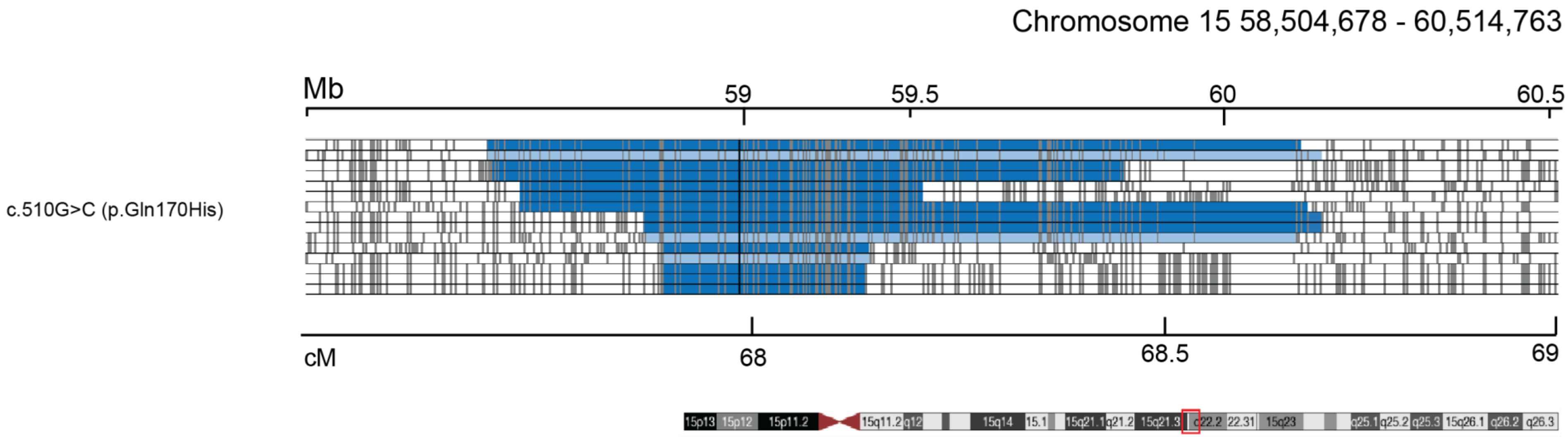

Figure S40
Alignment of the haplotypes that carry Strictly Damaging and Protein Truncating Variants in ADAM10 present in more than 1 individual.
Each haplotype is represented as a row and each SNP is a column. Haplotypes are colored according the ancestral origin of the variant, showing the areas where the carriers are identical by descent (IBD). Blue: European. Darker shaded haplotypes correspond to individuals from the TANGL cohort, and lighter shaded haplotypes correspond to individuals from the European cohort of the 1000GP. Mb: Megabases, cM: Centimorgans

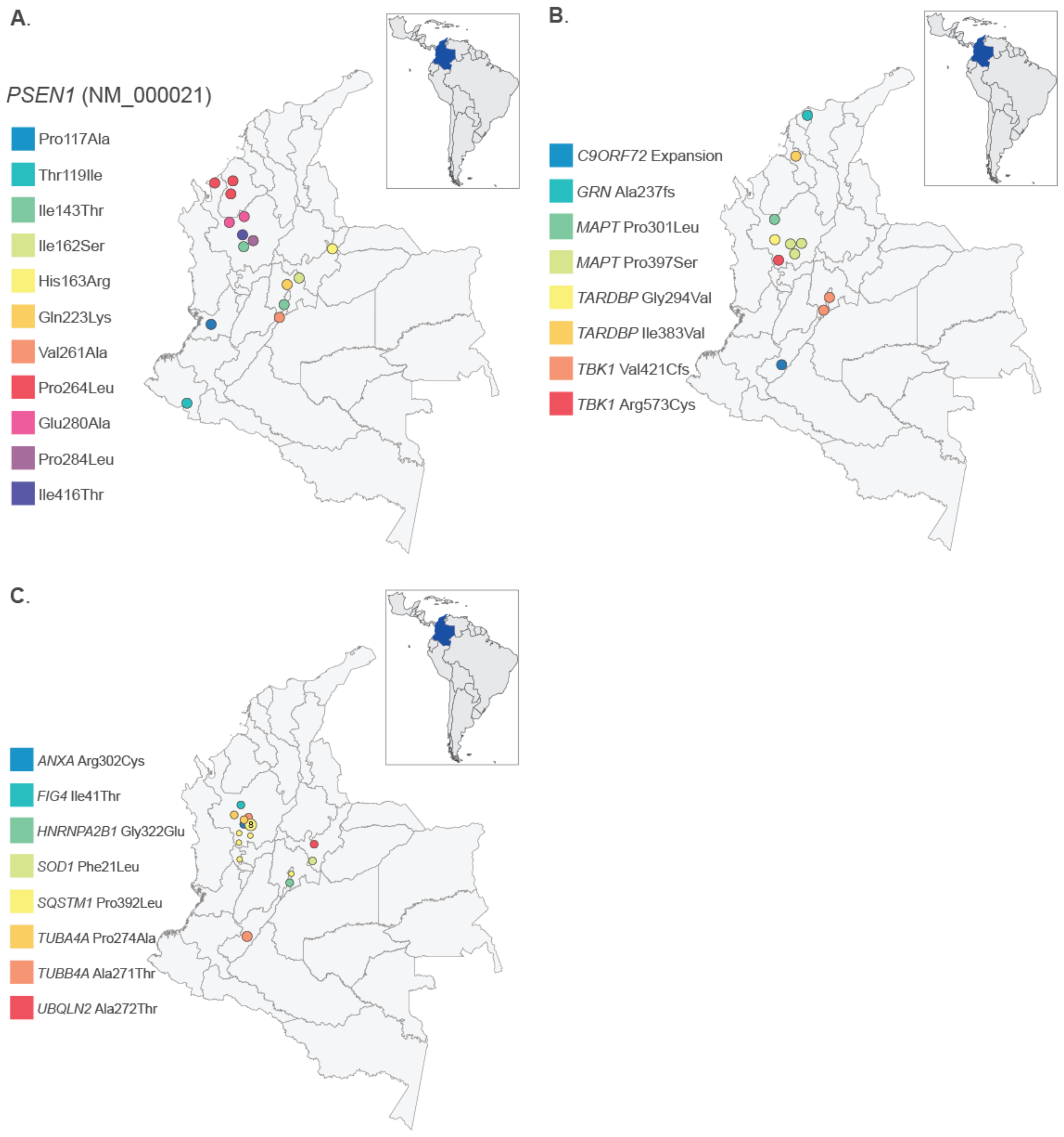

Figure S41
Maps of Colombia representing the place of origin of the families with disease causing variants. Numbers inside circles represent numbers of families carrying the variant who are original from the same geographic region.
