# Supplementary material for: A neurodegenerative disease landscape of rare mutations in Colombia due to founder effects
Source: Genome Med. 2022 Mar 8;14:27. doi: 10.1186/s13073-022-01035-9 (PMC8902761; doi:10.1186/s13073-022-01035-9)
Supplement: Supplementary file 7 — Additional file 7:. Supplementary methods. [file 13073_2022_1035_MOESM7_ESM.pdf]

ADDITIONAL FILE 7
SUPPLEMENTARY METHODS

VARIANT CURATION OF ALZHEIMER'S DISEASE ASSOCIATED GENES

- ***APP* Chr21 g.(26253828_30011000)dup.**

Duplication of the APP. Locus have been reported as disease causing for Alzheimer's Disease (AD)[1]

- ***PSEN1* NM_000021, c.349C>G (p.Pro117Ala)**

Variant previously reported in a Colombian family with early onset AD[2].

- ***PSEN1* NM_000021, c.356C>T (p. Thr119Ile) – Figure S16**

Variant previously described as pathogenic. Identified in Argentine[3] and Korean patients with AD[4]

- ***PSEN1* NM_000021, c.428T>C (p. Ile143Thr) – Figure S16**

Variant previously described as pathogenic. Reported in multiple cohorts from different ancestries. European ancestry in Belgium[5] France[6] and Sweden[7]. Asian ancestry in Japan[8] and China[9]. It had also been previously reported in another Colombian family[10].

- ***PSEN1* NM_000021, c.485T>G (p. Ile162Ser) – Figure S16**

ACMG classification: Cosegregation with disease in multiple family members – Figure S16 (ACMG criterion PP1). Absent in population databases – ExAC and 1000 Genomes project (ACMG criterion PM2). More than 300 variants in *PSEN1* have been reported and pathogenic variants in PSEN1 are the most common cause of familiar early onset Alzheimer's disease (https://www.alzforum.org/mutations/psen-1) (ACMG criterion PP2). Predicted to be damaging by three computational algorithms (SIFT CADD and PolyPhen-2) (ACMG criterion PP3) → Likely pathogenic (II)
Guerreiro algorithm for *PSEN1* and *PSEN2* variant classification[11].: Classified as Definite Pathogenic since there are more than three cases of individuals with AD carrying this *PSEN1* variant in the same family

- ***PSEN1* NM_000021, c.488A>G (p. His163Arg) – Figure S16**

Variant previously described as pathogenic. Reported in multiple cohorts from different ancestries, including Americans and Canadians[12], French[13], Japanese [14], Polish[15], Spanish[16], Turkish[17], and Chinese[18].

- ***PSEN1* NM_000021, c.667C>A (p. Gln223Lys) – Figure S16**

ACMG classification: Cosegregation with disease in multiple family members – Figure S16 (ACMG criterion PP1). Absent in population databases – ExAC and 1000 Genomes project (ACMG criterion PM2). Novel missense change at an amino acid residue where a different missense change determined to be pathogenic has been seen before[19,20](ACMG criterion PM5). More than 300 variants in *PSEN1* have been reported and pathogenic variants in PSEN1 are the most common cause of familiar early onset Alzheimer's disease (https://www.alzforum.org/mutations/psen-1) (ACMG criterion PP2). Predicted to be damaging by three computational algorithms (SIFT CADD and PolyPhen-2) (ACMG criterion PP3) → Likely pathogenic
Guerreiro algorithm for *PSEN1* and *PSEN2* variant classification[11]: Classified as Definite Pathogenic since there are more than three cases of individuals with AD carrying this *PSEN1* variant in the same family

- ***PSEN1* NM_000021, c.782C>T (p.Val261Ala)**

ACMG classification: Absent in population databases – ExAC and 1000 Genomes project (ACMG criterion PM2). Novel missense change at an amino acid residue where a different missense change determined to be pathogenic has been seen before[16,21,22] (ACMG criterion PM5). More than 300 variants in *PSEN1* have been reported and pathogenic variants in PSEN1 are the most common cause of familiar early onset Alzheimer's disease (https://www.alzforum.org/mutations/psen-1) (ACMG criterion PP2). Predicted to be damaging by three computational algorithms (SIFT CADD and PolyPhen-2) (ACMG criterion PP3) → Likely pathogenic
Guerreiro algorithm for *PSEN1* and *PSEN2* variant classification[11]: Classified as probably pathogenic as the variant is seen in a single case, it is absent from population databases, it is conserved between *PSEN1* and *PSEN2* and other pathogenic variants have been described in the same residue.

- ***PSEN1* NM_000021, c.791C>T (p. Pro264Leu) – Figure S16**

Variant previously described as pathogenic. Reported in multiple cohorts from different ancestries, including French[23], British[24] and descendants of the United Kingdom[25], Turkish[17], and Japanese[26].

- ***PSEN1* NM_000021, c.839A>C (p.Glu280Ala)**

Pathogenic variant widely described in Colombian cohorts and a Japanese family[14,27]

- ***PSEN1* NM_000021, c.851C>T (p.Pro284Leu) – Figure S16**

Variant previously described as pathogenic. Reported in a Japanese patient with spastic paraparesis and cotton wool amyloid beta plaques[28].

- ***PSEN1* NM_000021, c.1247T>C (p.Ile416Thr)**

Identified in a Colombian family with early onset AD[29].

- ***PSEN2* NM_000047, c.487C>T (p.Arg163Cys) – Figure S21**

ACMG classification: Previously described in cases with AD. While absent in controls (ACMG criterion PS4)[30]. Absent in population databases – ExAC and 1000 Genomes project (ACMG criterion PM2). Variants in *PSEN2* are a cause of genetic Alzheimer's disease (https://www.alzforum.org/mutations/psen-1) (ACMG criterion PP2). Predicted to be damaging by three computational algorithms (SIFT, CADD and PolyPhen-2) (ACMG criterion PP3) → Likely pathogenic
Guerreiro algorithm for *PSEN1* and *PSEN2* variant classification[11]: Classified as probably pathogenic as the variant is three or more unrelated cases while absent in population databases[30].

VARIANT CURATION OF FTLD-ALS ASSOCIATED GENES

- ***MAPT* NM_005910, c.902C>T (p.Pro301Leu) – Figure S23**

Variant previously described as pathogenic. Reported in multiple cohorts from different ancestries, including Dutch[31], French[32], Spanish[33], Japanese[34], and Chinese[35].

- ***MAPT NM*_005910, c c.1189C>T (p.Pro397Ser) – Figure S23**

Variant previously described as pathogenic. Reported in a cohort of Spanish ancestry[36]

- ***GRN* NM_002087, c.709-2A>G (p.Ala237fs) – Figure S29**

Variant previously described as pathogenic. Reported in multiple cohorts from different ancestries[37,38].

- ***TBK1* NM_002087, c.1257_1258del (p.Val421Cfs*26) – Figure S26**

Loss of function in *TBK1* is a known mechanism for disease[39] (ACMG criterion PVS1). Variant previously documented in Amyotrophic Lateral Sclerosis online (ALS) patients from cohort of European descent[40] (ACMG criterion PS4). Absent in population databases – ExAC and 1000 Genomes project (ACMG criterion PM2). Variant causes a premature stop codon (ACMG criterion PM4)→ Pathogenic

- ***TBK1* NM_002087, c.1717C>T (p.Arg573Cys) – Figure S26**

This variant has previously been reported as dominant non-benign in exome sequencing of ALS patients[41](ACMG criterion PS4). Located in a mutational hotspot or well-studied functional domain [42,43] (ACMG criterion PM1). Missense change at an amino acid residue where a different missense change determined to be pathogenic has been seen before[44,45] (ACMG criterion PM5). Segregation data suggests pathogenicity in homozygous carriers (ACMG criterion PP1). Predicted to be damaging by three computational algorithms (SIFT, CADD and PolyPhen-2) (ACMG criterion PP3). → Pathogenic

- ***TARDBP* NM_007375, c.881G>T (p.Gly294Val)**

Variant previously described as pathogenic for ALS. Reported in multiple cohorts from different ancestries, including Italian[36] Morrocan[47,48] and Australian[49]

- ***TARDBP* NM_007375, c.1147A>G (p.Ile383Val) – Figure S28**

Variant previously described as pathogenic. Reported in multiple cohorts from different ancestries, including Sardinian[50], Taiwanese[51], and a cohort of general European descent[40].

VARIANT CURATION OF ALS ASSOCIATED GENES

- ***ANXA11*, NM_001157 c. 904C>T (p.Arg302Cys)**

This variant been reported in individuals with ALS from different cohorts [52,53](ACMG criterion PS4). It is localized in the stabilizing N-terminal segment, where impairs protein function[54] (ACMG criterion PM1). Low rate of benign mutations in a gene associated with the phenotype[55] (ACMG criterion PP2) Predicted damaging by multiple computational methods (CADD, PolyPhen-2, and SIFT) (ACMG criterion PP3) → Pathogenic

- ***FIG4* NM_014845 c.122T>C (p.Ile41Thr)**

The Ile41Thr mutation found in this population has been the most frequent missense mutation associated with CMT4J, an autosomal recessive subtype of Charcot-Marie-Tooth disorders[56].

- ***HNRNPA2B1*, NM_001157 c.965G>A (p.Gly322Glu)**

ACMG classification:

Located in domain has been shown to lead to fibril formation and cause ALS [57,58](ACMG criterion PM1). Absent in population databases – ExAC and 1000 Genomes project (ACMG criterion PM2). Low rate of benign mutations[55] (ACMG criterion PP2). Predicted damaging by multiple computational methods (PolyPhen-2, and SIFT) (ACMG criterion PP3) → Likely Pathogenic

- ***SOD1* NM_000454, c.63C>G (p.Phe21Leu)**

Cell biology studies indicate pathogenicity of this variant [59,60](ACMG criterion PS3). Phe21Leu was reported in a study on ALS in an Italian population[61](ACMG criterion PS4) . Not present in ExAC database (ACMG criterion PM2). Missense variants at Phe21 (misreported in some early papers as being Phe20) have been reported previously in ALS patients[62](ACMG criterion PM5). Low rate of benign mutations in a gene associated with the phenotype[55](ACMG criterion PP2). Predicted damaging in two computational methods (CADD and PolyPhen-2)(ACMG criterion PP3) → Pathogenic

- ***TUBA4A* NM_006000, c.820C>G (p.Pro274Ala) – Figure S32**

Cosegregation with disease in family members of two independent families– Figure S32 (ACMG criterion PP1). Deleterious variation in this gene has been associated with sporadic and familiar ALS[63–65] (ACMG criterion PP2). Predicted damaging by two computational methods (CADD and PolyPhen-2) (ACMG criterion PP3) Absent in population databases – ExAC and 1000 Genomes project (ACMG criterion PM2). → Likely pathogenic

- ***TUBB4A* NM_006087, c.811G>A (p.Ala271Thr)**

Previously described as pathogenic for autosomal dominant motor neuron disease [Clinvar RCV000077783.3]

- ***UBQLN2* NM_001344, c.724G>A (p.Ala242Thr) – Figure 34**

Variant located in STI1 domain, demonstrated to be important for interaction with chaperone proteins and implicated in pathogenesis[66] (ACMG criterion PM1) Absent in population databases – ExAC and 1000 Genomes project (ACMG criterion PM2). Gene with low rate of benign mutations [55](ACMG criterion PP2). Predicted damaging by multiple computational methods (CADD, PolyPhen-2 and SIFT) (ACMG criterion PP3) → Likely pathogenic

VARIANT CURATION OF GENES ASSOCIATED WITH OTHER NEURODEGENERATIVE DISEASES:

- ***CSF1R* NM_001288705, c.2068G>A (p.Gly690Ser) – Figure S36**

This variant is located in the protein kinase domain, which is a hotspot for deleterious mutation [67](ACMG criterion PM1). *CSF1R* has over 60 missense variants reported as pathogenic in patients with Hereditary diffuse leukoencephalopathy with spheroids (HDLD) and cancer predisposition[55] (ACMG criterion PP2). This variant is predicted as damaging by two computational methods (CADD and PolyPhen-2) (ACMG criterion PP3). The patient's phenotype is highly specific for this gene; recurrent malignancies and early onset dementia with neuropathology confirmation of the HDLD diagnosis (ACMG criterion PP4). Family history of dementia and cancer, but with no cosegregation data available for analyses. There is not enough evidence to use the ACMG criteria to declare the variant pathogenic, but the highly specific phenotype of the patient and the family history suggests this variant to be a good candidate to test other family members.

- **DNAJC5 NM_025219, c.347T>G (p.Leu116Arg) – Figure S35**

DNAJC5 has been implicated as pathogenic for neuronal ceroid lipofuscinosis of adult onset (CLN4B) with a low rate of benign variation(ACMG criterion PP2). Functional studies of the residue Leu116 support the pathogenicity of variants in that residue[68](ACMG criterion PS3). Leu116Arg is located in a mutational hotspot for CLN4B [69–71] (ACMG criterion PM1). Leu115Arg and Leu116del mutations were originally discovered in a study done on a Czech family along with data from previous patients collected from the

Netherlands, USA, Canada, France, Italy, Germany, Austria, Belgium and Poland[72] (ACMG criterion PM5). Absent in population databases – ExAC and 1000 Genomes project (ACMG criterion PM2). Predicted to be at least possibly damaging by multiple computational methods (CADD, PolyPhen-2 and SIFT) (ACMG criterion PP3). The phenotype and cosegregation of the variant with the illness supports the diagnosis of CLN4B and pathogenicity of the variant (ACMG criteria PP4 and PP1-S) → Pathogenic

- ***LRRK2* NM_198578, c.4334C>G (p.Ser1445Cys)**

Functional studies have demonstrated that Ser1445 is one of LRRK2 autophosphorylation sites, where missense mutations are deleterious[73–75] (ACMG criterion PS3). Located in the Roc (GTP-ase like) domain which is a mutational hotspot (Uniprot database Q5S007 and [76]) (ACMG criterion PM1). Absent in population databases – ExAC and 1000 Genomes project (ACMG criterion PM2). Predicted as damaging by two computational methods (CADD and PolyPhen-2) (ACMG criterion PP3) → Likely pathogenic

- ***LRRK2* NM_198578, c.6055G>A (p.Gly2019Ser)**

The G2019S mutation has been discovered to be the most frequent cause of autosomal dominant PD in many genome and exome sequencing studies within a wide variety of populations [77–79].

REFERENCES

1. Lanoiselée H-MM, Nicolas G, Wallon D, Rovelet-Lecrux A, Lacour M, Rousseau S, et al. APP, PSEN1, and PSEN2 mutations in early-onset Alzheimer disease: A genetic screening study of familial and sporadic cases. Miller BL, editor. PLoS Med [Internet]. Public Library of Science; 2017 [cited 2018 Mar 27];14:e1002270. Available from: https://doi.org/10.1371/journal.pmed.1002270
2. Kauwe JSK, Wang J, Chakraverty S, Goate AM, Henao-Martinez AF. Novel presenilin 1 variant (P117A) causing Alzheimer's disease in the fourth decade of life. Neurosci Lett [Internet]. Elsevier; 2008 [cited 2018 Jun 18];438:257–9. Available from: https://www.sciencedirect.com/science/article/pii/S0304394008004667?via%3Dihub
3. Itzcovich T, Chrem-Méndez P, Vázquez S, Barbieri-Kennedy M, Niikado M, Martinetto H, et al. A novel mutation in PSEN1 (p.T119I) in an Argentine family with early- and late-onset Alzheimer's disease. Neurobiol Aging. Elsevier Inc.; 2020;85:155.e9-155.e12.
4. Giau V Van, Bagyinszky E, Yang YS, Youn YC, An SSA, Kim SY. Genetic analyses of early-onset Alzheimer's disease using next generation sequencing. Sci Rep [Internet]. Nature Publishing Group; 2019 [cited 2021 Sep 5];9:1–10. Available from: https://www.nature.com/articles/s41598-019-44848-2
5. Cruts M, Backhovens H, Wang SY, Van Gassen G, Theuns J, De Jonghe C, et al. Molecular genetic analysis of familial early-onset alzheimer's disease linked to chromosome 14q24.3. Hum Mol Genet. 1995;
6. Raux G, Guyant-Maréchal L, Martin C, Bou J, Penet C, Brice A, et al. Molecular diagnosis of autosomal dominant early onset Alzheimer's disease: An update [Internet]. J. Med. Genet. J Med Genet; 2005 [cited 2021 Sep 20]. p. 793–5. Available from: https://pubmed.ncbi.nlm.nih.gov/16033913/
7. Keller L, Welander H, Chiang HH, Tjernberg LO, Nennesmo I, Wallin ÅK, et al. The PSEN1 I143T mutation in a Swedish family with Alzheimer's disease: Clinical report and quantification of AB in different brain regions. Eur J Hum Genet. 2010;
8. Arai N, Kishino A, Takahashi Y, Morita D, Nakamura K, Yokoyama T, et al. Familial cases presenting very early onset autosomal dominant Alzheimer's disease with I143T in presenilin-1 gene: Implication for genotype-phenotype correlation [1]. Neurogenetics. 2008. p. 65–7.
9. Xu Y, Liu X, Shen J, Tian W, Fang R, Li B, et al. The whole exome sequencing clarifies the genotype-phenotype correlations in patients with early-onset dementia. Aging Dis. International Society on Aging and Disease; 2018;9:696–705.

10. Arango D, Cruts M, Torres O, Backhovens H, Serrano ML, Villareal E, et al. Systematic genetic study of Alzheimer disease in Latin America: Mutation frequencies of the amyloid β precursor protein and presenilin genes in Colombia. Am J Med Genet [Internet]. Wiley-Blackwell; 2001 [cited 2018 Mar 17];103:138–43. Available from: http://doi.wiley.com/10.1002/1096-8628%2820011001%29103%3A2%3C138%3A%3AAID-AJMG1529%3E3.0.CO%3B2-8
11. Guerreiro RJ, Baquero M, Blesa R, Boada M, Brás JM, Bullido MJ, et al. Genetic screening of Alzheimer's disease genes in Iberian and African samples yields novel mutations in presenilins and APP. Neurobiol Aging [Internet]. Elsevier; 2010 [cited 2018 Mar 16];31:725–31. Available from: https://www.sciencedirect.com/science/article/pii/S0197458008002194#fig2
12. Sherrington R, Rogaev EI, Liang Y, Rogaeva EA, Levesque G, Ikeda M, et al. Cloning of a gene bearing missense mutations in early-onset familial Alzheimer's disease. Nature. 1995;
13. Campion D, Dumanchin C, Hannequin D, Dubois B, Belliard S, Puel M, et al. Early-onset autosomal dominant Alzheimer disease: prevalence, genetic heterogeneity, and mutation spectrum. Am J Hum Genet. 1999;
14. Tanahashi H, Kawakatsu S, Kaneko M, Yamanaka H, Takahashi K, Tabira T. Sequence analysis of presenilin-1 gene mutation in Japanese Alzheimer's disease patients. Neurosci Lett. 1996;218:139–41.
15. C. Z, M. S, B. P, T. G, D. R, J. I, et al. Mutations in presenilin 1, presenilin 2 and amyloid precursor protein genes in patients with early-onset Alzheimer's disease in Poland. Exp Neurol. 2003;
16. Gómez-Tortosa E, Barquero S, Barón M, Gil-Neciga E, Castellanos F, Zurdo M, et al. Clinical-genetic correlations in familial Alzheimer's disease caused by presenilin 1 mutations. J Alzheimers Dis [Internet]. 2010 [cited 2018 Jul 24];19:873–84. Available from: http://www.ncbi.nlm.nih.gov/pubmed/20157243
17. Lohmann E, Guerreiro RJ, Erginel-Unaltuna N, Gurunlian N, Bilgic B, Gurvit H, et al. Identification of PSEN1 and PSEN2 gene mutations and variants in Turkish dementia patients. Neurobiol Aging. Elsevier Inc.; 2012;33:1850.e17-1850.e27.
18. Shea YF, Chu LW, Chan AOK, Ha J, Li Y, Song YQ. A systematic review of familial Alzheimer's disease: Differences in presentation of clinical features among three mutated genes and potential ethnic differences. J. Formos. Med. Assoc. Elsevier B.V.; 2016. p. 67–75.
19. Uttner I, Kirchheiner J, Tumani H, Mottaghy FM, Lebedeva E, Özer E, et al. A novel presenilin1 mutation (Q223R) associated with early onset Alzheimer's disease, dysarthria and spastic paraparesis and decreased Abeta levels in CSF. Eur J Neurol [Internet]. Eur J Neurol; 2010 [cited 2021 Oct 5];17:631–3. Available from: https://pubmed.ncbi.nlm.nih.gov/19912322/
20. Wallon D, Rousseau S, Rovelet-Lecrux A, Quillard-Muraine M, Guyant-Maréchal L, Martinaud O, et al. The french series of autosomal dominant early onset alzheimer's disease cases: Mutation spectrum and cerebrospinal fluid biomarkers. J Alzheimer's Dis [Internet]. J Alzheimers Dis; 2012 [cited 2021 Oct 5];30:847–56. Available from: https://pubmed.ncbi.nlm.nih.gov/22475797/
21. Rogaeva EA, Fafel KC, Song YQ, Medeiros H, Sato C, Liang Y, et al. Screening for PS1 mutations in a referral-based series of AD cases: 21 Novel mutations. Neurology. Lippincott Williams and Wilkins; 2001;57:621–5.
22. Miravalle L, Calero M, Takao M, Roher AE, Ghetti B, Vidal R. Amino-terminally truncated Aβ peptide species are the main component of cotton wool plaques. Biochemistry [Internet]. Biochemistry; 2005 [cited 2021 Oct 5];44:10810–21. Available from: https://pubmed.ncbi.nlm.nih.gov/16086583/
23. Campion D, Dumanchin C, Hannequin D, Dubois B, Belliard S, Puel M, et al. Early-onset autosomal dominant Alzheimer disease: Prevalence, genetic heterogeneity, and mutation spectrum. Am J Hum Genet. University of Chicago Press; 1999;65:664–70.
24. Poorkaj P, Sharma V, Anderson L, Nemens E, Alonso ME, Orr H, et al. Missense mutations in the chromosome 14 familial Alzheimer's disease presenilin 1 gene. Hum Mutat. 1998;11:216–21.
25. Mahoney CJ, Downey LE, Beck J, Liang Y, Mead S, Perry RJ, et al. The presenilin 1 P264L mutation presenting as non-fluent/agrammatic primary progressive aphasia. J Alzheimer's Dis [Internet]. J

Alzheimers Dis; 2013 [cited 2021 Sep 20];36:239–43. Available from: https://pubmed.ncbi.nlm.nih.gov/23579325/

26. Ishizuka T, Nakamura M, Ichiba M, Fujita S, Takeuchi K, Fujimoto T, et al. Different clinical phenotypes in siblings with a Presenilin-1 P264L mutation. Dement Geriatr Cogn Disord. 2012;33:132–40.

27. Lopera F. Clinical features of early-onset Alzheimer disease in a large kindred with an E280A presenilin-1 mutation. JAMA J Am Med Assoc [Internet]. 1997;277:793–9. Available from: http://jama.ama-assn.org/cgi/doi/10.1001/jama.277.10.793

28. Tabira T, De Chui H, Nakayama H, Kuroda S, Shibuya M. Alzheimer's disease with spastic paresis and cotton wool type plaques [Internet]. J. Neurosci. Res. Wiley-Blackwell; 2002 [cited 2018 Sep 7]. p. 367–72. Available from: http://doi.wiley.com/10.1002/jnr.10392

29. Ramirez Aguilar L, Acosta-Uribe J, Giraldo MM, Moreno S, Baena A, Alzate D, et al. Genetic origin of a large family with a novel PSEN1 mutation (Ile416Thr). Alzheimers Dement [Internet]. John Wiley & Sons, Ltd; 2019 [cited 2021 Jul 20];15:709–19. Available from: https://alz-journals.onlinelibrary.wiley.com/doi/10.1016/j.jalz.2018.12.010

30. Gao Y, Ren RJ, Zhong ZL, Dammer E, Zhao QH, Shan S, et al. Mutation profile of APP, PSEN1, and PSEN2 in Chinese familial Alzheimer's disease. Neurobiol Aging [Internet]. Elsevier Inc.; 2019 [cited 2020 Oct 19];77:154–7. Available from: https://linkinghub.elsevier.com/retrieve/pii/S0197458019300326

31. Hutton M, Lendon CL, Rizzu P, Baker M, Froelich S, Houlden HH, et al. Association of missense and 5'-splice-site mutations in tau with the inherited dementia FTDP-17. Nature. Nature; 1998;393:702–4.

32. Dumanchin C, Camuzat A, Campion D, Verpillat P, Hannequin D, Dubois B, et al. Segregation of a missense mutation in the microtubule-associated protein tau gene with familial frontotemporal dementia and parkinsonism. Hum Mol Genet. Hum Mol Genet; 1998;7:1825–9.

33. Borrego-Écija S, Morgado J, Palencia-Madrid L, Grau-Rivera O, Reñé R, Hernández I, et al. Frontotemporal Dementia Caused by the P301L Mutation in the MAPT Gene: Clinicopathological Features of 13 Cases from the Same Geographical Origin in Barcelona, Spain. Dement Geriatr Cogn Disord. S. Karger AG; 2017;44:213–21.

34. Ishizuka T, Nakamura M, Ichiba M, Sano A. Familial semantic dementia with P301L mutation in the tau gene. Dement Geriatr Cogn Disord. Dement Geriatr Cogn Disord; 2011;31:334–40.

35. He S, Chen S, Xia MR, Sun ZK, Huang Y, Zhang JW. The role of MAPT gene in Chinese dementia patients: A P301l pedigree study and brief literature review. Neuropsychiatr Dis Treat. Dove Medical Press Ltd.; 2018;14:1627–33.

36. Borrego-Écija S, Antonell A, Puig-Butillé JA, Pericot I, Prat-Bravo C, Abellan-Vidal MT, et al. Novel P397S MAPT variant associated with late onset and slow progressive frontotemporal dementia. Ann Clin Transl Neurol. Wiley-Blackwell; 2019;6:1559–65.

37. Leverenz JB, Yu CE, Montine TJ, Steinbart E, Bekris LM, Zabetian C, et al. A novel progranulin mutation associated with variable clinical presentation and tau, TDP43 and alpha-synuclein pathology. Brain. Oxford Academic; 2007;130:1360–74.

38. Sassi C, Capozzo R, Gibbs R, Crews C, Zecca C, Arcuti S, et al. A Novel Splice-Acceptor Site Mutation in GRN (c.709-2 A>T) Causes Frontotemporal Dementia Spectrum in a Large Family from Southern Italy. J Alzheimer's Dis. IOS Press; 2016;53:475–85.

39. Verheijen J, van der Zee J, Gijselinck I, Van den Bossche T, Dillen L, Heeman B, et al. Common and rare TBK1 variants in early-onset Alzheimer disease in a European cohort. Neurobiol Aging [Internet]. Elsevier; 2018 [cited 2019 Mar 13];62:245.e1-245.e7. Available from: https://linkinghub.elsevier.com/retrieve/pii/S0197458017303512

40. Ramos EM, Dokuru DR, Van Berlo V, Wojta K, Wang Q, Huang AY, et al. Genetic screening of a large series of North American sporadic and familial frontotemporal dementia cases. Alzheimer's Dement. John Wiley and Sons Inc.; 2020;16:118–30.

41. Cirulli ET, Lasseigne BN, Petrovski S, Sapp PC, Dion PA, Leblond CS, et al. Exome sequencing in

amyotrophic lateral sclerosis identifies risk genes and pathways. Science (80-) [Internet]. 2015;347:1436–1441. Available from: http://www.ncbi.nlm.nih.gov/pubmed/25700176
42. Zhang C, Shang G, Gui X, Zhang X, Bai X chen, Chen ZJ. Structural basis of STING binding with and phosphorylation by TBK1. Nature [Internet]. Nature; 2019 [cited 2021 Oct 5];567:394–8. Available from: https://pubmed.ncbi.nlm.nih.gov/30842653/
43. Larabi A, Devos JM, Ng SL, Nanao MH, Round A, Maniatis T, et al. Crystal Structure and Mechanism of Activation of TANK-Binding Kinase 1. Cell Rep [Internet]. Cell Rep; 2013 [cited 2021 Oct 5];3:734–46. Available from: https://pubmed.ncbi.nlm.nih.gov/23453971/
44. Cirulli ET, Lasseigne BN, Petrovski S, Sapp PC, Dion PA, Leblond CS, et al. Exome sequencing in amyotrophic lateral sclerosis identifies risk genes and pathways. Science (80-) [Internet]. Science; 2015 [cited 2021 Oct 5];347:1436–41. Available from: https://pubmed.ncbi.nlm.nih.gov/25700176/
45. Gómez-Tortosa E, Van der Zee J, Ruggiero M, Gijselinck I, Esteban-Pérez J, García-Redondo A, et al. Familial primary lateral sclerosis or dementia associated with Arg573Gly TBK1 mutation. J Neurol Neurosurg Psychiatry [Internet]. J Neurol Neurosurg Psychiatry; 2017 [cited 2021 Oct 5];88:996–7. Available from: https://pubmed.ncbi.nlm.nih.gov/28365590/
46. Conforti FL, Sproviero W, Simone IL, Mazzei R, Valentino P, Ungaro C, et al. TARDBP gene mutations in south Italian patients with amyotrophic lateral sclerosis. J Neurol Neurosurg Psychiatry. BMJ Publishing Group Ltd; 2011;82:587–8.
47. Corrado L, Ratti A, Gellera C, Buratti E, Castellotti B, Carlomagno Y, et al. High frequency of TARDBP gene mutations in Italian patients with amyotrophic lateral sclerosis. Hum Mutat [Internet]. 2009;30:688–94. Available from: http://doi.wiley.com/10.1002/humu.20950
48. Corrado L, Pensato V, Croce R, Di Pierro A, Mellone S, Dalla Bella E, et al. The first case of the TARDBP p.G294V mutation in a homozygous state: is a single pathogenic allele sufficient to cause ALS? Amyotroph Lateral Scler Front Degener. Taylor and Francis Ltd; 2020;21:273–9.
49. Williams KL, Durnall JC, Thoeng AD, Warraich ST, Nicholson GA, Blair IP. A novel TARDBP mutation in an Australian amyotrophic lateral sclerosis kindred. J Neurol Neurosurg Psychiatry. 2009;
50. Floris G, Borghero G, Cannas A, Di Stefano F, Murru MR, Corongiu D, et al. Clinical phenotypes and radiological findings in frontotemporal dementia related to TARDBP mutations. J Neurol. Dr. Dietrich Steinkopff Verlag GmbH and Co. KG; 2015;262:375–84.
51. Cheng Y-W, Lee M-J, Chen T-F, Cheng T-W, Lai Y-M, Hua M-S, et al. A single nucleotide TDP-43 mutation within a Taiwanese family: A multifaceted demon. Amyotroph Lateral Scler Front Degener [Internet]. Taylor and Francis Ltd; 2016 [cited 2020 Jul 2];17:292–4. Available from: https://www.tandfonline.com/doi/abs/10.3109/21678421.2015.1111905
52. Topp SD, Fallini C, Shibata H, Chen HJ, Troakes C, King A, et al. Mutations in the vesicular trafficking protein Annexin A11 are associated with amyotrophic lateral sclerosis Bradley N. Smith. Sci Transl Med. American Association for the Advancement of Science; 2017;9.
53. Zhang K, Liu Q, Liu K, Shen D, Tai H, Shu S, et al. ANXA11 mutations prevail in Chinese ALS patients with and without cognitive dementia. Neurol Genet. Lippincott Williams and Wilkins; 2018;4.
54. Lillebostad PAG, Raasakka A, Hjellbrekke SJ, Patil S, Røstbø T, Hollås H, et al. Structure of the als mutation target annexin a11 reveals a stabilising n-terminal segment. Biomolecules [Internet]. Multidisciplinary Digital Publishing Institute (MDPI); 2020 [cited 2021 Oct 4];10. Available from: /pmc/articles/PMC7226064/
55. Stenson PD, Mort M, Ball E V., Evans K, Hayden M, Heywood S, et al. The Human Gene Mutation Database: towards a comprehensive repository of inherited mutation data for medical research, genetic diagnosis and next-generation sequencing studies. Hum Genet [Internet]. Springer Verlag; 2017 [cited 2021 Jan 10];136:665–77. Available from: https://www.ncbi.nlm.nih.gov/pmc/articles/PMC5429360/
56. Lenk GM, Ferguson CJ, Chow CY, Jin N, Jones JM, Grant AE, et al. Pathogenic mechanism of the FIG4 mutation responsible for charcot-marie-tooth disease CMT4J. PLoS Genet [Internet]. PLoS Genet; 2011

[cited 2020 Aug 10];7. Available from: https://pubmed.ncbi.nlm.nih.gov/21655088/
57. Kim HJ, Kim NC, Wang YD, Scarborough EA, Moore J, Diaz Z, et al. Mutations in prion-like domains in hnRNPA2B1 and hnRNPA1 cause multisystem proteinopathy and ALS. Nature [Internet]. Nature; 2013 [cited 2021 Oct 5];495:467–73. Available from: https://pubmed.ncbi.nlm.nih.gov/23455423/
58. Xiang S, Kato M, Wu LC, Lin Y, Ding M, Zhang Y, et al. The LC Domain of hnRNPA2 Adopts Similar Conformations in Hydrogel Polymers, Liquid-like Droplets, and Nuclei. Cell [Internet]. Cell; 2015 [cited 2021 Oct 5];163:829–39. Available from: https://pubmed.ncbi.nlm.nih.gov/26544936/
59. Gregoire S, Zhang S, Costanzo J, Wilson K, Fernandez EJ, Kwon I. Cis-suppression to arrest protein aggregation in mammalian cells. Biotechnol Bioeng [Internet]. Biotechnol Bioeng; 2014 [cited 2021 Oct 5];111:462–74. Available from: https://pubmed.ncbi.nlm.nih.gov/24114411/
60. Proctor EA, Fee L, Tao Y, Redler RL, Fay JM, Zhang Y, et al. Nonnative SOD1 trimer is toxic to motor neurons in a model of amyotrophic lateral sclerosis. Proc Natl Acad Sci U S A [Internet]. Proc Natl Acad Sci U S A; 2016 [cited 2021 Oct 5];113:614–9. Available from: https://pubmed.ncbi.nlm.nih.gov/26719414/
61. Lattante S, Conte A, Zollino M, Luigetti M, Del Grande A, Marangi G, et al. Contribution of major amyotrophic lateral sclerosis genes to the etiology of sporadic disease. Neurology [Internet]. Lippincott Williams and Wilkins; 2012 [cited 2020 Dec 13];79:66–72. Available from: http://www.neurology.org/cgi/doi/10.1212/WNL.0b013e31825dceca
62. Andersen PM, Sims KB, Xin WW, Kiely R, O'Neill G, Ravits J, et al. Sixteen novel mutations in the Cu/Zn superoxide dismutase gene in amyotrophic lateral sclerosis: A decade of discoveries, defects and disputes [Internet]. Amyotroph. Lateral Scler. Other Mot. Neuron Disord. 2003 [cited 2020 Dec 13]. p. 62–73. Available from: https://www.tandfonline.com/action/journalInformation?journalCode=iafd20
63. Pensato V, Tiloca C, Corrado L, Bertolin C, Sardone V, Del Bo R, et al. TUBA4A gene analysis in sporadic amyotrophic lateral sclerosis: identification of novel mutations [Internet]. J. Neurol. Springer; 2015 [cited 2021 Oct 5]. p. 1376–8. Available from: https://link-springer-com.proxy.library.ucsb.edu:9443/article/10.1007/s00415-015-7739-y
64. Li J, He J, Tang L, Chen L, Ma Y, Fan D. Screening for TUBA4A mutations in a large Chinese cohort of patients with ALS: Re-evaluating the pathogenesis of TUBA4A in ALS. J Neurol Neurosurg Psychiatry [Internet]. BMJ Publishing Group Ltd; 2018 [cited 2021 Oct 5];89:1350–2. Available from: https://jnnp-bmj-com.proxy.library.ucsb.edu:9443/content/89/12/1350
65. Perrone F, Nguyen HP, Van Mossevelde S, Moisse M, Sieben A, Santens P, et al. Investigating the role of ALS genes CHCHD10 and TUBA4A in Belgian FTD-ALS spectrum patients. Neurobiol Aging [Internet]. Elsevier; 2017 [cited 2019 Sep 15];51:177.e9-177.e16. Available from: https://linkinghub.elsevier.com/retrieve/pii/S0197458016303141
66. Renaud L, Picher-Martel V, Codron P, Julien JP. Key role of UBQLN2 in pathogenesis of amyotrophic lateral sclerosis and frontotemporal dementia [Internet]. Acta Neuropathol. Commun. BioMed Central Ltd.; 2019 [cited 2020 Jun 18]. p. 103. Available from: https://actaneurocomms.biomedcentral.com/articles/10.1186/s40478-019-0758-7
67. Luo J, Elwood F, Britschgi M, Villeda S, Zhang H, Ding Z, et al. Colony-stimulating factor 1 receptor (CSF1R) signaling in injured neurons facilitates protection and survival. J Exp Med [Internet]. 2013;210:157–172. Available from: https://www.ncbi.nlm.nih.gov/pubmed/23296467
68. Greaves J, Lemonidis K, Gorleku OA, Cruchaga C, Grefen C, Chamberlain LH. Palmitoylation-induced Aggregation of Cysteine-string Protein Mutants That Cause Neuronal Ceroid Lipofuscinosis. J Biol Chem [Internet]. American Society for Biochemistry and Molecular Biology; 2012 [cited 2021 Oct 6];287:37330. Available from: /pmc/articles/PMC3481330/
69. Velinov M, Dolzhanskaya N, Gonzalez M, Powell E, Konidari I, Hulme W, et al. Mutations in the gene DNAJC5 cause autosomal dominant kufs disease in a proportion of cases: Study of the parry family and 8 other families. PLoS One [Internet]. PLoS One; 2012 [cited 2021 Oct 6];7. Available from: https://pubmed.ncbi.nlm.nih.gov/22235333/

70. Cadieux-Dion M, Andermann E, Lachance-Touchette P, Ansorge O, Meloche C, Barnabé A, et al. Recurrent mutations in DNAJC5 cause autosomal dominant Kufs disease. Clin Genet [Internet]. Clin Genet; 2013 [cited 2021 Oct 6];83:571–5. Available from: https://pubmed.ncbi.nlm.nih.gov/22978711/
71. Burneo JG, Arnold T, Palmer CA, Kuzniecky RI, Oh SJ, Faught E. Adult-onset neuronal ceroid lipofuscinosis (Kufs disease) with autosomal dominant inheritance in Alabama. Epilepsia [Internet]. Epilepsia; 2003 [cited 2021 Oct 6];44:841–6. Available from: https://pubmed.ncbi.nlm.nih.gov/12790899/
72. Nosková L, Stránecký V, Hartmannová H, Přistoupilová A, Barešová V, Ivánek R, et al. Mutations in DNAJC5, encoding cysteine-string protein alpha, cause autosomal-dominant adult-onset neuronal ceroid lipofuscinosis. Am J Hum Genet [Internet]. Am J Hum Genet; 2011 [cited 2021 Oct 6];89:241–52. Available from: https://pubmed.ncbi.nlm.nih.gov/21820099/
73. Edelman W. Identification of candidate substrates of the leucine rich repeat kinase 2 by mass spectrometry-based phosphoproteomics [Internet]. undefined. [Seattle]: University of Washington; 2016 [cited 2021 Oct 6]. Available from: http://hdl.handle.net/1773/38145
74. Reynolds A, Doggett EA, Riddle SM, Lebakken CS, Jeremy Nichols R. LRRK2 kinase activity and biology are not uniformly predicted by its autophosphorylation and cellular phosphorylation site status. Front Mol Neurosci. Frontiers; 2014;7:54.
75. Christensen K, Hentzer M, Oppermann F, Elschenbroich S, Dossang P, Thirstrup K, et al. LRRK2 exonic variants associated with Parkinson's disease augment phosphorylation levels for LRRK2-Ser1292 and Rab10-Thr73. bioRxiv [Internet]. 2018 [cited 2021 Oct 6];447946. Available from: https://doi.org/10.1101/447946
76. Refai FS, Ng SH, Tan EK. Evaluating LRRK2 genetic variants with unclear pathogenicity. Biomed Res Int. Hindawi Publishing Corporation; 2015;2015.
77. Ross OA, Toft M, Farrer MJ, Papapetropoulos S, Singer C, Mash DC, et al. Clinical Heterogeneity of the LRRK2 G2019S Mutation. Arch Neurol [Internet]. American Medical Association; 2006 [cited 2018 Aug 27];63:1242. Available from: http://archneur.jamanetwork.com/article.aspx?doi=10.1001/archneur.63.9.1242
78. Cornejo-Olivas M, Torres L, Velit-Salazar MR, Inca-Martinez M, Mazzetti P, Cosentino C, et al. Variable frequency of LRRK2 variants in the Latin American research consortium on the genetics of Parkinson's disease (LARGE-PD), a case of ancestry /692/617/375/1718 /631/208/1516 article. npj Park Dis. Nature Publishing Group; 2017;3.
79. Zabetian CP, Lauricella CJ, Tsuang DW, Leverenz JB, Schellenberg GD, Payami H. Analysis of the LRRK2 G2019S mutation in Alzheimer disease [3] [Internet]. Arch. Neurol. NIH Public Access; 2006 [cited 2018 Aug 27]. p. 156–7. Available from: http://www.ncbi.nlm.nih.gov/pubmed/16401756
